# The microbiome of marine mat-forming cyanobacteria—a microcosm of taxonomic novelty and phototrophic diversity

**DOI:** 10.1093/ismeco/ycag041

**Published:** 2026-02-27

**Authors:** Pia Marter, Henner Brinkmann, Heike M Freese, Victoria Ringel, Boyke Bunk, Michael Jarek, Michal Koblížek, Irene Wagner-Döbler, Jörn Petersen

**Affiliations:** Leibniz Institute DSMZ – German Collection of Microorganisms and Cell Cultures, Inhoffenstraße 7b, D-38124 Braunschweig, Germany; Julius Kühn Institute (JKI) – Federal Research Centre for Cultivated Plants, Erwin-Baur-Straße 27, D-06484 Quedlinburg, Germany; Leibniz Institute DSMZ – German Collection of Microorganisms and Cell Cultures, Inhoffenstraße 7b, D-38124 Braunschweig, Germany; Leibniz Institute DSMZ – German Collection of Microorganisms and Cell Cultures, Inhoffenstraße 7b, D-38124 Braunschweig, Germany; Leibniz Institute DSMZ – German Collection of Microorganisms and Cell Cultures, Inhoffenstraße 7b, D-38124 Braunschweig, Germany; Leibniz Institute DSMZ – German Collection of Microorganisms and Cell Cultures, Inhoffenstraße 7b, D-38124 Braunschweig, Germany; Helmholtz Centre for Infection Research, Inhoffenstraße 7b, D-38124 Braunschweig, Germany; Laboratory of Anoxygenic Phototrophs, Institute of Microbiology of the Czech Academy of Sciences, Novohradská 237, Opatovický mlýn, 379 01 Třeboň, Czech Republic; Helmholtz Centre for Infection Research, Inhoffenstraße 7b, D-38124 Braunschweig, Germany; Institute of Microbiology, Technical University of Braunschweig, Spielmannstraße 7, D-38106 Braunschweig, Germany; Leibniz Institute DSMZ – German Collection of Microorganisms and Cell Cultures, Inhoffenstraße 7b, D-38124 Braunschweig, Germany; Institute of Microbiology, Technical University of Braunschweig, Spielmannstraße 7, D-38106 Braunschweig, Germany

**Keywords:** cyanosphere, metagenome sequencing, phylogenomics, phototrophic deltaproteobacteria, proteorhodopsin, dual phototrophy

## Abstract

Intertidal biological mats are highly dynamic ecosystems typically dominated by filamentous cyanobacteria of the genus *Coleofasciculus*. These primary producers play important roles in primary production, biogeochemical cycling, and coastal protection. 16S rRNA gene profiling of non-axenic cultures has recently revealed an astonishing wealth of associated bacteria. We analyzed the microbiomes of 14 non-axenic *Coleofasciculus* cultures from nine globally distributed marine sampling sites, representing seven distinct phylogenomic lineages. Metagenome sequencing and binning resulted in 320 metagenome-assembled genomes (MAGs) representing a broad spectrum of “uncultivated” bacterial diversity mostly belonging to *Pseudomonadota*, *Bacteroidota* and *Planctomycetota*. *Marinovum algicola*, and *Roseitalea porphyridii* were found in 12 of the microbiomes studied, making them the most common housemates. The complex microbiome of *Coleofasciculus* sp. WW12 contained seven *Planctomycetota* MAGs from so far undescribed species, representing *inter alia* a new family in the order *Phycisphaerales* and an MAG from a deeply branching sister lineage of all cultivated planctomycetes. The discovery of 36 proteobacterial MAGs with photosynthesis gene clusters (PGCs) and 32 MAGs with proteorhodopsin or xanthorhodopsin operons documented the coexistence with many photoheterotrophic bacteria, indicating that the cyanosphere is a hotspot of phototrophic life. The presence of a PGC-containing *Myxococcales* MAG (*Candidatus* Photomyxococcus marinus) is of special interest because it paves the way to investigate photosynthesis in *Deltaproteobacteria*. In a Mediterranean *Coleofasciculus* culture, three alphaproteobacterial MAGs were found that have both a xanthorhodopsin operon and the PGC, suggesting that dual phototrophy is not restricted to alpine lakes or glaciers, and can also be found in marine habitats.

## Introduction

### Microbial diversity and the dilemma of environmental metagenomics

Metagenome sequencing combined with phylogenomic analyses provided insights into the astonishing diversity of uncultivated prokaryotic phyla and resulted in the current view of the tree of life [1, 2]. A comprehensive phylogenetic census of the Earth’s microbiomes in 2021 resulted in a catalog of >50 000 metagenome-assembled genomes (MAGs), comprising 12 556 putative candidate species from 135 different phyla [3]. Although the analysis of MAGs has enabled various new taxonomic, metabolic, and ecological inferences, cultivated microbes and model organisms remain indispensable for microbiology. However, the isolation rate of new microorganisms is heavily biased toward easily cultivatable bacteria. In 2024, >90% of the 1286 validly published new species names belonged to four prominent phyla (597 *Pseudomonadota*, 235 *Actinomycetota*, 188 *Bacillota*, 161 *Bacteroidota*), while not a single bacterium of a new phylum was described (see List of Prokaryotic names with Standing in Nomenclature [LPSN] statistics; [4]). The detection of MAGs from yet uncultivated lineages offers the promising perspective of a genome-guided isolation of the corresponding bacteria [5], but it also illustrates the greatest dilemma of environmental metagenomics, namely, the lack of access to the microorganisms of interest. In the future, the sampling dilemma could be overcome by a culturomics-based metagenomics approach [6, 7].

### The marine cyanobacterial genus *Coleofasciculus*

Filamentous cyanobacteria of the genus *Coleofasciculus* are the dominant primary producers in marine microbial mats [8], complex assemblages of vertically stratified microbial communities in intertidal regions that play an important role in surface stabilization and coast protection [9]. A brief overview of the relevance, global distribution, and annual dynamics of *Coleofasciculus* was provided in our recent community profiling study, mainly based on amplicon sequencing of the 16S rRNA gene [10]. Phylogenetic analyses are required to distinguish the morphologically very similar cyanobacteria of the genera *Coleofasciculus* and *Microcoleus* [8], which are located on two distinct branches of the cyanobacterial tree of life, namely, clade B3 and clade A [11, 12]. Remarkably, the characteristic bundle formation of both lineages is induced by specific heterotrophic bacteria and disappears in axenic cultures [8, 13]. *Coleofasciculus chthonoplastes* is the type species of the family *Coleofasciculaceae,* comprising filamentous, nonbranching, desiccation-resistant cyanobacteria without heterocysts, necridia, and akinetes [14]. A recent metagenome study illustrated the key function of *C. chthonoplastes* for the benthic microbial mat communities of the Solar Lake in Taba, Egypt [15]. The largest collection of *Coleofasciculus* strains sampled worldwide is deposited in the German Collection of Microorganisms and Cell Cultures (DSMZ).

### The cyanobacterial microbiome

The natural environment of cyanobacteria is not sterile, and isolated strains are typically accompanied by a considerable number of closely associated bacteria [12, 16]. Cultivation of a cyanobacterium in a test tube under optimal conditions mimics a continuous cyanobacterial bloom in the environment. Accordingly, the cyanosphere is a market of metabolites that mediate different types of bacterial interactions [17], which are only partially understood. Community analyses of *Microcystis aeruginosa* blooms and cocultivation experiments of the axenic cyanobacterium with phosphonate-degrading heterotrophs revealed their relevance in phosphate-deficient waters [18]. In contrast to the marine diazotroph *Trichodesmium erythraeum* IMS101, which is uniquely adapted for scavenging phosphorus from organic sources by a gene cluster for phosphonate import and hydrolysis (C-P lyase; [19]), *Microcystis* benefits from the corresponding metabolic capacity in the cyanosphere. *Trichodesmium* itself probably relies on associated siderophore-producing heterotrophs to overcome the chronic iron deficiency in the ocean [20]. The filamentous, non-nitrogen-fixing cyanobacterium *Microcoleus vaginatus* is the dominant primary producer in biocrust communities of dryland soils and also the main source of leaked organic carbon [21]. Heterotrophic bacteria with nitrogenase *nifH* genes are very abundant in the cyanosphere of *Microcoleus*, and coculture of the axenic cyanobacterium with diazotrophic heterotrophs confirmed a metabolic C for N exchange mediated by universal infochemicals [13, 22]. *Synechococcus*–Roseobacter cocultivation experiments exemplified the crucial role of nutrient recycling for the long-term stability of phototroph–heterotroph interactions [23]. It is therefore evident that cyanobacteria use light energy very efficiently to produce large amounts of carbon sources, which are released into the environment and thus serve as trade goods in exchange for growth-limiting nutrients.

Due to the historical peculiarity that cyanobacteria were for a long time studied by botanists [24, 25], their laborious axenization is not mandatory for the description of new species. Accordingly, most cyanobacterial isolates deposited in public culture collections such as the DSMZ, BCCM/ULC, CCAP, or SAG have not been purified and comprise an uncharacterized set of associated microbes, e.g. referred to as “*bacterial or other types of contamination* [sic].” Metagenome sequencing paved the way for state-of-the-art molecular and phylogenomic characterization of non-axenic cyanobacteria [12, 16, 26–28]. Recent studies have shown that the rapid establishment of stable cyanobacterial microbiomes after inoculation of axenic cyanobacteria with environmental water samples [29] demonstrated a comparable bacterial composition after years of continuous cultivation [10] and suggested the maintenance of defined communities over decades [12]. Based on the detection of >40 MAGs in single cultures of non-axenic cyanobacteria, we propose that these microbiomes of medium complexity are ideal resources for a metagenome-guided discovery of promising new bacteria, as shown in the current study.

### Aerobic anoxygenic phototrophs and rhodopsin-containing bacteria

The ability to harvest light energy is not restricted to cyanobacteria, and different phototrophic organisms are also present in the bacterial phylum *Pseudomonadota*. So-called purple bacteria harvest light energy using bacteriochlorophyll-containing photosynthetic complexes under anoxic or microaerophilic conditions [30]. However, there also exist a number of phototrophic *Pseudomonadota* that thrive under oxic conditions. These “aerobic anoxygenic phototrophic” (AAP) bacteria also use bacteriochlorophyll-containing photosynthesis complexes to generate metabolic energy but do not fix carbon [31]. Their apparatus is encoded by a photosynthesis gene cluster (PGC) with a size of ~45 kb. Despite the most plausible assumption that photosynthesis genes are usually inherited vertically, there is evidence for horizontal transfer of the entire PGC. The scattered distribution of photosynthesis in *Rhodobacterales* is best explained by horizontal operon transfers (HOTs; [32]). Moreover, a complete PGC transfer across phylum borders gave rise to phototrophy in the phylum *Gemmatimonadota* [33]. The recent discovery of PGCs in different myxococcal MAGs provided compelling evidence for the horizontal acquisition of photosynthesis by *Deltaproteobacteria* [34].

A completely different type of phototrophy employs microbial proton-pumping rhodopsins that were first discovered in halophilic *Archaea* [35]. Later, analogous proteorhodopsins were found in marine *Bacteria* in 2000 [36]. Upon illumination, these rhodopsins translocate protons across the cell membrane and the established gradient is used for adenosine triphosphate (ATP) synthesis. Homologous proteins with sensory or ion-pumping functions are widespread among bacteria, archaea, and even eukaryotes [37, 38]. Recently, it has been reported that *Sphingomonas glacialis* AAP5, cultured from the cold alpine lake Gossenköllesee, can perform dual phototrophy, i.e. it can harvest light using both bacteriochlorophyll and proton-pumping xanthorhodopsin [39]. It was suggested that *S. glacialis* uses the two different systems as a special adaptation to the alpine environment, which is characterized by strong fluctuations of solar irradiance and low temperatures.

### Aim of the study

The aim of the current metagenome study was to uncover the hidden diversity of heterotrophic marine bacteria stably associated with non-axenic cyanobacteria of the genus *Coleofasciculus* and to illustrate their scientific relevance. Our recent survey of amplicon sequence variants (ASVs) of the 16S rRNA gene suggested that between 2 and 75 different bacterial species are present in the cyanosphere of 32 investigated non-axenic strains [10], which likely reflects their individual history of isolation and purification. In our current study, we sequenced the metagenomes of 14 *Coleofasciculus* cultures from nine different oceanic regions worldwide, resulting in a total of 320 MAGs. Phylogenomic comparisons with the closest type strains allowed us to unveil the astonishing taxonomic novelty in the *Coleofasciculus* microbiome. Given the diversity of phototrophic bacteria detected in microbial mats [40, 41], we systematically studied the distribution of PGCs and proteorhodopsin and discovered a microcosm of phototrophic diversity.

## Materials and methods

### Cyanobacterial cultures

Fourteen non-axenic *Coleofasciculus* strains were obtained from the DSMZ (German Collection of Microorganisms and Cell Cultures, Braunschweig, Germany): SPW (DSM 104237), GNL1 (DSM 104238), SOL (DSM 104241), GNP5 (DSM 104239), CHI (DSM 104232), EBD (DSM 104233), BRE (DSM 104253), TOW (DSM 104236), STO (DSM 104242), SA18 (DSM 104244), SAH (DSM 104254), WW12 (DSM 104231), EDA (DSM 104234), WIS (DSM 101416; for strain history, see [10]). The cyanobacteria were grown in the recommended marine media (ASN3+: DSMZ medium 1673, MCL: DSMZ medium 1680, SWES: DSMZ medium 1831; [42]) at 17°C under low light conditions (3–4 μmol photons s^−1^ m^−2^) with a combination of three fluorescent tubes (Osram L30W/830 Lumilux warm white, Osram L30W/840 Lumilux cool white, Osram L30W/77 Fluora) at a day–night cycle of 16 h/8 h.

### Metagenome sequencing and binning

DNA from 14 non-axenic cyanobacteria was extracted with the DNeasy® Blood and Tissue Kit (Qiagen, Hilden, Germany) as previously described [12]. Illumina libraries were prepared from DNA isolated in 2019 [10] using the NEBNext Ultra II FS DNA Library Prep Kit (New England Biolabs, Frankfurt, Germany). Paired-end sequencing of the libraries (PE 150) was performed on the Illumina NovaSeq 6000 system using the v3 chemistry (600 cycles). Four libraries (SPW, CHI, EBD, WW12) were sequenced with 200 million reads per sample. Since this resulted in 1600-fold genome coverage of the cyanobacterial MAG in *Coleofasciculus* sp. CHI [10], we reduced the sequencing depth for the other 10 libraries to 50 million reads per library. Quality control and adapter clipping of the sequences were done using the fastq-mcf tool of ea-utils v1.04.803.

Sequence reads were assembled with MEGAHIT v1.2.7 [43] and the metagenomic assemblies were individually binned with MaxBin 2.0 v2.2.6 [44], MetaBAT v2.12.1 [45], and Concoct v1.1.0 [46]. The final set of MAGs was obtained by dereplication, aggregation, and scoring of the binning results with DAS Tool v1.1.2 [47]. Mapping of the raw reads on these MAGs was used to determine the genome coverage. The quality assessment of MAGs in terms of completeness and contamination was conducted with CheckM v1.0.13 [48]. For practical reasons, further analyses were mainly conducted with medium to high-quality MAGs with a calculated completeness >80% and a contamination rate <10% [49].

### Classification of metagenome-assembled genomes and comparison of microbiomes

The initial classification of MAGs was conducted with the Genome Taxonomy Database Toolkit GTDB-Tk v2.1.0 on the GTDB reference data version r207 [50, 51] and the type (strain) genome server TYGS [52]. A final taxonomic assignment was based on the LPSN [53]. The relationship of closely related MAGs was analysed by digital DNA–DNA hybridization (dDDH) using the Genome-to-Genome Distance Calculator 3.0 (GGDC; [4]). To compare the microbiome composition of the 14 non-axenic *Coleofasciculus* cultures and identify the influence of the medium and the sampling site, presence–absence matrices at different taxonomic levels (species, genus, family, order, class, phylum) were generated. For each taxonomic level, visualization of the data was conducted by *t*-distributed stochastic neighbour embedding (t-SNE; [54]) using the R package Rtsne (https://github.com/jkrijthe/Rtsne) with the default parameters but setting PCA to FALSE and perplexity to the highest acceptable perplexity.

### Abundance and commonality of metagenome-assembled genomes

In the current study, we distinguish between the “most abundant” and the “most common” bacteria in the cyanosphere and define them as follows: (i) The relative abundance of MAGs in the cyanosphere was calculated by mapping the Illumina reads on the MAGs and dividing the number of sequenced bases by the genome size [12]. Accordingly, the ‘abundance’ of a bacterium is a relative metric value determined individually for each microbiome on the basis of the genome coverage in comparison to the cyanobacterial host. Calibration to the *Coleofasciculus* MAG (=100%) allowed to compare the abundance of heterotrophic bacteria between different microbiomes. (ii) In contrast, the ‘commonality’ simply describes the presence of a bacterium among the total number of microbiomes investigated (reference: 14× metagenomes [Supplementary Table S1], 16S rRNA gene and internally transcribed spacer [16S-ITS] amplicons [10]).

### Characterization of functional genes

PGCs were identified by TBLASTN searches with the photosynthesis reaction center protein PufM of *Dinoroseobacter shibae* DFL 12 (WP_012180180.1). The PGC of *D. shibae* also served as a reference for the characterization of newly discovered PGCs from proteobacterial MAGs [32]. Proteorhodopsin genes and their corresponding operons were identified by TBLASTN searches with reference proteins of the three phylogenetic subtrees (Q9AFF7, WP_012370306, QBM75193).

### Phylogenetic analyses

Concatenated amino acid alignments of 92 housekeeping genes representing the bacterial core gene set were extracted with the up-to-date bacterial core gene set (UBCG) [55] and used to reconstruct genome-based phylogenies. The MUST package [56] and G-blocks [57] were subsequently used to manually refine the alignments by eliminating positions with gaps and highly divergent regions. Phylogenomic Maximum Likelihood trees were calculated with IQ-Tree under the LG + C40 + F + 4G model [58], including 1000 ultrafast bootstrap approximations [59]. Rhodopsin proteins and concatenated proteins of the photosynthesis reaction center (PufLM) were aligned with Clustal Omega [60] and evolutionary trees for rhodopsin and PufLM proteins were calculated by Maximum Likelihood analyses with MEGA7 v7.0.25 [61]. Phylogenetic trees with associated metadata were either visualized with the R package ggtree [62] or illustrated in PowerPoint.

## Results and discussion

### Metagenomic assessment of the cyanosphere

#### Genome sequencing and phylogenomic characterization of *Coleofasciculus*

**Figure 1:**
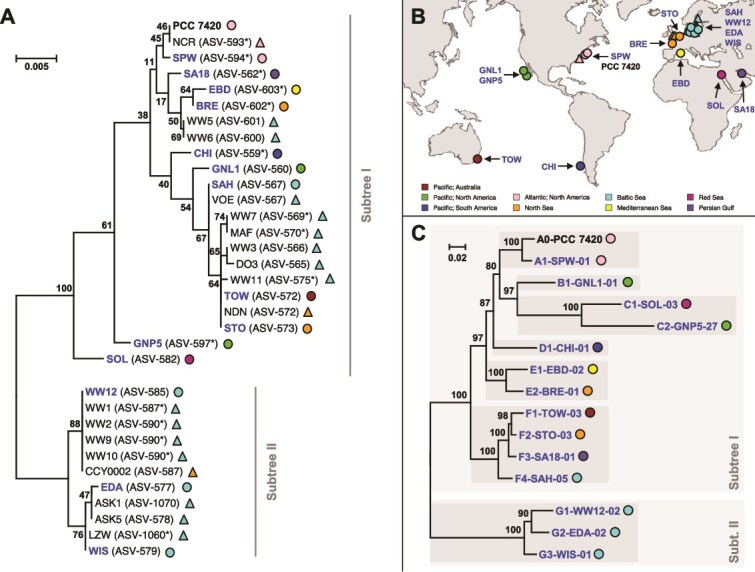
Phylogenetic relationship and biogeographic origin of 33 *Coleofasciculus* strains. (A) Phylogenetic 16S rRNA gene tree based on an alignment with 1461 nucleotide positions. Bootstrap proportions [BP] ≥ 30% are shown. Metagenome-sequenced strains are shown in blue. 16S- and metagenome-sequenced strains are indicated by triangles and circles, respectively. (B) Biogeography of the *Coleofasciculus* strains sampled from the intertidal zone of nine marine sampling sites (Pacific—Australia, Pacific—North America, Pacific—South America, Atlantic—North America, North Sea, Baltic Sea, Mediterranean Sea, Red Sea, Persian Gulf). (C) Phylogenomic tree of 15 *Coleofasciculus* strains. The RaxML tree was constructed based on 45 422 variable nucleotide positions from the underlying amino acid alignment of 92 housekeeping genes under the GTRIF4Γ model. The tree was midpoint rooted between Subtrees I and II. *Coleofasciculus* genomes are located in distinct lineages representing seven putative species (A–G; dark-gray boxes). *Coleofasciculus* genomes of the current study are labeled with unique identifiers comprising (i) a prefix with the phylogenomic lineage, (ii) the strain designation, and (iii) a numerical suffix reflecting the abundance of the respective MAG within the metagenome (Supplementary Table S1). *Most abundant ASV in cultures with allelic ribosomal operon variants.

Fourteen *Coleofasciculus* strains were selected for metagenome sequencing based on their phylogenetic position in the 16S rRNA gene tree (Fig. 1A) and a broad biogeographic sampling (Fig. 1B). Binning resulted in three to 40 MAGs (completeness >80%, contamination <10%) per culture (median = 21), and each metagenome contained a single cyanobacterial MAG of the genus *Coleofasciculus* (Supplementary Table S1). Based on the genome coverage, the cyanobacterium was the most abundant MAG in six metagenomes and among the top five in all but one sample.

The phylogenomic tree of the cyanobacterial MAGs confirmed the deep dichotomy of the genus *Coleofasciculus* (Fig. 1C), which was previously proposed by phylogenies of the 16S rRNA gene [8, 10]. In addition, seven distinct phylogenetic lineages with pairwise dDDH values clearly below 70% were identified (A–G, Fig. 1C; Supplementary Table S2A), suggesting that the corresponding strains represent different species of this mat-forming cyanobacterium.

#### Taxonomic and phylogenomic evaluation of bacteria from the cyanosphere

**Figure 2:**
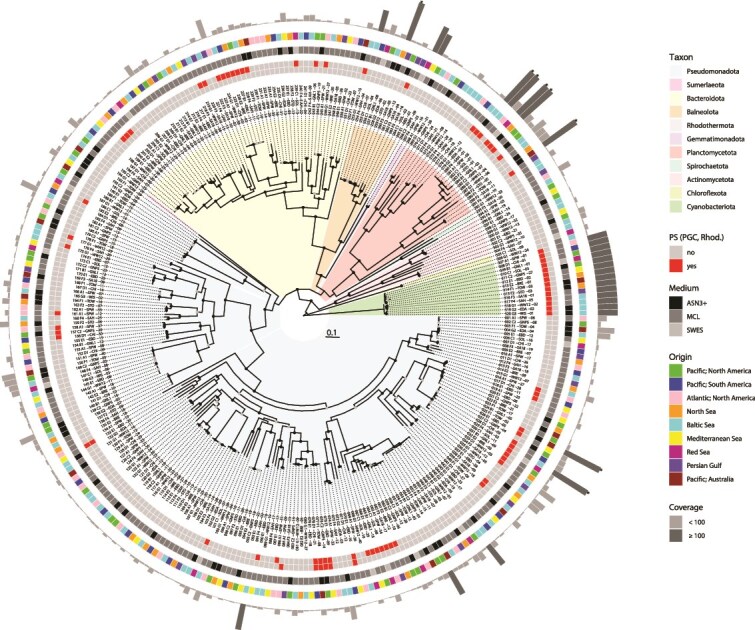
Phylogenomic tree of 320 MAGs from the cyanosphere of 14 *Coleofasciculus* strains. Taxonomic groups are differentiated at the phylum level; the complete tree and exact classification of MAGs and genome characteristics are presented in Supplementary Fig. S1 and Supplementary Table S1, respectively. The four rings from inside to outside show (i) the presence of bacteriochlorophyll-dependent photosynthesis in *Cyanobacteriota* and *Pseudomonadota* [photosynthesis gene cluster (PGC)], (ii) the presence of rhodopsin-dependent photosynthesis, (iii) the cultivation media of the *Coleofasciculus* strains, and (iv) the biogeographic origin of the cultures. The outermost gray columns represent the individual abundances of the MAGs in comparison to the cyanobacterial host (*Coleofasciculus* = 100%). Coverages of >100% are highlighted in dark gray. *Coverage values >250% were truncated. Absolute coverage values and an adjustment for strain GNP5 are shown in Supplementary Table S1.

In total, we obtained 320 MAGs (Supplementary Table S1) representing ten different phyla (Fig. 2, Supplementary Fig. S1F). More than 60% of the MAGs belong to *Pseudomonadota* (synonym: *Proteobacteria*; 193 MAGs), with the largest proportion of 135 *Alphaproteobacteria*, followed by 45 *Gammaproteobacteria*, 12 *Deltaproteobacteria*, and 1 betaproteobacterium. The wealth of *Alpha*- and *Gammaproteobacteria* is characteristic for the marine habitat, while the dominance of *Alpha*- and *Betaproteobacteria* in non-axenic cultures of freshwater cyanobacteria reflects limnic systems [12, 63]. Eighteen percent of the genomes were classified as *Bacteroidota* (59 MAGs), 8% as *Planctomycetota* (24 MAGs), and all other phyla accounted for <5%.

Based on digital DNA–DNA hybridization (dDDH), only one-sixth of the identified MAGs could be unambiguously assigned to a known species (52/320; Supplementary Fig. S1A, Supplementary Table S1). Our comparative phylogenomic analysis of the 320 MAGs indicated the presence of 68 new species, 28 new genera, 16 new families, 9 new orders, and 1 MAG from a bacterium of the widespread candidate phylum Sumerlaeota [64]. The most diverse microbiome of the genus *Coleofasciculus* was found in the WW12 culture (DSM 104231) [10], and 36 of the 40 MAGs in this culture probably represent new species (Supplementary Table S1).

#### Biogeographic distribution of *Coleofasciculus* and associated bacteria

The 33 examined *Coleofasciculus* strains from public culture collections [10] originated from nine coastal sites on four continents (Fig. 1B). By comparing the phylogenetic clustering of the strains with their biogeographic origin, a notable observation was made for the worldwide distribution of this genus. All but one of the strains of subtree II have been isolated from the Baltic Sea (Fig. 1A); the sole exception was strain CCY0002 originating from the Dutch North Sea island Schiermonnikoog. This distribution could be due to the high proportion of isolates from the Baltic Sea compared to other regions of the world, but it could also reflect specific adaptations of the phylogenetically distinct *Coleofasciculus* strains of subtree II to the brackish environment.

We then investigated whether the taxonomic composition of the associated bacteria reflects the biogeographic origin of their cyanobacterial hosts (Fig. 1B). The general observation from well-sampled phylogenetic lineages such as *Alphaproteobacteria* is a comparably random occurrence of MAGs from all regions worldwide without striking differences, as illustrated by the second outermost ring in Fig. 2. Furthermore, we compared the species distribution between the 14 *Coleofasciculus* metagenomes using a t-SNE analysis (Supplementary Fig. S2, Supplementary Table S3) to investigate the relevance of the original sampling site for the composition of the microbiome. However, the 2D mapping revealed no clustering at the species level, and a comparably random distribution was also observed at genus, family, class, and phylum levels (Supplementary Fig. S2). The only exception was the microbiome comparison at the order level, but clustering did not correlate with the biogeographical origin and was rather influenced by the cultivation medium of the cyanobacterium (ASN3+, MCL, SWES). Accordingly, our study provided clear evidence that the current composition of *Coleofasciculus* microbiomes in our culture collection is not determined by the biogeography of the cyanobacterial host.

### The marine microbiome of *Coleofasciculus*

#### The most common housemates

**Figure 3:**
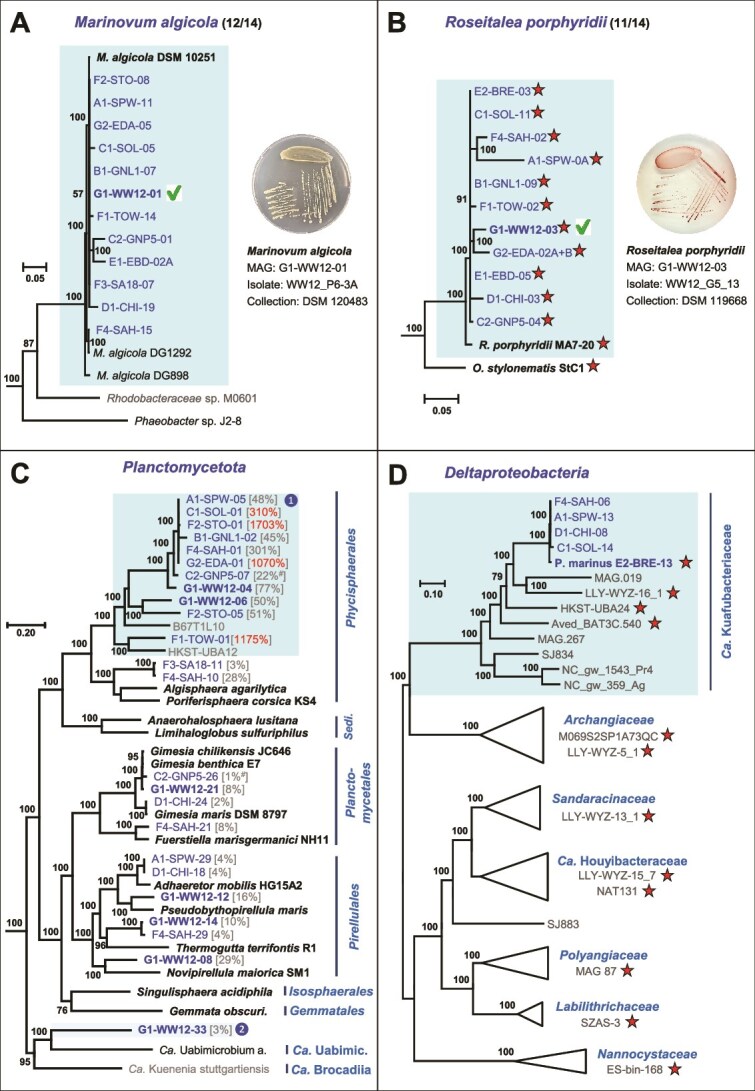
Phylogenomic analyses of *Coleofasciculus* MAGs and their closest related type strains. Complete phylogenies are shown as supplemental figures: (A) *Marinovum algicola*, Supplementary Fig. S3; (B) *Roseitalea porphyridii*, Supplementary Fig. S4; (C) *Planctomycetota*, Supplementary Fig. S5. The genome coverage in comparison to *Coleofasciculus* (100%) is shown in brackets; values >100% are highlighted in red. #, coverage ratios of strain GNP5 were adjusted (see Supplementary Table S1). Boxes with “**
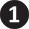
**” and “**
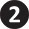
**” represent a new family and order, respectively; (D) *Deltaproteobacteria*, Supplementary Fig. S7. Accession numbers and metadata are provided in Supplementary Table S7. *Coleofasciculus* and reference MAGs are shown in blue and gray, respectively. Type strains, MAGs of *Coleofasciculus* sp. WW12 and reference MAGs of particular interest are highlighted in bold. Red stars indicate the presence of photosynthesis gene clusters (PGCs). Green hooks reflect the successful isolation of the corresponding bacterium. *O. stylonematis*, *Oceaniradius stylonematis* StC1; *Sedi*., *Sedimentisphaerales*; *Ca*. Uabimic., *Candidatus* Uabimicrobiales; *P. marinus*, *Candidatus* Photomyxococcus marinus; *Ca*., *Candidatus*.

Our recent 16S-ITS study detected a set of seven heterotrophic bacteria in at least 20 of 32 *Coleofasciculus* cultures investigated [10]. Corresponding MAGs of all seven taxa, namely, *Marinovum algicola*, *Roseitalea porphyridii*, *Algiphilus acroporae*, *Balneola* sp., *Nitratireductor* sp., *Imperialibacter* sp., and *Roseovarius* sp., were found in 5–12 of 14 metagenomes examined (Supplementary Table S4A). Surprisingly, nine MAGs of these common housemates lacked a corresponding amplicon sequencing variant (ASV) of the 16S rRNA gene (Supplementary Table S4B), which is likely related to primer binding and competition during PCR amplification [10]. Metagenome sequencing of *Coleofasciculus* sp. SPW and GNL, for example, revealed the presence of *M. algicola*, which confirms our previous recommendation to perform 16S-ITS amplicon sequencing and metagenomic binning in parallel [10]. In contrast, the absence of individual MAGs with corresponding ASVs is likely related to the sequencing depth [65], which did not allow for the recovery of metagenomes from bacteria that were more than two orders of magnitude less abundant than the cyanobacterium. The two most common MAGs, *M. algicola* (*Rhodobacterales*, *Alphaproteobacteria*) and *R. porphyridii* (*Hyphomicrobiales*, *Alphaproteobacteria*), were detected in 12 and 11 of 14 metagenomes, respectively, and are therefore expected to play a central role in the microbiome of *Coleofasciculus* (Fig. 3A and B; Supplementary Figs S3 and Supplementary S4; Supplementary Text S1). The corresponding bacteria have already been isolated from *Coleofasciculus* sp. WW12 and were deposited at the DSMZ culture collection (DSM 120483, DSM 119668).

#### The most abundant housemates

The genome coverage comparison of different MAGs in the metagenomes provided new insights into the ratio of “associated bacteria” versus “cyanobacterial host,” reflecting the abundance of individual bacteria of the cyanosphere. The by far most abundant heterotrophs of the cyanosphere are represented by MAGs of the *Planctomycetota* order *Phycisphaerales* (MAGs: 279–291; Fig. 2, Supplementary Fig. S5) showing planctomycete:phototroph ratios of 17:1 (F2-STO-01), 12:1 (F1-TOW-01), and 11:1 (G2-EDA-01). The uppermost six closely related MAGs in the *Planctomycetota* tree (A1-SPW-05 to G2-EDA-01, Fig. 3C), which belong to the same species (dDDH >99.5%; Supplementary Table S2I), showed ratios between 0.45:1 and 17:1. Accordingly, the abundance of a given bacterium in different *Coleofasciulus* microbiomes varies by a factor of up to 40, likely depending on individual growth conditions, metabolite exchange, and the community composition. The phylogenomic *Planctomycetota* tree illustrates the hidden microbial diversity in the cyanosphere (Fig. 3C). The metagenomic analysis of 14 *Coleofasciculus* cultures revealed the presence of 24 planctomycete MAGs, and 7 of them were found in the metagenome of *Coleofasciculus* sp. WW12 (Supplementary Table S1). TYGS analyses with the closest related-type strains showed that all planctomycete MAGs of WW12 represent new species, and the GTDB-Tk analysis provided further insights into their taxonomic novelty. G1-WW12-08 and G1-WW12-14/F4-SAH-29 are two examples of new genera within the families *Pirellulaceae* and *Thermoguttaceae* (Supplementary Fig. S5), respectively. Furthermore, our phylogenomic analysis confirmed the GTDB-Tk results and showed that the highlighted subtree ranging from A1-SPW-05 to HKST-UBA12 represents the genomic diversity of a yet uncultivated family within the order *Phycisphaerales* (Fig. 3C). The wealth of 13 *Phycisphaerales* MAGs in the current study is remarkable, as the class *Phycisphaerae* was barely studied in the past [66]. The MAGs from the cyanosphere belong to four uncharacterized genera: (i) A1-SPW-05 to G1-WW12-04, (ii) G1-WW12-06/F2-STO-05, (iii) F1-TOW-01, and (iv) F3-SA18-11/F4-SAH-10. Finally, the discovery of G1-WW12-33, an MAG of the *Coleofasciculus* sp. WW12 microbiome with a very low abundance (planctomycete:phototroph ratio 0.03:1; Supplementary Table S1), is noteworthy. It represents an uncultivated strain of at least a new order or even a separate class within a deeply branching sister lineage of all cultivated planctomycetes (Fig. 3C, Supplementary Fig. S5), which also comprises the “phagotrophic” bacterium *Candidatus* Uabimicrobium amorphum SRT547 and the anaerobic ammonium oxidizing (anammox) bacterium *Candidatus* Kuenenia stuttgartiensis CSTR1 [67, 68]. Our comparative metagenome analyses demonstrate that the microbiome of *Coleofasciculus* is a treasure trove for future cultivation attempts of new planctomycete lineages that have evaded detailed investigation due to lack of isolates.

### The diversity of phototrophic bacteria in the cyanosphere

#### Different phototrophic lifestyles

Beyond the cyanobacterial oxygenic photosynthesis with photosystems I and II [69], two additional modes of bacterial phototrophy were found in the current study [31, 36]. First, we detected PGCs in 36 of 306 MAGs of associated bacteria (Fig. 2, Supplementary Fig. S6), which documented that the cyanosphere of *Coleofasciculus* usually comprises between two and three proteobacteria with PGCs. Second, rhodopsin genes were detected in 46 MAGs of *Alphaproteobacteria*, *Actinomycetota*, *Bacteroidota*, *Balneolaeota,* and *Planctomycetota* (Supplementary Table S5). Phylogenetic analyses and the investigation of the genomic context were performed to draw valid conclusions about their role in bacterial photosynthesis (see below).

#### A deltaproteobacterial metagenome-assembled genomes with a photosynthesis gene cluster

**Figure 4:**
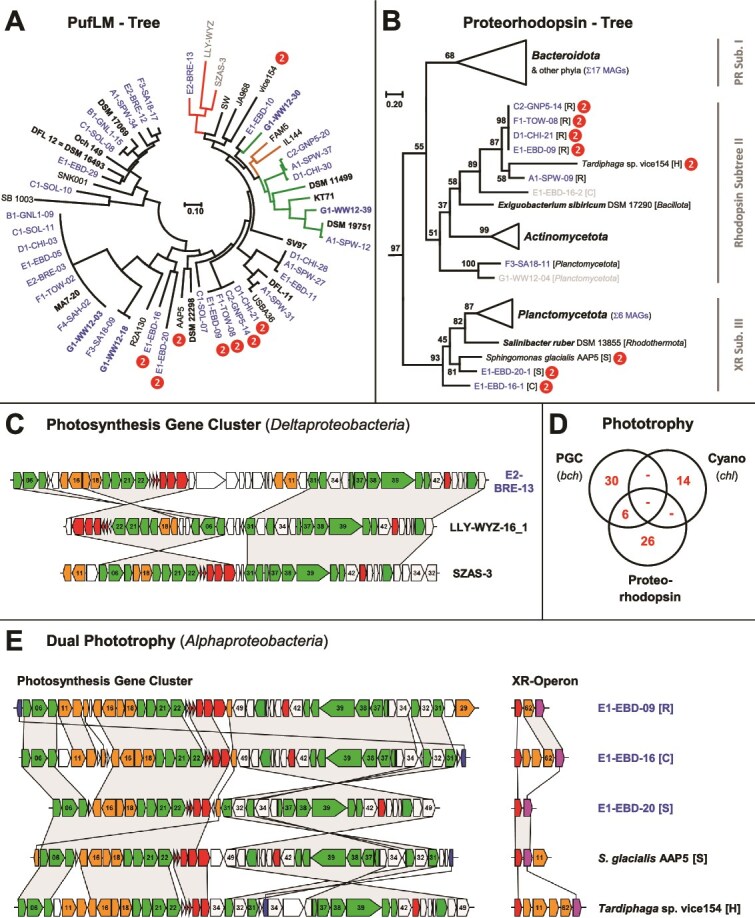
Distribution of anoxygenic photosynthesis in the cyanosphere. (A) PufLM tree of 58 concatenated PufL and PufM protein sequences of the photosynthesis gene cluster (PGC). MAGs of the current study are shown in blue, reference MAGs are shown in gray, and type strains are highlighted in bold. The color of the branches reflects different proteobacterial lineages: *Alphaproteobacteria*—black, *Betaproteobacteria*—orange, *Gammaproteobacteria*—green, and *Deltaproteobacteria*—red. “**
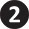
**” indicates the presence of proton-pumping rhodopsin (dual phototrophy). LLY-WYZ, strain LLY-WYZ-16_1. The complete phylogeny is shown in Supplementary Fig. S6. (B) Rhodopsin tree with three xanthorhodopsin (XR)/proteorhodopsin (PR) subtrees based on 73 protein sequences. “**
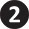
**” indicates the presence of a PGC (dual phototrophy). MAGs with *bona fide* light-driven proton pumps are highlighted in blue (Supplementary Table S5A). The complete phylogeny is shown in Supplementary Fig. S8. (C) Synteny plot of three deltaproteobacterial PGCs including *Ca.* Photomyxococcus marinus (E2-BRE-13). Color code of genes: green, bacteriochlorophyll biosynthesis (*bch*); orange, carotenoid biosynthesis (*crt*); red, light-harvesting and photosynthesis reaction center (*puf*); dark blue, cytochrome c_2_ (*cycA*, Fig. 4C); gray, additional conserved genes of the PGC; white, nonconserved genes. Genes were numbered according to the reference PGC of *D. shibae* DFL 12 [32]. Locus tags, localization, and classification of genes are presented in Supplementary Table S6. (D) Venn diagram of the distribution of photosynthesis in 320 MAGs of the current study. Cyano, oxygenic photosynthesis in cyanobacteria with photosystem I and II. (E) Dual phototrophy in *Alphaproteobacteria* with synteny plots of three new MAGs from *Coleofasciculus* sp. EBD and two reference strains [78, 99]. Color code of xanthorhodopsin operon genes: red, xanthorhodopsin (XR); pink, -carotene 15,15´-dioxygenase (*brp*/*blh*). Details of the operon composition are presented in Supplementary Table S5A and Supplementary B. Alphaproteobacterial lineages are abbreviated as follows: *Rhodospirillales* [R], *Caulobacterales* [C], *Sphingomonadales* [S], and *Hyphomicrobiales* [H].

The phylogenetic tree of concatenated PufLM protein sequences from 36 newly established photosynthetic MAGs and 22 reference sequences reflects the scattered distribution of photosynthesis in *Pseudomonadota* (Fig. 4A). Twenty-nine PGCs were found in five orders of *Alphaproteobacteria* (13× *Hyphomicrobiales*, 7× *Rhodobacterales*, 7× *Rhodospirillales*, 1× *Sphingomonadales*, 1× *Caulobacterales*; Supplementary Fig. S6), and the localization of *Rhodospirillales pufLM* genes in three distant regions of the tree likely reflects frequent HOTs of the PGC [32]. Two gammaproteobacterial PGCs (G1-WW12-39, A1-SPW-12) were located in a well-supported *Cellvibrionales* subtree together with *Congregibacter litoralis* KT71 and *Pseudohaliea rubra* DSM 19751 (Supplementary Fig. S6), both capable of aerobic anoxygenic photosynthesis [70, 71]. With regard to phototrophy, the probably most remarkable finding of the current study was the discovery of a culture-associated deltaproteobacterial MAG with a complete PGC (E2-BRE-13; Fig. 4C, Supplementary Table S6), representing a bacterium of the order *Myxococcales* (*Deltaproteobacteria*), for which we propose the name *Candidatus* Photomyxococcus marinus (see below). The common branching of *Ca.* Photomyxococcus marinus (MAG: E2-BRE-13), together with two other deltaproteobacterial MAGs (LLY-WYZ-16_1, SZAS-3) in a distinct PufLM subtree (Fig. 4A) and the localization of its PGC on a large contig with a size of 416 kb (JAVKDZ010000082.1), supports the authenticity of our metagenome assembly and binning. The first indication of phototrophic *Deltaproteobacteria* (alternatively *Myxococcota* [72]) was reported in 2019 on the basis of two MAGs and published at ResearchGate (NAT131 [GCA_002699025.1], Ga0077550 [GCA_001464385.1]; [73]). A recent comprehensive metagenome study showed that six discrete lineages of *Deltaproteobacteria* contain PGCs, confirmed the expression of the photosynthetic genes by metatranscriptomics, and documented the functionality of deltaproteobacterial pigment biosynthesis genes by heterologous expression in *Rhodobacter sphaeroides* [34]. The comparison of PGCs from *Ca*. Photomyxococcus marinus (E2-BRE-13), LLY-WYZ-16_1, and SZAS-3 revealed the characteristic synteny with several gene rearrangements reflecting the individual evolution of photosynthesis in *Deltaproteobacteria* (Fig. 4C, [74]). We analyzed the phylogenomic position of 11 deltaproteobacterial MAGs from the current study together with phototrophic reference MAGs and genome-sequenced type strains to determine the closest relatives of *Ca*. Photomyxococcus marinus and to illustrate the distribution of photosynthesis in *Deltaproteobacteria* (Fig. 3D, Supplementary Fig. S7). *Ca.* Photomyxococcus marinus (E2-BRE-13) is located together with three other phototrophic MAGs (LLY-WYZ-16_1, HKST-UBA24, Aved_18-Q3-R54-62_BAT3C.540) and nine non-phototrophic MAGs in the candidate family Kuafubacteriaceae [34]. *Ca*. Photomyxococcus marinus forms a distinct branch with four very closely related but non-phototrophic MAGs (F4-SAH-06, A1-SPW-13, D1-CHI-08, C1-SOL-14), which reflects either a recent gain or a recent loss of phototrophy. The distribution of PGCs in Kuafubacteriaceae that were found in a wide range of habitats (Fig. 3D, Supplementary Table S7D) can be explained by a common photosynthetic ancestry and four losses (Scenario 1), four comparably recent HOTs (Scenario 2), or a mixed evolutionary scenario.

The phylogenomic tree illustrates the dispersed distribution of phototrotrophic MAGs in seven different lineages of *Deltaproteobacteria* (Fig. 3D, Supplementary Fig. S7), representing names of five families validly published under the ICNP (*Archangiaceae*, *Sandaracinaceae*, *Polyangiaceae*, *Labilitrichaceae*, *Nanocystaceae*) and two non-validly published family names, namely, *Ca.* Kuafubacteriaceae and *Ca.* Houyibacteraceae. The scattered localization of PGCs in the phylogenetic tree of *Deltaproteobacteria* is reminiscent of the PGC distribution in *Rhodobacterales*, where HOT was shown to be the main driver of its phylogenetic distribution [32], but for the *Deltaproteobacteria*, it is still an open question whether it reflects frequent horizontal exchanges via HOTs or a strictly vertical evolution with multiple losses [73]. The remarkable wealth of phototrophic MAGs from *Myxococcales* provides further insights into the ecological niches of these yet uncultivated *Deltaproteobacteria* (Supplementary Table S7D; [34]). They were detected in activated sludge from wastewater treatment plants (HKST-UBA24), found in drinking water (LLY-WYZ-5_1), on the surface of a glacier (ES-bin-168), and in the cyanosphere of *Microcystis* from a freshwater lake (M069S2SP1A73QC; [75]). The frequent detection of phototrophic *Myxococcales* MAGs in pelagic seawater samples from the open ocean (Pacific, Gulf of Mexico, North Atlantic), in the sediment of a wetland lagoon (LLY-WYZ-13_1, -14_1, -16_1; Baja California) and the cyanosphere of *Coleofasciculus*, reflected their abundance in marine habitats. The detection of phototrophic representatives in a broad spectrum of different ecosystems and in at least seven different families thus reflects the relevance of light energy for the biology and evolution of *Deltaproteobacteria*. Finally, the non-axenic culture *Coleofasciculus* sp. BRE from our collection offers a promising perspective to isolate a bacterium of this fascinating group of photoheterotrophs for the first time.

#### Distribution of rhodopsin-driven photosynthesis

The detection of 48 rhodopsin genes in 46 of 320 newly established MAGs indicates that light-activated proton pumping for ATP production plays a considerable energetic role in the cyanosphere of *Coleofasciculus* (Supplementary Table S5A). This proportion is in striking agreement with previous calculations from natural marine habitats, according to which proteorhodopsin-exploiting bacteria account for at least 15% of microorganisms in the photic zone [76]. However, in contrast to *pufLM* genes of the PGC that are diagnostic markers for anoxygenic photosynthesis [77], rhodopsins have a broad range of physiological functions, including proton pumping for ATP generation and survival under starvation, maintenance of homeostasis via chloride and sodium ion pumping, and light sensing for phototactic responses [37, 38]. Therefore, we investigated the phylogenetic placement of all 48 detected rhodopsin proteins in the context of established reference sequences [38, 78, 79]. Apart from one Na-pumping rhodopsin found in E1-EBD-20 and not further considered, all 47 identified sequences represent proton-pumping bacterial rhodopsins belonging to three different subtrees of proteo- and xanthorhodopsins (Fig. 4B, Supplementary Fig. S8) [38]. Furthermore, three amino acid positions that are diagnostic for both proteo- and xanthorhodopsin proteins (D-85, T-89, E-96, [80]) were almost universally conserved in the alignment (Supplementary Table S5B). A notable exception is the divergent position 96 in a separate branch of eight rhodopsin proteins in subtree II, ranging from C2-GNP5-14 to *Exiguobacterium sibiricum* DSM 17290 (Fig. 4B). However, the well-studied rhodopsin from *E. sibiricum* is a functional proton pump [81, 82], which shows that glutamic acid (E) at position 96 is not strictly necessary. A diagnostic lysine (K-292) from the polar transmembrane domain TM7 for the binding of retinal ligands [83] is present in all but three of the rhodopsins examined (Supplementary Table S5B). The functional role of the three closely related, fast-evolving rhodopsin-homologs (MEQ8652030.1, WP_137929642.1, WP_350157907.1; Supplementary Fig. S8), which also lack the characteristic DTE motif (Supplementary Table S5B), is unclear. It has recently been demonstrated that many proton-pumping rhodopsins contain an additional carotenoid antenna that expands their optical cross section [39, 84–86], and it has been proposed that the presence of a glycine at position 156 (G-156) facilitates its binding [84]. The diagnostic glycine was present in all proteorhodopsin sequences from *Bacteroidota* in subtree I and the xanthorhodopsins from subtree III (Fig. 4B), including the sequences from the potentially dual-phototrophs E1-EBD-20 and E1-EBD-16-1 (see below). This finding suggests that the corresponding marine bacteria likely use rhodopsins with carotenoid antennas to capture light more efficiently. Closer examination of rhodopsin operons revealed a typical structure with a *brp*/*blh* gene and optionally up to four carotenoid biosynthesis genes (Fig. 4E, Supplementary Table S6, Supplementary Table S5A). The bacteriorhodopsin-related protein (Brp)/bacteriorhodopsin-related protein-like homolog protein (Blh) encodes a -carotene 15,15′-dioxygenase for the release of all-*trans*-retinal [87], which is crucial for the functionality of proteorhodopsin. The rhodopsin operons from the current study comprise up to three additional carotenoid genes (Supplementary Table S5A), which are all required for retinal biosynthesis [88]. Based on their phylogenetic placement and the presence of a *brp*/*blh* gene within the respective metagenome, we conclude that 32 proteorhodopsins of the current study represent *bona fide* light-driven proton pumps (Supplementary Fig. S8, Supplementary Table S5A). Light can thus be used for flagellar motility, ATP production, or metabolite accumulation [89–91] and could even stimulate the growth of the respective marine bacteria in the cyanosphere [92].

#### Prediction of dual phototrophy in marine bacteria

The detection of 36 PGC- and 32 proteorhodopsin-containing MAGs in 14 examined *Coleofasciculus* cultures showed that photoheterotrophic bacteria are common in these marine consortia. A notable finding is the presence of six MAGs comprising both the PGC with bacteriochlorophyll genes and a xanthorhodopsin operon (Fig. 4D), suggesting that the respective bacteria can perform dual phototrophy. This remarkable physiological trait has previously been discovered in the freshwater bacterium *S. glacialis* AAP5, which was isolated from an alpine lake in Tyrol, Austria [39]. Four of the MAGs represent a single *Rhodospirillales* species (dDDH >99.5%; Supplementary Table S2G) sampled from intertidal habitats in the Mediterranean Sea (E1-EBD-09) and the North American, South American, and Australian Pacific (C2-GNP5-14, D1-CHI-21, F1-TOW-08; Fig. 2). They contain absolutely conserved PGCs and identical xanthorhodopsin operons, which include the xanthorhodopsin gene, a carotenoid biosynthesis gene, and the crucial β-carotene 15,15′-dioxygenase (*XR*, *crtY*, *brp*/*blh*; Supplementary Table S6). This finding documents the authenticity of the respective MAGs that originated from different microbial consortia and were independently assembled and binned. The most diverse cyanobacterial culture in terms of dual phototrophy is *Coleofasciculus* sp. EBD, which was sampled in 1993 from the intertidal zone of the Mediterranean Ebro Delta in Spain [93]. In addition to the *Rhodospirillales* MAG (E1-EBD-09), it contains two additional alphaproteobacterial MAGs from the orders *Caulobacterales* (E1-EBD-16) and *Sphingomonadales* (E1-EBD-20). Assessment of the photosynthetic gene inventory in the *Caulobacterales* MAG documented the presence of a genuine xanthorhodopsin operon (*XR*, *crtE*, *crtB*, *crtY*, *brp*/*blh*) and a solitary rhodopsin gene without an adjacent dioxygenase and carotenoid genes (Fig. 4E, Supplementary Table S5a). The comparison of the three dual phototrophs from the EBD culture revealed a different number of carotenoid genes in their xanthorhodopsin operons and structural rearrangements of their PGCs, which are characteristic for photosystems from different proteobacterial lineages [32, 70, 74]. Two *Sphingomonadales* with dual phototrophy, namely, the marine *Erythrobacter* sp. MAG (E1-EBD-20) and the alpine isolate *S. glaciales* AAP5 [39, 78], showed a close phylogenetic relationship of their xanthorhodopsins (Fig. 4B), whereas the PufLM proteins were not specifically related (Fig. 4A), as reflected by the rearrangements of their PGCs (Fig. 4E). Overall, the current study proposes the presence of dual phototrophy in three orders of marine *Alphaproteobacteria* (*Rhodospirillales*, *Caulobacterales*, *Sphingomonadales*). The use of two photosynthetic modes is clearly not limited to freshwater bacteria and harsh arctic or alpine habitats, but could also be important for phototrophs inhabiting temperate marine ecosystems.

### Conclusion and outlook

#### The potential of non-axenic cultures in the post-genomic era

Non-axenic cyanobacteria were formerly regarded as the “grubby urchins” of microbial culture collections, although the benefits of associated heterotrophs in vitamin provision, reciprocal metabolite exchange, and protection from reactive oxygen species have been acknowledged [23, 94–96]. The current metagenome study has shown that they are promising resources of hidden bacterial biodiversity. Non-axenic cyanobacteria represent ecological time capsules that preserve the associated microbial diversity of the phototrophic host for decades [12, 16, 95]. In addition, they display unique fingerprints of the bacterial flora at the sampling site, selected by the medium and cultivation conditions of the respective cyanobacterium [29]. Their metagenomic characterization allows the development of specific genome-guided isolation strategies for fastidious bacteria. In contrast to MAGs from environmental studies, the main advantages of non-axenic cultures are (i) the pre-existing enrichment of the bacterium of interest in a low-complexity community and (ii) the continuous access to the sample.

#### The cyanosphere as a reservoir of anoxygenic phototrophs

The cyanobacterial holobiont combines all three types of phototrophy that emerged on our planet during >3 billion years of evolution, utilizing light-harvesting systems based on chlorophyll, bacteriochlorophyll, and microbial rhodopsins [92]. The diversity of phototrophic bacteria in the cyanosphere of *Coleofasciculus* corresponds to the abundance of aerobic anoxygenic phototrophs in limnic *Microcystis* blooms [97] and indicates that non-axenic cyanobacteria are promising reservoirs for the discovery of new features of phototrophy. AAP species are often found in association with algae or cyanobacteria. The first AAP species, *Erythrobacter longus*, was isolated from the surface of marine seaweed [98]. Light and host exudates likely promote the observed enrichment of photoheterotrophs, and individual absorption spectra of the photosystems could avoid competition for “the place in the sun.” A notable finding was the presence of three MAGs with the genes for dual phototrophy in the metagenome of *Coleofasciculus* sp. EBD (Fig. 4). The detection of different light-harvesting systems in *Sphingomonadales* is in agreement with the outcome of a recent study [78], but their presence in *Rhodospirillales* and *Caulobacterales* indicates that strains with two modes of photosynthesis can be found in various orders of *Alphaproteobacteria*. In contrast to the previous reports of dual phototrophy in limnic bacteria from alpine and arctic habitats [78, 99], our study showed the presence of both photosystems in marine bacteria from the intertidal zone of the Mediterranean Sea. The benefit of two fundamentally different systems for light energy harvesting for *S. glacialis* AAP5, which was isolated from an alpine lake, has been correlated with extreme changes of light and low temperature [39]. Bacteria from intertidal zones also experience extreme fluctuations, particularly in terms of salinity, turbulence, light intensity, temperature, and nutrient availability. Therefore, dual phototrophy may allow them to fine-tune their light-harvesting apparatus according to the different light intensity or solar spectrum in the particular habitat. Accordingly, isolation and investigation of these mesophilic marine strains would greatly advance our understanding of dual phototrophy.

## Taxonomic proposal

### Description of *Ca.* Photomyxococcus marinus gen. nov., sp. nov.

(Pho.to.my.xo.coc’cus. Gr. neut. n. *phôs*, light; Gr. fem. n. *myxa*, mucus, slime; N.L. masc. n. *coccus*, coccus; from Gr. masc. n. *kokkos*, grain, seed; N.L. masc. n. *Photomyxococcus*, a photosynthetic slime coccus; ma.ri’nus. L. masc. adj. *marinus*, of the sea). This marine bacterium lives in the culture of the filamentous cyanobacterium *Coleofasciculus* sp. BRE (DSM 104253). It is represented by the MAG E2-BRE-13 (JAVKDZ000000000.1) with a size of 11.9 Mbp and a G + C content of 69.0%; the complete protologue can be found in Supplementary Table S8. It belongs to the family *Candidatus* Kuafubacteriaceae, order *Candidatus* Kuafubacteriales.

## Supplementary Material

Figure-S1_UBCG-Tree_320-MAGs_251024_ycag041

Figure-S2_Taxon-Matrix_tSNA_251024_ycag041

Figure-S3-S8_UBCG-Trees_260224_ycag041

Table-S1_Metagenomes_Coleofasciculus_251024_ycag041

Table-S2_ddDH_Matrices_251024_ycag041

Table-S3_Taxonomy-Matrix_t-SNE_251024_ycag041

Table-S4_Distribution_MAGs_and_ASVs_251024_ycag041

Table-S5_Coleo-MAG_Rhodopsin-Operons_260223_ycag041

Table-S6_Coleo-MAG_PGC-Genes_251024_ycag041

Table-S7_Phylogenies-Reference-Genomes_260223_ycag041

Table-S8_Taxonomy_Protologue_251024_ycag041

Text-S1_Coleofasciculus_260122_ycag041

## Data Availability

The datasets generated and analyzed during the current study are available in the NCBI repository. All MAGs and the corresponding filtered and trimmed raw sequence data have been deposited at the NCBI under the following BioProjects: *Coleofasciculus* sp. SPW (PRJNA993097), GNL1 (PRJNA993446), SOL (PRJNA993462), GNP5 (PRJNA993461), CHI (PRJNA993444), EBD (PRJNA993099), BRE (PRJNA993106), TOW (PRJNA993458), STO (PRJNA993456), SA18 (PRJNA993115), SAH (PRJNA993447), WW12 (PRJNA993463), EDA (PRJNA993471), and WIS (PRJNA993482).
