# Supplementary material for: The microbiome of marine mat-forming cyanobacteria—a microcosm of taxonomic novelty and phototrophic diversity
Source: ISME Commun. 2026 Feb 27;6(1):ycag041. doi: 10.1093/ismeco/ycag041 (PMC13043013; doi:10.1093/ismeco/ycag041)
Supplement: Figure-S1_UBCG-Tree_320-MAGs_251024_ycag041 [file figure-s1_ubcg-tree_320-mags_251024_ycag041.pptx]

## Slide 1
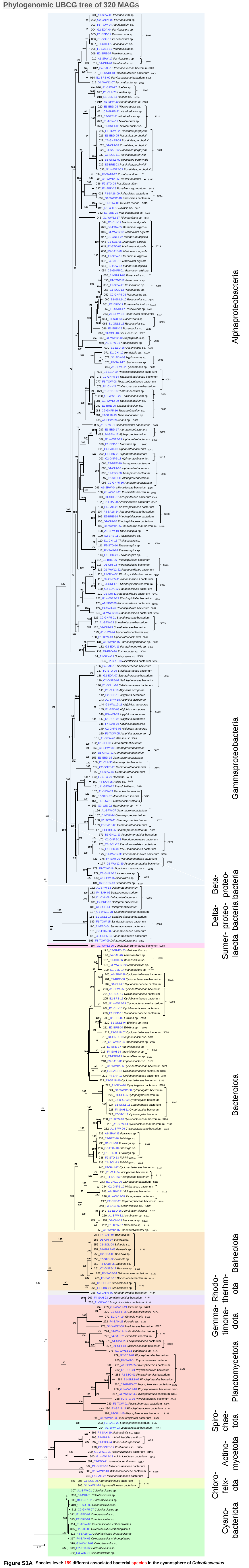

Phylogenomic UBCG tree of 320 MAGs
 001_A1-SPW-06 Parvibaculum sp.
 002_C2-GNP5-08 Parvibaculum sp.
97
 003_F1-TOW-04 Parvibaculum sp.
96
 004_G2-EDA-04 Parvibaculum sp.
S001
 005_E1-EBD-12 Parvibaculum sp.
100
 006_C1-SOL-16 Parvibaculum sp.
 007_D1-CHI-17 Parvibaculum sp.
 008_F3-SA18-19 Parvibaculum sp.
100
 009_E2-BRE-07 Parvibaculum sp.
 010_A1-SPW-17 Parvibaculum sp.
S002
100
100
 011_D1-CHI-26 Parvibaculum sp.
S003
 012_F4-SAH-16 Parvibaculaceae bacterium
98
100
S004
100
 013_F3-SA18-18 Parvibaculaceae bacterium
S005
 014_E2-BRE-09 Parvibaculaceae bacterium
S006
 015_G1-WW12-07 Pyruvatibacter sp.
016_A1-SPW-27 Hoeflea sp.
100
S007
100
 017_D1-CHI-28 Hoeflea sp.
S008
 018_E1-EBD-11 Hoeflea sp.
S009
019_ A1-SPW-20 Nitratireductor sp.
 020_E1-EBD-06 Nitratireductor sp.
100
 021_C2-GNP5-22 Nitratireductor sp.
100
S010
 022_E2-BRE-21 Nitratireductor sp.
100
 023_F1-TOW-17 Nitratiredutor sp.
 024_B1-GNL1-05 Nitratireductor sp.
 025_F1-TOW-02 Roseitalea porphyridii
 026_E1-EBD-05 Roseitalea porphyridii
84
 027_C2-GNP5-04 Roseitalea porphyridii
 028_D1-CHI-03 Roseitalea porphyridii
99
S011
96
 029_F4-SAH-02 Roseitalea porphyridii
 030_C1-SOL-11 Roseitalea porphyridii
100
 031_B1-GNL1-09 Roseitalea porphyridii
 032_E2-BRE-03 Roseitalea porphyridii
100
 033_G1-WW12-03 Roseitalea porphyridii
 034_F3-SA18-12 Roseibium album
100
100
S012
100
 035_G1-WW12-05 Roseibium album
100
 036_F2-STO-04 Roseibium album
S013
 037_E1-EBD-28 Roseibium aggregatum
89
 038_F3-SA18-09 Rhizobiales bacterium
100
S014
 039_G1-WW12-18 Rhizobiales bacterium
S015
 040_F1-TOW-06 Devosia marina
100
S016
100
 041_D1-CHI-27 Devosia sp.
S017
 042_E1-EBD-23 Pelagibacterium sp.
S018
 043_G1-WW12-17 Filomicrobium sp.
 044_D1-CHI-19 Marinovum algicola
 045_G2-EDA-05 Marinovum algicola
 046_G1-WW12-01 Marinovum algicola
 047_B1-GNL1-07 Marinovum algicola
 048_C1-SOL-05 Marinovum algicola
100
S019
 049_F2-STO-08 Marinovum algicola
 050_F3-SA18-07 Marinovum algicola
100
 051_A1-SPW-11 Marinovum algicola
 052_F4-SAH-15 Marinovum algicola
 053_F1-TOW-14 Marinovum algicola
79
Alphaproteobacteria
 054_C2-GNP5-01 Marinovum algicola
 055_B1-GNL1-03 Roseovarius sp.
 056_F1-TOW-12 Roseovarius sp.
54
100
S020
 057_A1-SPW-28 Roseovarius sp.
100
 058_C1-SOL-12 Roseovarius sp.
93
 059_C2-GNP5-06 Roseovarius sp.
100
S021
 060_B1-GNL1-10 Roseovarius sp.
100
S022
 061_E2-BRE-12 Roseovarius indicus
100
S023
 062_F3-SA18-17 Roseovarius sp.
S024
 063_A1-SPW-34 Roseovarius confluentis
100
100
 064_C1-SOL-08 Roseovarius sp.
S025
100
 065_B1-GNL1-15 Roseovarius sp.
S026
 066_E1-EBD-29 Roseocyclus sp.
S027
 067_C1-SOL-10 Silicimonas sp.
48
 068_G1-WW12-40 Amphiplicatus sp.
100
S028
 069_A1-SPW-36 Amphiplicatus sp.
S029
 070_E1-EBD-16 Oceanicaulis sp.
100
S030
 071_D1-CHI-12 Henriciella sp.
100
100
 072_G2-EDA-03 Hyphomonas sp.
100
S031
100
 073_F4-SAH-12 Hyphomonas sp.
S032
 074_A1-SPW-22 Hyphomonas sp.
73
 075_E1-EBD-09 Thalassobaculaceae bacterium
 076_C2-GNP5-14 Thalassobaculaceae bacterium
100
S033
077_F1-TOW-08 Thalassobaculaceae bacterium
 078_D1-CHI-21 Thalassobaculaceae bacterium
100
 079_E1-EBD-18 Thalassobaculum sp.
100
S034
 080_G1-WW12-27 Thalassobaculum sp.
 081_G1-WW12-09 Thalassobaculum sp.
100
100
 082_E2-BRE-05 Thalassobaculum sp.
S035
 083_C2-GNP5-16 Thalassobaculum sp.
100
100
 084_F3-SA18-13 Thalassobaculum sp.
S036
 085_A1-SPW-09 Nisaea sp.
S037
 086_A1-SPW-31 Oceanibaculum nanhaiense
70
 087_E1-EBD-17 Alphaproteobacterium
100
S038
100
 088_F4-SAH-17 Alphaproteobacterium
100
S039
 089_G1-WW12-19 Alphaproteobacterium
100
S040
 090_E1-EBD-10 Marivibrio sp.
S041
 091_F4-SAH-03 Alphaproteobacterium
 092_E1-EBD-22 Alphaproteobacterium
100
S042
94
 093_C2-GNP5-18 Alphaproteobacterium
100
 094_E2-BRE-19 Alphaproteobacterium
 095_D1-CHI-16 Alphaproteobacterium
100
S043
 096_E1-EBD-30 Alphaproteobacterium
46
 097_F2-STO-11 Alphaproteobacterium
 098_C2-GNP5-10 Alphaproteobacterium
S044
 099_A1-SPW-04 Kiloniellaceae bacterium
100
S045
 100_G1-WW12-28 Kiloniellales bacterium
S046
 101_C1-SOL-07 Azospirillaceae bacterium
100
S047
 102_G2-EDA-09 Azospirillaceae bacterium
 103_F4-SAH-28 Rhodospirillaceae bacterium
100
 104_F3-SA18-14 Rhodospirillaceae bacterium
100
100
S048
 105_E2-BRE-14 Rhodospirillaceae bacterium
100
 106_D1-CHI-20 Rhodospirillaceae bacterium
S049
 107_G1-WW12-25 Rhodospirillaceae bacterium
 108_A1-SPW-10 Thalassospira sp.
96
 109_E2-BRE-11 Thalassospira sp.
100
 110_D1-CHI-13 Thalassospira sp.
S050
52
 111_F2-STO-10 Thalassospira sp.
100
 112_F4-SAH-24 Thalassospira sp.
 113_E1-EBD-27 Thalassospira sp.
 114_E2-BRE-06 Rhodospirillales bacterium
100
S051
115_ D1-CHI-22 Rhodospirillales bacterium
100
100
 116_G1-WW12-22 Rhodospirillales bacterium
S052
 117_A1-SPW-30 Rhodospirillales bacterium
100
94
 118_C2-GNP5-11 Rhodospirillales bacterium
95
S053
 119_B1-GNL1-18 Rhodospirillales bacterium
100
100
 120_G2-EDA-12 Rhodospirillales bacterium
99
S054
 121_D1-CHI-11 Rhodospirillales bacterium
S055
 122_G1-WW12-23 Rhodospirillales bacterium
S056
 123_A1-SPW-39 Rhodospirillales bacterium
67
S057
 124_F4-SAH-26 Rhodospirillales bacterium
100
S058
 125_G1-WW12-34 Rhodospirillales bacterium
 126_C2-GNP5-21 Sneathiellaceae bacterium
100
S059
 127_A1-SPW-23 Sneathiellaceae bacterium
51
100
 128_D1-CHI-29 Sneathiellaceae bacterium
S060
 129_A1-SPW-24 Alphaproteobacterium
100
S061
 130_F1-TOW-13 Alphaproteobacterium
S062
 131_G1-WW12-16 Parasphingorhabdus sp.
53
S063
53
 132_G2-EDA-11 Parasphingopyxis sp.
100
S064
 133_E1-EBD-20 Erythrobacter sp.
S065
 134_A1-SPW-19 Sphingopyxis sp.
S066
 135_E2-BRE-18 Rickettsiales bacterium
 136_F4-SAH-18 Salinispheraceae bacterium
 137_F2-STO-09 Salinispheraceae bacterium
100
S067
 138_G2-EDA-07 Salinispheraceae bacterium
 139_C2-GNP5-02 Salinispheraceae bacterium
 140_B1-GNL1-16 Salinispheraceae bacterium
 141_D1-CHI-10 Algiphilus acroporae
 142_E2-BRE-10 Algiphilus acroporae
100
 143_A1-SPW-18 Algiphilus acroporae
 144_G1-WW12-11 Algiphilus acroporae
100
99
 145_E1-EBD-08 Algiphilus acroporae
S068
 146_G3-WIS-03 Algiphilus acroporae
95
Gammaproteobacteria
 147_C1-SOL-06 Algiphilus acroporae
100
 148_F4-SAH-08 Algiphilus acroporae
 149_C2-GNP5-03 Algiphilus acroporae
 150_F1-TOW-05 Algiphilus acroporae
S069
57
 151_A1-SPW-40 Woeseia sp.
 152_D1-CHI-09 Gammaproteobacterium
100
 153_A1-SPW-08 Gammaproteobacterium
100
S070
 154_B1-GNL1-12 Gammaproteobacterium
100
 155_E1-EBD-15 Gammaproteobacterium
100
 156_D1-CHI-30 Gammaproteobacterium
100
100
S071
 157_C2-GNP5-20 Gammaproteobacterium
 158_A1-SPW-37 Gammaproteobacterium
S072
 159_F2-STO-06 Haliea sp.
100
100
S073
100
 160_F4-SAH-25 Haliea sp.
S074
 161_A1-SPW-12 Pseudohaliea sp.
98
 162_A1-SPW-33 Marinobacter salarius
100
S075
 163_F2-STO-07 Marinobacter salarius
100
 164_F1-TOW-16 Marinobacter salarius
S076
 165_G3-WIS-02 Marinobacter sp.
 166_A1-SPW-07 Gammaproteobacterium
 167_D1-CHI-14 Gammaproteobacterium
100
100
S077
 168_F1-TOW-11 Gammaproteobacterium
100
 169_F3-SA18-08 Gammaproteobacterium
100
S078
 170_E1-EBD-25 Gammaproteobacterium
100
 171_B1-GNL1-13 Pseudomonadales bacterium
100
99
 172_C2-GNP5-23 Pseudomonadales bacterium
S079
100
 173_C1-SOL-15 Pseudomonadales bacterium
100
 174_E1-EBD-07 Pseudomonadales bacterium
S080
100
 175_G1-WW12-30 Pseudomonadales bacterium
68
 176_F4-SAH-20 Pseudomonadales bacterium
100
S081
 177_G1-WW12-39 Pseudomonadales bacterium
S082
 178_F1-TOW-18 Alcanivorax xenomutans
Beta-
proteo-
bacteria
100
 179_C2-GNP5-15 Alcanivorax sp.
S083
100
 180_A1-SPW-15 Alcanivorax sp.
47
S084
 181_C2-GNP5-13 Limnobacter sp.
 182_A1-SPW-13 Deltaproteobacterium
 183_F4-SAH-06 Deltaproteobacterium
100
S085
 184_D1-CHI-08 Deltaproteobacterium
100
Delta-
proteo-
bacteria
 185_E2-BRE-13 Deltaproteobacterium
 186_C1-SOL-14 Deltaproteobacterium
100
 187_G1-WW12-31 Sandaracinaceae bacterium
 188_B1-GNL1-17 Sandaracinaceae bacterium
 189_F1-TOW-15 Sandaracinaceae bacterium
100
S086
 190_E1-EBD-04 Sandaracinaceae bacterium
91
 191_G2-EDA-08 Sandaracinaceae bacterium
Sumer-
laeota
 192_C2-GNP5-24 Sandaracinaceae bacterium
S087
 193_F1-TOW-09 Deltaproteobacterium
S088
 194_G1-WW12-26 Candidatus Sumerlaeota bacterium
 195_C2-GNP5-25 Marinoscillum sp.
 196_F4-SAH-07 Marinoscillum sp.
100
S089
 197_D1-CHI-06 Marinoscillum sp.
100
 198_G1-WW12-20 Marinoscillum sp.
S090
99
 199_E1-EBD-14 Marinoscillum sp.
 200_A1-SPW-38 Cyclobacteriaceae bacterium
100
S091
 201_E2-BRE-08 Cyclobacteriaceae bacterium
 202_D1-CHI-25 Cyclobacteriaceae bacterium
100
 203_A1-SPW-25 Cyclobacteriaceae bacterium
73
 204_C1-SOL-17 Cyclobacteriaceae bacterium
 205_E2-BRE-15 Cyclobacteriaceae bacterium
S092
 206_G1-WW12-29 Cyclobacteriaceae bacterium
100
100
 207_D1-CHI-15 Cyclobacteriaceae bacterium
 208_E1-EBD-13 Cyclobacteriaceae bacterium
S093
 209_D1-CHI-02 Ekhidna sp.
100
S094
100
 210_B1-GNL1-04 Ekhidna sp.
S095
 211_E2-BRE-04 Ekhidna sp.
100
S096
 212_F3-SA18-02 Cyclobacteriaceae bacterium
S097
 213_B1-GNL1-19 Imperialibacter sp.
S098
43
 214_G1-WW12-35 Imperialibacter sp.
 215_E2-BRE-17 Imperialibacter sp.
S099
100
100
 216_F4-SAH-14 Imperialibacter sp.
S100
 217_E1-EBD-19 Imperialibacter sp.
S101
 218_F3-SA18-06 Imperialibacter sp.
S102
 219_G1-WW12-36 Cyclobacteriaceae bacterium
100
S103
 220_F3-SA18-15 Cyclobacteriaceae bacterium
68
Bacteroiota
S104
 221_F4-SAH-13 Cyclobacteriaceae bacterium
S105
 222_F3-SA18-10 Cyclobacteriaceae bacterium
S106
 223_A1-SPW-02 Cytophagales bacterium
 224_G1-WW12-38 Cytophagales bacterium
100
100
 225_D1-CHI-05 Cytophagales bacterium
 226_E2-BRE-02 Cytophagales bacterium
S107
100
 227_B1-GNL1-11 Cytophagales bacterium
100
100
 228_F4-SAH-11 Cytophagales bacterium
 229_F2-STO-12 Cytophagales bacterium
S108
 230_F1-TOW-10 Cyclobacteriaceae bacterium
100
S109
100
 231_A1-SPW-14 Cyclobacteriaceae bacterium
S110
100
 232_A1-SPW-26 Cyclobacteriaceae bacterium
 233_A1-SPW-35 Fulvivirga sp.
100
 234_E2-BRE-16 Fulvivirga sp.
69
S111
 235_D1-CHI-31 Fulvivirga sp.
 236_G2-EDA-10 Fulvivirga sp.
100
 237_E1-EBD-03 Fulvivirga sp.
100
S112
 238_F2-STO-13 Fulvivirga sp.
S113
 239_C1-SOL-13 Fulvivirga sp.
100
S114
 240_F4-SAH-22 Cyclobacteriaceae bacterium
 241_D1-CHI-04 Vicingaceae bacterium
100
S115
100
 242_F4-SAH-09 Vicingaceae bacterium
S116
 243_B1-GNL1-06 Vicingaceae bacterium
100
 244_C2-GNP5-19 Vicingaceae bacterium
87
S117
90
 245_A1-SPW-21 Vicingaceae bacterium
100
 246_G1-WW12-37 Vicingaceae bacterium
100
S118
 247_E2-BRE-20 Cryomorphaceae bacterium
S119
100
 248_F3-SA18-03 Owenweeksia sp.
S120
 249_E1-EBD-26 Arenibacter algicola
100
S121
100
 250_A1-SPW-32 Arenibacter sp.
S122
100
 251_D1-CHI-23 Muricauda sp.
S123
100
 252_F1-TOW-07 Muricauda sp.
S124
 253_G1-WW12-15 Phaeodactylibacter sp.
Balneolota
 254_F4-SAH-04 Balneola sp.
100
 255_D1-CHI-07 Balneola sp.
100
 256_C1-SOL-04 Balneola sp.
S125
 257_B1-GNL1-08 Balneola sp.
100
 258_G2-EDA-06 Balneola sp.
100
 259_F2-STO-02 Balneola sp.
 260_F3-SA18-05 Balneola sp.
100
S126
 261_C2-GNP5-12 Balneola sp.
100
S127
 262_F3-SA18-04 Balneolaceae bacterium
Rhodo-
therhm-
ota
100
S128
100
 263_F3-SA18-16 Balneolaceae bacterium
100
 264_C1-SOL-02 Gracilimonas sp.
100
S129
100
 265_E1-EBD-01 Gracilimonas sp.
S130
 266_C2-GNP5-09 Rhodothermales bacterium
S131
 267_F4-SAH-23 Longimicrobiales bacterium
100
S132
 268_A1-SPW-16 Longimicrobiales bacterium
Gemma-
timona-
dota
S133
 269_G1-WW12-21 Gimesia sp.
100
S134
100
 270_C2-GNP5-26 Gimesia chilikensis
S135
100
 271_D1-CHI-24 Gimesia maris
S136
 272_F4-SAH-21 Fuerstia sp.
100
S137
 273_G1-WW12-08 Pirellulaceae bacterium
 274_G1-WW12-14 Pirellulales bacterium
100
S138
100
 275_F4-SAH-29 Pirellulales bacterium
Planctomycetota
 276_A1-SPW-29 Lacipirellulaceae bacterium
100
42
100
S139
100
 277_D1-CHI-18 Lacipirellulaceae bacterium
S140
 278_G1-WW12-12 Botrimarina sp.
 279_G2-EDA-01 Phycisphaerales bacterium
 280_F4-SAH-01 Phycisphaerales bacterium
100
 281_A1-SPW-05 Phycisphaerales bacterium
100
S141
 282_C1-SOL-01 Phycisphaerales bacterium
100
 283_F2-STO-01 Phycisphaerales bacterium
 284_B1-GNL1-02 Phycisphaerales bacterium
100
S142
 285_C2-GNP5-07 Phycisphaerales bacterium
100
S143
 286_G1-WW12-04 Phycisphaerales bacterium
100
S144
 287_G1-WW12-06 Phycisphaerales bacterium
100
100
S145
 288_F2-STO-05 Phycisphaerales bacterium
100
S146
 289_F1-TOW-01 Phycisphaerales bacterium
S147
 290_F3-SA18-11 Phycisphaeraceae bacterium
100
Spiro-
chae-
tota
65
S148
 291_F4-SAH-10 Phycisphaeraceae bacterium
S149
 292_G1-WW12-33 Planctomycetota bacterium
S150
 293_F3-SA18-20 Leptospirales bacterium
100
S151
 294_A1-SPW-03 Leptospiraceae bacterium
S152
 295_F4-SAH-19 Marinisubtilis sp.
100
Actino-
mycetota
 296_B1-GNL1-14 Marinisubtilis pacificus
S153
100
100
 297_E1-EBD-24 Marinisubtilis pacificus
S154
 298_C2-GNP5-17 Pontimonas sp.
100
S155
 299_G1-WW12-32 Acidimicrobiales bacterium
100
100
S156
 300_G1-WW12-13 Acidimicrobiales bacterium
100
S157
 301_E1-EBD-21 Ilumatobacter fluminis
 302_C2-GNP5-05 Miltoncostaeaceae bacterium
100
S158
 303_G1-WW12-10 Miltoncostaeaceae bacterium
Chloro-
felx-
ota
100
 304_F4-SAH-27 Miltoncostaeaceae bacterium
 305_C1-SOL-09 Aggregatilineales bacterium
100
S159
 306_G1-WW12-24 Aggregatilineales bacterium
 307_A1-SPW-01 Coleofasciculus sp.
 308_D1-CHI-01 Coleofasciculus sp.
 309_B1-GNL1-01 Coleofasciculus sp.
 310_C1-SOL-03 Coleofasciculus sp.
 311_C2-GNP5-27 Coleofasciculus sp.
 312_E1-EBD-02 Coleofasciculus sp.
 313_E2-BRE-01 Coleofasciculus sp.
 314_F1-TOW-03 Coleofasciculus chthonoplastes
 315_F2-STO-03 Coleofasciculus chthonoplastes
 316_F3-SA18-01 Coleofasciculus chthonoplastes
 317_F4-SAH-05 Coleofasciculus chthonoplastes
 318_G1-WW12-02 Coleofasciculus sp.
 319_G2-EDA-02 Coleofasciculus sp.
 320_G3-WIS-01 Coleofasciculus sp.
49
Cyano-
bacteriota
100
100
0.20
100
Figure S1A
Species level: 159 different associated bacterial species in the cyanosphere of Coleofasciculus

## Slide 2
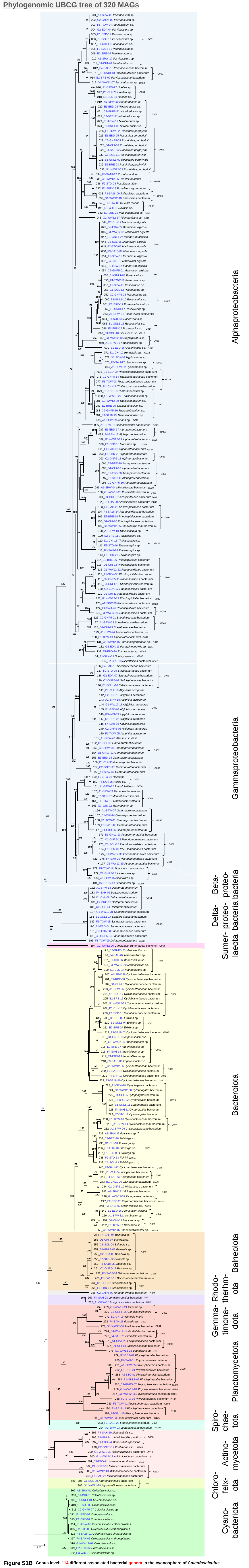

Phylogenomic UBCG tree of 320 MAGs
 001_A1-SPW-06 Parvibaculum sp.
 002_C2-GNP5-08 Parvibaculum sp.
97
 003_F1-TOW-04 Parvibaculum sp.
96
 004_G2-EDA-04 Parvibaculum sp.
 005_E1-EBD-12 Parvibaculum sp.
G001
100
 006_C1-SOL-16 Parvibaculum sp.
 007_D1-CHI-17 Parvibaculum sp.
 008_F3-SA18-19 Parvibaculum sp.
100
 009_E2-BRE-07 Parvibaculum sp.
 010_A1-SPW-17 Parvibaculum sp.
100
100
 011_D1-CHI-26 Parvibaculum sp.
 012_F4-SAH-16 Parvibaculaceae bacterium
98
100
100
G002
 013_F3-SA18-18 Parvibaculaceae bacterium
 014_E2-BRE-09 Parvibaculaceae bacterium
G003
 015_G1-WW12-07 Pyruvatibacter sp.
016_A1-SPW-27 Hoeflea sp.
100
G004
100
 017_D1-CHI-28 Hoeflea sp.
 018_E1-EBD-11 Hoeflea sp.
019_ A1-SPW-20 Nitratireductor sp.
 020_E1-EBD-06 Nitratireductor sp.
100
 021_C2-GNP5-22 Nitratireductor sp.
100
G005
 022_E2-BRE-21 Nitratireductor sp.
100
 023_F1-TOW-17 Nitratiredutor sp.
 024_B1-GNL1-05 Nitratireductor sp.
 025_F1-TOW-02 Roseitalea porphyridii
 026_E1-EBD-05 Roseitalea porphyridii
84
 027_C2-GNP5-04 Roseitalea porphyridii
 028_D1-CHI-03 Roseitalea porphyridii
99
G006
96
 029_F4-SAH-02 Roseitalea porphyridii
 030_C1-SOL-11 Roseitalea porphyridii
100
 031_B1-GNL1-09 Roseitalea porphyridii
 032_E2-BRE-03 Roseitalea porphyridii
100
 033_G1-WW12-03 Roseitalea porphyridii
 034_F3-SA18-12 Roseibium album
100
100
100
 035_G1-WW12-05 Roseibium album
G007
100
 036_F2-STO-04 Roseibium album
 037_E1-EBD-28 Roseibium aggregatum
89
 038_F3-SA18-09 Rhizobiales bacterium
100
G008
 039_G1-WW12-18 Rhizobiales bacterium
 040_F1-TOW-06 Devosia marina
100
G009
100
 041_D1-CHI-27 Devosia sp.
G010
 042_E1-EBD-23 Pelagibacterium sp.
G011
 043_G1-WW12-17 Filomicrobium sp.
 044_D1-CHI-19 Marinovum algicola
 045_G2-EDA-05 Marinovum algicola
 046_G1-WW12-01 Marinovum algicola
 047_B1-GNL1-07 Marinovum algicola
 048_C1-SOL-05 Marinovum algicola
100
G012
 049_F2-STO-08 Marinovum algicola
 050_F3-SA18-07 Marinovum algicola
100
 051_A1-SPW-11 Marinovum algicola
 052_F4-SAH-15 Marinovum algicola
 053_F1-TOW-14 Marinovum algicola
79
Alphaproteobacteria
 054_C2-GNP5-01 Marinovum algicola
 055_B1-GNL1-03 Roseovarius sp.
 056_F1-TOW-12 Roseovarius sp.
54
100
 057_A1-SPW-28 Roseovarius sp.
100
 058_C1-SOL-12 Roseovarius sp.
93
 059_C2-GNP5-06 Roseovarius sp.
100
 060_B1-GNL1-10 Roseovarius sp.
G013
100
 061_E2-BRE-12 Roseovarius indicus
100
 062_F3-SA18-17 Roseovarius sp.
 063_A1-SPW-34 Roseovarius confluentis
100
100
 064_C1-SOL-08 Roseovarius sp.
100
 065_B1-GNL1-15 Roseovarius sp.
G014
 066_E1-EBD-29 Roseocyclus sp.
G015
 067_C1-SOL-10 Silicimonas sp.
48
 068_G1-WW12-40 Amphiplicatus sp.
100
G016
 069_A1-SPW-36 Amphiplicatus sp.
G017
 070_E1-EBD-16 Oceanicaulis sp.
100
G018
 071_D1-CHI-12 Henriciella sp.
100
100
 072_G2-EDA-03 Hyphomonas sp.
100
100
G019
 073_F4-SAH-12 Hyphomonas sp.
 074_A1-SPW-22 Hyphomonas sp.
73
 075_E1-EBD-09 Thalassobaculaceae bacterium
 076_C2-GNP5-14 Thalassobaculaceae bacterium
100
G020
077_F1-TOW-08 Thalassobaculaceae bacterium
 078_D1-CHI-21 Thalassobaculaceae bacterium
100
 079_E1-EBD-18 Thalassobaculum sp.
100
 080_G1-WW12-27 Thalassobaculum sp.
 081_G1-WW12-09 Thalassobaculum sp.
G021
100
100
 082_E2-BRE-05 Thalassobaculum sp.
 083_C2-GNP5-16 Thalassobaculum sp.
100
100
 084_F3-SA18-13 Thalassobaculum sp.
G022
 085_A1-SPW-09 Nisaea sp.
G023
 086_A1-SPW-31 Oceanibaculum nanhaiense
70
 087_E1-EBD-17 Alphaproteobacterium
100
G024
100
 088_F4-SAH-17 Alphaproteobacterium
100
G025
 089_G1-WW12-19 Alphaproteobacterium
100
G026
 090_E1-EBD-10 Marivibrio sp.
G027
 091_F4-SAH-03 Alphaproteobacterium
 092_E1-EBD-22 Alphaproteobacterium
100
G028
94
 093_C2-GNP5-18 Alphaproteobacterium
100
 094_E2-BRE-19 Alphaproteobacterium
 095_D1-CHI-16 Alphaproteobacterium
100
G029
 096_E1-EBD-30 Alphaproteobacterium
46
 097_F2-STO-11 Alphaproteobacterium
 098_C2-GNP5-10 Alphaproteobacterium
G030
 099_A1-SPW-04 Kiloniellaceae bacterium
100
G031
 100_G1-WW12-28 Kiloniellales bacterium
G032
 101_C1-SOL-07 Azospirillaceae bacterium
100
G033
 102_G2-EDA-09 Azospirillaceae bacterium
 103_F4-SAH-28 Rhodospirillaceae bacterium
100
 104_F3-SA18-14 Rhodospirillaceae bacterium
100
100
G034
 105_E2-BRE-14 Rhodospirillaceae bacterium
100
 106_D1-CHI-20 Rhodospirillaceae bacterium
 107_G1-WW12-25 Rhodospirillaceae bacterium
 108_A1-SPW-10 Thalassospira sp.
96
 109_E2-BRE-11 Thalassospira sp.
100
 110_D1-CHI-13 Thalassospira sp.
G035
52
 111_F2-STO-10 Thalassospira sp.
100
 112_F4-SAH-24 Thalassospira sp.
 113_E1-EBD-27 Thalassospira sp.
 114_E2-BRE-06 Rhodospirillales bacterium
100
115_ D1-CHI-22 Rhodospirillales bacterium
100
100
 116_G1-WW12-22 Rhodospirillales bacterium
 117_A1-SPW-30 Rhodospirillales bacterium
100
94
G036
 118_C2-GNP5-11 Rhodospirillales bacterium
95
 119_B1-GNL1-18 Rhodospirillales bacterium
100
100
 120_G2-EDA-12 Rhodospirillales bacterium
99
 121_D1-CHI-11 Rhodospirillales bacterium
G037
 122_G1-WW12-23 Rhodospirillales bacterium
G038
 123_A1-SPW-39 Rhodospirillales bacterium
67
 124_F4-SAH-26 Rhodospirillales bacterium
100
G039
 125_G1-WW12-34 Rhodospirillales bacterium
 126_C2-GNP5-21 Sneathiellaceae bacterium
100
G040
 127_A1-SPW-23 Sneathiellaceae bacterium
51
100
 128_D1-CHI-29 Sneathiellaceae bacterium
G041
 129_A1-SPW-24 Alphaproteobacterium
100
G042
 130_F1-TOW-13 Alphaproteobacterium
G043
 131_G1-WW12-16 Parasphingorhabdus sp.
53
G044
53
 132_G2-EDA-11 Parasphingopyxis sp.
100
G045
 133_E1-EBD-20 Erythrobacter sp.
G046
 134_A1-SPW-19 Sphingopyxis sp.
G047
 135_E2-BRE-18 Rickettsiales bacterium
 136_F4-SAH-18 Salinispheraceae bacterium
 137_F2-STO-09 Salinispheraceae bacterium
100
G048
 138_G2-EDA-07 Salinispheraceae bacterium
 139_C2-GNP5-02 Salinispheraceae bacterium
 140_B1-GNL1-16 Salinispheraceae bacterium
 141_D1-CHI-10 Algiphilus acroporae
 142_E2-BRE-10 Algiphilus acroporae
100
 143_A1-SPW-18 Algiphilus acroporae
 144_G1-WW12-11 Algiphilus acroporae
G049
100
99
 145_E1-EBD-08 Algiphilus acroporae
 146_G3-WIS-03 Algiphilus acroporae
95
Gammaproteobacteria
 147_C1-SOL-06 Algiphilus acroporae
100
 148_F4-SAH-08 Algiphilus acroporae
 149_C2-GNP5-03 Algiphilus acroporae
 150_F1-TOW-05 Algiphilus acroporae
G050
57
 151_A1-SPW-40 Woeseia sp.
 152_D1-CHI-09 Gammaproteobacterium
100
 153_A1-SPW-08 Gammaproteobacterium
100
G051
 154_B1-GNL1-12 Gammaproteobacterium
100
 155_E1-EBD-15 Gammaproteobacterium
100
 156_D1-CHI-30 Gammaproteobacterium
100
100
G052
 157_C2-GNP5-20 Gammaproteobacterium
 158_A1-SPW-37 Gammaproteobacterium
 159_F2-STO-06 Haliea sp.
100
G053
100
100
 160_F4-SAH-25 Haliea sp.
G054
 161_A1-SPW-12 Pseudohaliea sp.
98
 162_A1-SPW-33 Marinobacter salarius
100
 163_F2-STO-07 Marinobacter salarius
G055
100
 164_F1-TOW-16 Marinobacter salarius
 165_G3-WIS-02 Marinobacter sp.
 166_A1-SPW-07 Gammaproteobacterium
 167_D1-CHI-14 Gammaproteobacterium
100
100
G056
 168_F1-TOW-11 Gammaproteobacterium
100
 169_F3-SA18-08 Gammaproteobacterium
100
 170_E1-EBD-25 Gammaproteobacterium
100
 171_B1-GNL1-13 Pseudomonadales bacterium
100
99
 172_C2-GNP5-23 Pseudomonadales bacterium
100
G057
 173_C1-SOL-15 Pseudomonadales bacterium
100
 174_E1-EBD-07 Pseudomonadales bacterium
100
 175_G1-WW12-30 Pseudomonadales bacterium
68
 176_F4-SAH-20 Pseudomonadales bacterium
100
G058
 177_G1-WW12-39 Pseudomonadales bacterium
 178_F1-TOW-18 Alcanivorax xenomutans
Beta-
proteo-
bacteria
100
G059
 179_C2-GNP5-15 Alcanivorax sp.
100
 180_A1-SPW-15 Alcanivorax sp.
47
G060
 181_C2-GNP5-13 Limnobacter sp.
 182_A1-SPW-13 Deltaproteobacterium
 183_F4-SAH-06 Deltaproteobacterium
100
G061
 184_D1-CHI-08 Deltaproteobacterium
100
Delta-
proteo-
bacteria
 185_E2-BRE-13 Deltaproteobacterium
 186_C1-SOL-14 Deltaproteobacterium
100
 187_G1-WW12-31 Sandaracinaceae bacterium
 188_B1-GNL1-17 Sandaracinaceae bacterium
 189_F1-TOW-15 Sandaracinaceae bacterium
100
G062
 190_E1-EBD-04 Sandaracinaceae bacterium
91
 191_G2-EDA-08 Sandaracinaceae bacterium
Sumer-
laeota
 192_C2-GNP5-24 Sandaracinaceae bacterium
G063
 193_F1-TOW-09 Deltaproteobacterium
G064
 194_G1-WW12-26 Candidatus Sumerlaeota bacterium
 195_C2-GNP5-25 Marinoscillum sp.
 196_F4-SAH-07 Marinoscillum sp.
100
G065
 197_D1-CHI-06 Marinoscillum sp.
100
 198_G1-WW12-20 Marinoscillum sp.
99
 199_E1-EBD-14 Marinoscillum sp.
 200_A1-SPW-38 Cyclobacteriaceae bacterium
100
 201_E2-BRE-08 Cyclobacteriaceae bacterium
 202_D1-CHI-25 Cyclobacteriaceae bacterium
100
 203_A1-SPW-25 Cyclobacteriaceae bacterium
73
G066
 204_C1-SOL-17 Cyclobacteriaceae bacterium
 205_E2-BRE-15 Cyclobacteriaceae bacterium
 206_G1-WW12-29 Cyclobacteriaceae bacterium
100
100
 207_D1-CHI-15 Cyclobacteriaceae bacterium
 208_E1-EBD-13 Cyclobacteriaceae bacterium
 209_D1-CHI-02 Ekhidna sp.
100
100
G067
 210_B1-GNL1-04 Ekhidna sp.
 211_E2-BRE-04 Ekhidna sp.
100
G068
 212_F3-SA18-02 Cyclobacteriaceae bacterium
 213_B1-GNL1-19 Imperialibacter sp.
43
 214_G1-WW12-35 Imperialibacter sp.
 215_E2-BRE-17 Imperialibacter sp.
G069
100
100
 216_F4-SAH-14 Imperialibacter sp.
 217_E1-EBD-19 Imperialibacter sp.
 218_F3-SA18-06 Imperialibacter sp.
 219_G1-WW12-36 Cyclobacteriaceae bacterium
100
G070
 220_F3-SA18-15 Cyclobacteriaceae bacterium
68
Bacteroiota
G071
 221_F4-SAH-13 Cyclobacteriaceae bacterium
G072
 222_F3-SA18-10 Cyclobacteriaceae bacterium
 223_A1-SPW-02 Cytophagales bacterium
 224_G1-WW12-38 Cytophagales bacterium
100
100
 225_D1-CHI-05 Cytophagales bacterium
G073
 226_E2-BRE-02 Cytophagales bacterium
100
 227_B1-GNL1-11 Cytophagales bacterium
100
100
 228_F4-SAH-11 Cytophagales bacterium
 229_F2-STO-12 Cytophagales bacterium
 230_F1-TOW-10 Cyclobacteriaceae bacterium
100
100
G074
 231_A1-SPW-14 Cyclobacteriaceae bacterium
100
 232_A1-SPW-26 Cyclobacteriaceae bacterium
 233_A1-SPW-35 Fulvivirga sp.
100
 234_E2-BRE-16 Fulvivirga sp.
69
 235_D1-CHI-31 Fulvivirga sp.
G075
 236_G2-EDA-10 Fulvivirga sp.
100
 237_E1-EBD-03 Fulvivirga sp.
100
 238_F2-STO-13 Fulvivirga sp.
 239_C1-SOL-13 Fulvivirga sp.
100
G076
 240_F4-SAH-22 Cyclobacteriaceae bacterium
 241_D1-CHI-04 Vicingaceae bacterium
100
G077
100
 242_F4-SAH-09 Vicingaceae bacterium
G078
 243_B1-GNL1-06 Vicingaceae bacterium
100
 244_C2-GNP5-19 Vicingaceae bacterium
87
G079
90
 245_A1-SPW-21 Vicingaceae bacterium
100
 246_G1-WW12-37 Vicingaceae bacterium
100
G080
 247_E2-BRE-20 Cryomorphaceae bacterium
G081
100
 248_F3-SA18-03 Owenweeksia sp.
 249_E1-EBD-26 Arenibacter algicola
100
G082
100
 250_A1-SPW-32 Arenibacter sp.
100
 251_D1-CHI-23 Muricauda sp.
G083
100
 252_F1-TOW-07 Muricauda sp.
G084
 253_G1-WW12-15 Phaeodactylibacter sp.
Balneolota
 254_F4-SAH-04 Balneola sp.
100
 255_D1-CHI-07 Balneola sp.
100
 256_C1-SOL-04 Balneola sp.
 257_B1-GNL1-08 Balneola sp.
G085
100
 258_G2-EDA-06 Balneola sp.
100
 259_F2-STO-02 Balneola sp.
 260_F3-SA18-05 Balneola sp.
100
 261_C2-GNP5-12 Balneola sp.
100
G086
 262_F3-SA18-04 Balneolaceae bacterium
Rhodo-
therhm-
ota
100
G087
100
 263_F3-SA18-16 Balneolaceae bacterium
100
 264_C1-SOL-02 Gracilimonas sp.
100
G088
100
 265_E1-EBD-01 Gracilimonas sp.
G089
 266_C2-GNP5-09 Rhodothermales bacterium
G090
 267_F4-SAH-23 Longimicrobiales bacterium
100
G091
 268_A1-SPW-16 Longimicrobiales bacterium
Gemma-
timona-
dota
 269_G1-WW12-21 Gimesia sp.
100
100
G092
 270_C2-GNP5-26 Gimesia chilikensis
100
 271_D1-CHI-24 Gimesia maris
G093
 272_F4-SAH-21 Fuerstia sp.
100
G094
 273_G1-WW12-08 Pirellulaceae bacterium
 274_G1-WW12-14 Pirellulales bacterium
100
G095
100
 275_F4-SAH-29 Pirellulales bacterium
Planctomycetota
 276_A1-SPW-29 Lacipirellulaceae bacterium
100
42
100
G096
100
 277_D1-CHI-18 Lacipirellulaceae bacterium
G097
 278_G1-WW12-12 Botrimarina sp.
 279_G2-EDA-01 Phycisphaerales bacterium
 280_F4-SAH-01 Phycisphaerales bacterium
100
 281_A1-SPW-05 Phycisphaerales bacterium
100
G098
 282_C1-SOL-01 Phycisphaerales bacterium
100
 283_F2-STO-01 Phycisphaerales bacterium
 284_B1-GNL1-02 Phycisphaerales bacterium
100
G099
 285_C2-GNP5-07 Phycisphaerales bacterium
100
G100
 286_G1-WW12-04 Phycisphaerales bacterium
100
G101
 287_G1-WW12-06 Phycisphaerales bacterium
100
100
G102
 288_F2-STO-05 Phycisphaerales bacterium
100
G103
 289_F1-TOW-01 Phycisphaerales bacterium
 290_F3-SA18-11 Phycisphaeraceae bacterium
100
G104
Spiro-
chae-
tota
65
 291_F4-SAH-10 Phycisphaeraceae bacterium
G105
 292_G1-WW12-33 Planctomycetota bacterium
G106
 293_F3-SA18-20 Leptospirales bacterium
100
G107
 294_A1-SPW-03 Leptospiraceae bacterium
 295_F4-SAH-19 Marinisubtilis sp.
100
G108
Actino-
mycetota
 296_B1-GNL1-14 Marinisubtilis pacificus
100
100
 297_E1-EBD-24 Marinisubtilis pacificus
G109
 298_C2-GNP5-17 Pontimonas sp.
100
G110
 299_G1-WW12-32 Acidimicrobiales bacterium
100
100
G111
 300_G1-WW12-13 Acidimicrobiales bacterium
100
G112
 301_E1-EBD-21 Ilumatobacter fluminis
 302_C2-GNP5-05 Miltoncostaeaceae bacterium
100
G113
 303_G1-WW12-10 Miltoncostaeaceae bacterium
Chloro-
felx-
ota
100
 304_F4-SAH-27 Miltoncostaeaceae bacterium
 305_C1-SOL-09 Aggregatilineales bacterium
100
G114
 306_G1-WW12-24 Aggregatilineales bacterium
 307_A1-SPW-01 Coleofasciculus sp.
 308_D1-CHI-01 Coleofasciculus sp.
 309_B1-GNL1-01 Coleofasciculus sp.
 310_C1-SOL-03 Coleofasciculus sp.
 311_C2-GNP5-27 Coleofasciculus sp.
 312_E1-EBD-02 Coleofasciculus sp.
 313_E2-BRE-01 Coleofasciculus sp.
 314_F1-TOW-03 Coleofasciculus chthonoplastes
 315_F2-STO-03 Coleofasciculus chthonoplastes
 316_F3-SA18-01 Coleofasciculus chthonoplastes
 317_F4-SAH-05 Coleofasciculus chthonoplastes
 318_G1-WW12-02 Coleofasciculus sp.
 319_G2-EDA-02 Coleofasciculus sp.
 320_G3-WIS-01 Coleofasciculus sp.
49
Cyano-
bacteriota
100
100
0.20
100
Figure S1B
Genus level: 114 different associated bacterial genera in the cyanosphere of Coleofasciculus

## Slide 3
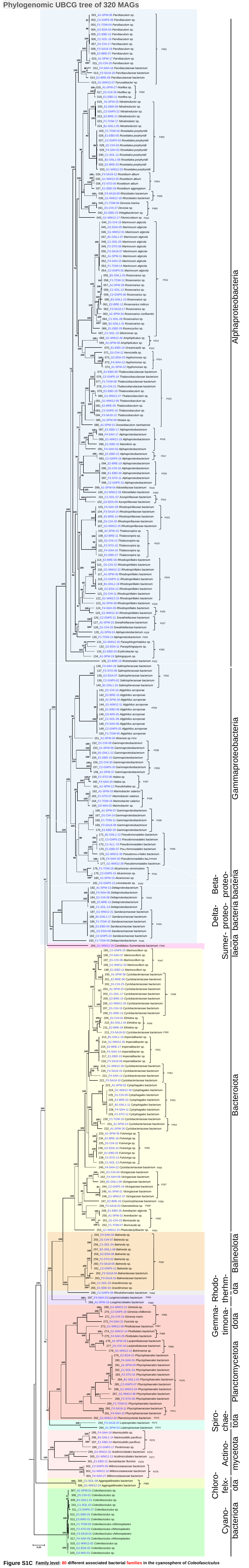

Phylogenomic UBCG tree of 320 MAGs
 001_A1-SPW-06 Parvibaculum sp.
 002_C2-GNP5-08 Parvibaculum sp.
97
 003_F1-TOW-04 Parvibaculum sp.
96
 004_G2-EDA-04 Parvibaculum sp.
 005_E1-EBD-12 Parvibaculum sp.
100
 006_C1-SOL-16 Parvibaculum sp.
 007_D1-CHI-17 Parvibaculum sp.
F001
 008_F3-SA18-19 Parvibaculum sp.
100
 009_E2-BRE-07 Parvibaculum sp.
 010_A1-SPW-17 Parvibaculum sp.
100
100
 011_D1-CHI-26 Parvibaculum sp.
 012_F4-SAH-16 Parvibaculaceae bacterium
98
100
100
 013_F3-SA18-18 Parvibaculaceae bacterium
 014_E2-BRE-09 Parvibaculaceae bacterium
 015_G1-WW12-07 Pyruvatibacter sp.
016_A1-SPW-27 Hoeflea sp.
100
F002
100
 017_D1-CHI-28 Hoeflea sp.
 018_E1-EBD-11 Hoeflea sp.
019_ A1-SPW-20 Nitratireductor sp.
 020_E1-EBD-06 Nitratireductor sp.
100
 021_C2-GNP5-22 Nitratireductor sp.
100
 022_E2-BRE-21 Nitratireductor sp.
100
 023_F1-TOW-17 Nitratiredutor sp.
 024_B1-GNL1-05 Nitratireductor sp.
 025_F1-TOW-02 Roseitalea porphyridii
 026_E1-EBD-05 Roseitalea porphyridii
F003
84
 027_C2-GNP5-04 Roseitalea porphyridii
 028_D1-CHI-03 Roseitalea porphyridii
99
96
 029_F4-SAH-02 Roseitalea porphyridii
 030_C1-SOL-11 Roseitalea porphyridii
100
 031_B1-GNL1-09 Roseitalea porphyridii
 032_E2-BRE-03 Roseitalea porphyridii
100
 033_G1-WW12-03 Roseitalea porphyridii
 034_F3-SA18-12 Roseibium album
100
100
100
 035_G1-WW12-05 Roseibium album
F004
100
 036_F2-STO-04 Roseibium album
 037_E1-EBD-28 Roseibium aggregatum
89
 038_F3-SA18-09 Rhizobiales bacterium
100
F005
 039_G1-WW12-18 Rhizobiales bacterium
 040_F1-TOW-06 Devosia marina
100
F009
100
 041_D1-CHI-27 Devosia sp.
 042_E1-EBD-23 Pelagibacterium sp.
F010
 043_G1-WW12-17 Filomicrobium sp.
 044_D1-CHI-19 Marinovum algicola
 045_G2-EDA-05 Marinovum algicola
 046_G1-WW12-01 Marinovum algicola
 047_B1-GNL1-07 Marinovum algicola
 048_C1-SOL-05 Marinovum algicola
100
 049_F2-STO-08 Marinovum algicola
 050_F3-SA18-07 Marinovum algicola
100
 051_A1-SPW-11 Marinovum algicola
 052_F4-SAH-15 Marinovum algicola
 053_F1-TOW-14 Marinovum algicola
79
Alphaproteobacteria
 054_C2-GNP5-01 Marinovum algicola
 055_B1-GNL1-03 Roseovarius sp.
F011
 056_F1-TOW-12 Roseovarius sp.
54
100
 057_A1-SPW-28 Roseovarius sp.
100
 058_C1-SOL-12 Roseovarius sp.
93
 059_C2-GNP5-06 Roseovarius sp.
100
 060_B1-GNL1-10 Roseovarius sp.
100
 061_E2-BRE-12 Roseovarius indicus
100
 062_F3-SA18-17 Roseovarius sp.
 063_A1-SPW-34 Roseovarius confluentis
100
100
 064_C1-SOL-08 Roseovarius sp.
100
 065_B1-GNL1-15 Roseovarius sp.
 066_E1-EBD-29 Roseocyclus sp.
 067_C1-SOL-10 Silicimonas sp.
48
 068_G1-WW12-40 Amphiplicatus sp.
100
F012
 069_A1-SPW-36 Amphiplicatus sp.
F013
 070_E1-EBD-16 Oceanicaulis sp.
100
 071_D1-CHI-12 Henriciella sp.
100
100
 072_G2-EDA-03 Hyphomonas sp.
100
F014
100
 073_F4-SAH-12 Hyphomonas sp.
 074_A1-SPW-22 Hyphomonas sp.
73
 075_E1-EBD-09 Thalassobaculaceae bacterium
 076_C2-GNP5-14 Thalassobaculaceae bacterium
100
077_F1-TOW-08 Thalassobaculaceae bacterium
 078_D1-CHI-21 Thalassobaculaceae bacterium
100
 079_E1-EBD-18 Thalassobaculum sp.
100
 080_G1-WW12-27 Thalassobaculum sp.
F015
 081_G1-WW12-09 Thalassobaculum sp.
100
100
 082_E2-BRE-05 Thalassobaculum sp.
 083_C2-GNP5-16 Thalassobaculum sp.
100
100
 084_F3-SA18-13 Thalassobaculum sp.
 085_A1-SPW-09 Nisaea sp.
 086_A1-SPW-31 Oceanibaculum nanhaiense
70
 087_E1-EBD-17 Alphaproteobacterium
100
100
 088_F4-SAH-17 Alphaproteobacterium
F016
100
 089_G1-WW12-19 Alphaproteobacterium
100
 090_E1-EBD-10 Marivibrio sp.
 091_F4-SAH-03 Alphaproteobacterium
 092_E1-EBD-22 Alphaproteobacterium
100
F017
94
 093_C2-GNP5-18 Alphaproteobacterium
100
 094_E2-BRE-19 Alphaproteobacterium
 095_D1-CHI-16 Alphaproteobacterium
100
F018
 096_E1-EBD-30 Alphaproteobacterium
46
 097_F2-STO-11 Alphaproteobacterium
 098_C2-GNP5-10 Alphaproteobacterium
F019
 099_A1-SPW-04 Kiloniellaceae bacterium
100
F020
 100_G1-WW12-28 Kiloniellales bacterium
 101_C1-SOL-07 Azospirillaceae bacterium
100
F021
 102_G2-EDA-09 Azospirillaceae bacterium
 103_F4-SAH-28 Rhodospirillaceae bacterium
100
 104_F3-SA18-14 Rhodospirillaceae bacterium
100
100
F022
 105_E2-BRE-14 Rhodospirillaceae bacterium
100
 106_D1-CHI-20 Rhodospirillaceae bacterium
 107_G1-WW12-25 Rhodospirillaceae bacterium
 108_A1-SPW-10 Thalassospira sp.
96
 109_E2-BRE-11 Thalassospira sp.
100
 110_D1-CHI-13 Thalassospira sp.
F023
52
 111_F2-STO-10 Thalassospira sp.
100
 112_F4-SAH-24 Thalassospira sp.
 113_E1-EBD-27 Thalassospira sp.
 114_E2-BRE-06 Rhodospirillales bacterium
100
115_ D1-CHI-22 Rhodospirillales bacterium
100
100
 116_G1-WW12-22 Rhodospirillales bacterium
 117_A1-SPW-30 Rhodospirillales bacterium
100
94
F024
 118_C2-GNP5-11 Rhodospirillales bacterium
95
 119_B1-GNL1-18 Rhodospirillales bacterium
100
100
 120_G2-EDA-12 Rhodospirillales bacterium
99
 121_D1-CHI-11 Rhodospirillales bacterium
 122_G1-WW12-23 Rhodospirillales bacterium
F025
 123_A1-SPW-39 Rhodospirillales bacterium
67
 124_F4-SAH-26 Rhodospirillales bacterium
100
F026
 125_G1-WW12-34 Rhodospirillales bacterium
 126_C2-GNP5-21 Sneathiellaceae bacterium
100
F027
 127_A1-SPW-23 Sneathiellaceae bacterium
51
100
 128_D1-CHI-29 Sneathiellaceae bacterium
F028
 129_A1-SPW-24 Alphaproteobacterium
100
F029
 130_F1-TOW-13 Alphaproteobacterium
 131_G1-WW12-16 Parasphingorhabdus sp.
53
53
 132_G2-EDA-11 Parasphingopyxis sp.
F030
100
 133_E1-EBD-20 Erythrobacter sp.
 134_A1-SPW-19 Sphingopyxis sp.
F031
 135_E2-BRE-18 Rickettsiales bacterium
 136_F4-SAH-18 Salinispheraceae bacterium
 137_F2-STO-09 Salinispheraceae bacterium
100
F032
 138_G2-EDA-07 Salinispheraceae bacterium
 139_C2-GNP5-02 Salinispheraceae bacterium
 140_B1-GNL1-16 Salinispheraceae bacterium
 141_D1-CHI-10 Algiphilus acroporae
 142_E2-BRE-10 Algiphilus acroporae
100
 143_A1-SPW-18 Algiphilus acroporae
 144_G1-WW12-11 Algiphilus acroporae
F033
100
99
 145_E1-EBD-08 Algiphilus acroporae
 146_G3-WIS-03 Algiphilus acroporae
95
Gammaproteobacteria
 147_C1-SOL-06 Algiphilus acroporae
100
 148_F4-SAH-08 Algiphilus acroporae
 149_C2-GNP5-03 Algiphilus acroporae
 150_F1-TOW-05 Algiphilus acroporae
F034
57
 151_A1-SPW-40 Woeseia sp.
 152_D1-CHI-09 Gammaproteobacterium
100
 153_A1-SPW-08 Gammaproteobacterium
100
F035
 154_B1-GNL1-12 Gammaproteobacterium
100
 155_E1-EBD-15 Gammaproteobacterium
100
 156_D1-CHI-30 Gammaproteobacterium
100
100
F036
 157_C2-GNP5-20 Gammaproteobacterium
 158_A1-SPW-37 Gammaproteobacterium
 159_F2-STO-06 Haliea sp.
100
100
F037
100
 160_F4-SAH-25 Haliea sp.
 161_A1-SPW-12 Pseudohaliea sp.
98
 162_A1-SPW-33 Marinobacter salarius
100
 163_F2-STO-07 Marinobacter salarius
F038
100
 164_F1-TOW-16 Marinobacter salarius
 165_G3-WIS-02 Marinobacter sp.
 166_A1-SPW-07 Gammaproteobacterium
 167_D1-CHI-14 Gammaproteobacterium
100
100
F039
 168_F1-TOW-11 Gammaproteobacterium
100
 169_F3-SA18-08 Gammaproteobacterium
100
 170_E1-EBD-25 Gammaproteobacterium
100
 171_B1-GNL1-13 Pseudomonadales bacterium
100
99
 172_C2-GNP5-23 Pseudomonadales bacterium
100
 173_C1-SOL-15 Pseudomonadales bacterium
100
F040
 174_E1-EBD-07 Pseudomonadales bacterium
100
 175_G1-WW12-30 Pseudomonadales bacterium
68
 176_F4-SAH-20 Pseudomonadales bacterium
100
 177_G1-WW12-39 Pseudomonadales bacterium
 178_F1-TOW-18 Alcanivorax xenomutans
Beta-
proteo-
bacteria
100
F041
 179_C2-GNP5-15 Alcanivorax sp.
100
 180_A1-SPW-15 Alcanivorax sp.
47
F042
 181_C2-GNP5-13 Limnobacter sp.
 182_A1-SPW-13 Deltaproteobacterium
 183_F4-SAH-06 Deltaproteobacterium
100
F043
 184_D1-CHI-08 Deltaproteobacterium
100
Delta-
proteo-
bacteria
 185_E2-BRE-13 Deltaproteobacterium
 186_C1-SOL-14 Deltaproteobacterium
100
 187_G1-WW12-31 Sandaracinaceae bacterium
 188_B1-GNL1-17 Sandaracinaceae bacterium
 189_F1-TOW-15 Sandaracinaceae bacterium
100
F044
 190_E1-EBD-04 Sandaracinaceae bacterium
91
 191_G2-EDA-08 Sandaracinaceae bacterium
Sumer-
laeota
 192_C2-GNP5-24 Sandaracinaceae bacterium
F045
 193_F1-TOW-09 Deltaproteobacterium
F046
 194_G1-WW12-26 Candidatus Sumerlaeota bacterium
 195_C2-GNP5-25 Marinoscillum sp.
 196_F4-SAH-07 Marinoscillum sp.
100
F047
 197_D1-CHI-06 Marinoscillum sp.
100
 198_G1-WW12-20 Marinoscillum sp.
99
 199_E1-EBD-14 Marinoscillum sp.
 200_A1-SPW-38 Cyclobacteriaceae bacterium
100
 201_E2-BRE-08 Cyclobacteriaceae bacterium
 202_D1-CHI-25 Cyclobacteriaceae bacterium
100
 203_A1-SPW-25 Cyclobacteriaceae bacterium
73
F048
 204_C1-SOL-17 Cyclobacteriaceae bacterium
 205_E2-BRE-15 Cyclobacteriaceae bacterium
 206_G1-WW12-29 Cyclobacteriaceae bacterium
100
100
 207_D1-CHI-15 Cyclobacteriaceae bacterium
 208_E1-EBD-13 Cyclobacteriaceae bacterium
 209_D1-CHI-02 Ekhidna sp.
100
100
F049
 210_B1-GNL1-04 Ekhidna sp.
 211_E2-BRE-04 Ekhidna sp.
100
F050
 212_F3-SA18-02 Cyclobacteriaceae bacterium
 213_B1-GNL1-19 Imperialibacter sp.
43
 214_G1-WW12-35 Imperialibacter sp.
 215_E2-BRE-17 Imperialibacter sp.
F051
100
100
 216_F4-SAH-14 Imperialibacter sp.
 217_E1-EBD-19 Imperialibacter sp.
 218_F3-SA18-06 Imperialibacter sp.
 219_G1-WW12-36 Cyclobacteriaceae bacterium
100
 220_F3-SA18-15 Cyclobacteriaceae bacterium
68
F052
Bacteroiota
 221_F4-SAH-13 Cyclobacteriaceae bacterium
 222_F3-SA18-10 Cyclobacteriaceae bacterium
 223_A1-SPW-02 Cytophagales bacterium
 224_G1-WW12-38 Cytophagales bacterium
100
100
 225_D1-CHI-05 Cytophagales bacterium
F053
 226_E2-BRE-02 Cytophagales bacterium
100
 227_B1-GNL1-11 Cytophagales bacterium
100
100
 228_F4-SAH-11 Cytophagales bacterium
 229_F2-STO-12 Cytophagales bacterium
 230_F1-TOW-10 Cyclobacteriaceae bacterium
100
100
F054
 231_A1-SPW-14 Cyclobacteriaceae bacterium
100
 232_A1-SPW-26 Cyclobacteriaceae bacterium
 233_A1-SPW-35 Fulvivirga sp.
100
 234_E2-BRE-16 Fulvivirga sp.
69
 235_D1-CHI-31 Fulvivirga sp.
F055
 236_G2-EDA-10 Fulvivirga sp.
100
 237_E1-EBD-03 Fulvivirga sp.
100
 238_F2-STO-13 Fulvivirga sp.
 239_C1-SOL-13 Fulvivirga sp.
100
F056
 240_F4-SAH-22 Cyclobacteriaceae bacterium
 241_D1-CHI-04 Vicingaceae bacterium
100
100
 242_F4-SAH-09 Vicingaceae bacterium
 243_B1-GNL1-06 Vicingaceae bacterium
F057
100
 244_C2-GNP5-19 Vicingaceae bacterium
87
90
 245_A1-SPW-21 Vicingaceae bacterium
100
 246_G1-WW12-37 Vicingaceae bacterium
100
F058
 247_E2-BRE-20 Cryomorphaceae bacterium
F059
100
 248_F3-SA18-03 Owenweeksia sp.
 249_E1-EBD-26 Arenibacter algicola
100
100
 250_A1-SPW-32 Arenibacter sp.
F060
100
 251_D1-CHI-23 Muricauda sp.
100
 252_F1-TOW-07 Muricauda sp.
F061
 253_G1-WW12-15 Phaeodactylibacter sp.
Balneolota
 254_F4-SAH-04 Balneola sp.
100
 255_D1-CHI-07 Balneola sp.
100
 256_C1-SOL-04 Balneola sp.
 257_B1-GNL1-08 Balneola sp.
100
 258_G2-EDA-06 Balneola sp.
100
 259_F2-STO-02 Balneola sp.
F062
 260_F3-SA18-05 Balneola sp.
100
 261_C2-GNP5-12 Balneola sp.
100
 262_F3-SA18-04 Balneolaceae bacterium
Rhodo-
therhm-
ota
100
100
 263_F3-SA18-16 Balneolaceae bacterium
100
 264_C1-SOL-02 Gracilimonas sp.
100
100
 265_E1-EBD-01 Gracilimonas sp.
F063
 266_C2-GNP5-09 Rhodothermales bacterium
F064
 267_F4-SAH-23 Longimicrobiales bacterium
100
F065
 268_A1-SPW-16 Longimicrobiales bacterium
Gemma-
timona-
dota
 269_G1-WW12-21 Gimesia sp.
100
100
 270_C2-GNP5-26 Gimesia chilikensis
F066
100
 271_D1-CHI-24 Gimesia maris
 272_F4-SAH-21 Fuerstia sp.
100
F067
 273_G1-WW12-08 Pirellulaceae bacterium
 274_G1-WW12-14 Pirellulales bacterium
100
F068
100
 275_F4-SAH-29 Pirellulales bacterium
Planctomycetota
 276_A1-SPW-29 Lacipirellulaceae bacterium
100
42
100
100
F069
 277_D1-CHI-18 Lacipirellulaceae bacterium
 278_G1-WW12-12 Botrimarina sp.
 279_G2-EDA-01 Phycisphaerales bacterium
 280_F4-SAH-01 Phycisphaerales bacterium
100
 281_A1-SPW-05 Phycisphaerales bacterium
100
 282_C1-SOL-01 Phycisphaerales bacterium
100
 283_F2-STO-01 Phycisphaerales bacterium
F070
 284_B1-GNL1-02 Phycisphaerales bacterium
100
 285_C2-GNP5-07 Phycisphaerales bacterium
100
 286_G1-WW12-04 Phycisphaerales bacterium
100
 287_G1-WW12-06 Phycisphaerales bacterium
100
100
 288_F2-STO-05 Phycisphaerales bacterium
100
 289_F1-TOW-01 Phycisphaerales bacterium
 290_F3-SA18-11 Phycisphaeraceae bacterium
100
F071
Spiro-
chae-
tota
65
 291_F4-SAH-10 Phycisphaeraceae bacterium
F072
 292_G1-WW12-33 Planctomycetota bacterium
F073
 293_F3-SA18-20 Leptospirales bacterium
100
F074
 294_A1-SPW-03 Leptospiraceae bacterium
 295_F4-SAH-19 Marinisubtilis sp.
100
Actino-
mycetota
 296_B1-GNL1-14 Marinisubtilis pacificus
F075
100
100
 297_E1-EBD-24 Marinisubtilis pacificus
 298_C2-GNP5-17 Pontimonas sp.
100
F076
 299_G1-WW12-32 Acidimicrobiales bacterium
100
100
F077
 300_G1-WW12-13 Acidimicrobiales bacterium
100
F078
 301_E1-EBD-21 Ilumatobacter fluminis
 302_C2-GNP5-05 Miltoncostaeaceae bacterium
100
F079
 303_G1-WW12-10 Miltoncostaeaceae bacterium
Chloro-
felx-
ota
100
 304_F4-SAH-27 Miltoncostaeaceae bacterium
 305_C1-SOL-09 Aggregatilineales bacterium
100
F080
 306_G1-WW12-24 Aggregatilineales bacterium
 307_A1-SPW-01 Coleofasciculus sp.
 308_D1-CHI-01 Coleofasciculus sp.
 309_B1-GNL1-01 Coleofasciculus sp.
 310_C1-SOL-03 Coleofasciculus sp.
 311_C2-GNP5-27 Coleofasciculus sp.
 312_E1-EBD-02 Coleofasciculus sp.
 313_E2-BRE-01 Coleofasciculus sp.
 314_F1-TOW-03 Coleofasciculus chthonoplastes
 315_F2-STO-03 Coleofasciculus chthonoplastes
 316_F3-SA18-01 Coleofasciculus chthonoplastes
 317_F4-SAH-05 Coleofasciculus chthonoplastes
 318_G1-WW12-02 Coleofasciculus sp.
 319_G2-EDA-02 Coleofasciculus sp.
 320_G3-WIS-01 Coleofasciculus sp.
49
Cyano-
bacteriota
100
100
0.20
100
Figure S1C
Family level: 80 different associated bacterial families in the cyanosphere of Coleofasciculus

## Slide 4
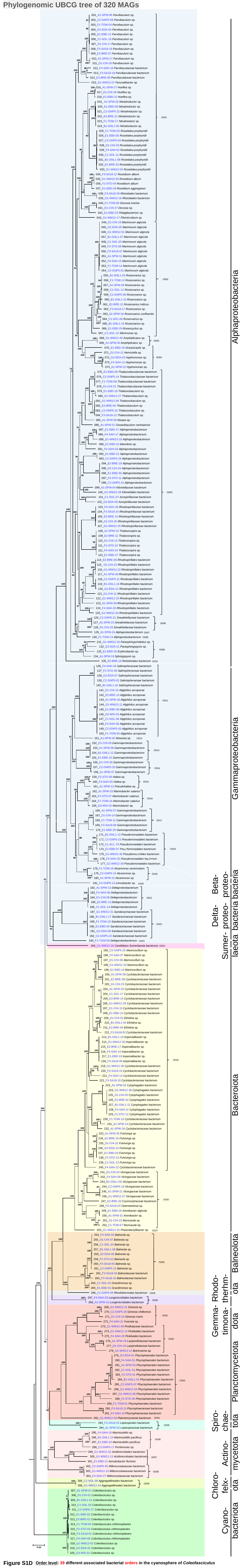

Phylogenomic UBCG tree of 320 MAGs
 001_A1-SPW-06 Parvibaculum sp.
 002_C2-GNP5-08 Parvibaculum sp.
97
 003_F1-TOW-04 Parvibaculum sp.
96
 004_G2-EDA-04 Parvibaculum sp.
 005_E1-EBD-12 Parvibaculum sp.
100
 006_C1-SOL-16 Parvibaculum sp.
 007_D1-CHI-17 Parvibaculum sp.
 008_F3-SA18-19 Parvibaculum sp.
100
 009_E2-BRE-07 Parvibaculum sp.
 010_A1-SPW-17 Parvibaculum sp.
100
100
 011_D1-CHI-26 Parvibaculum sp.
 012_F4-SAH-16 Parvibaculaceae bacterium
98
100
100
 013_F3-SA18-18 Parvibaculaceae bacterium
 014_E2-BRE-09 Parvibaculaceae bacterium
 015_G1-WW12-07 Pyruvatibacter sp.
016_A1-SPW-27 Hoeflea sp.
100
100
 017_D1-CHI-28 Hoeflea sp.
 018_E1-EBD-11 Hoeflea sp.
019_ A1-SPW-20 Nitratireductor sp.
 020_E1-EBD-06 Nitratireductor sp.
100
 021_C2-GNP5-22 Nitratireductor sp.
100
O001
 022_E2-BRE-21 Nitratireductor sp.
100
 023_F1-TOW-17 Nitratiredutor sp.
 024_B1-GNL1-05 Nitratireductor sp.
 025_F1-TOW-02 Roseitalea porphyridii
 026_E1-EBD-05 Roseitalea porphyridii
84
 027_C2-GNP5-04 Roseitalea porphyridii
 028_D1-CHI-03 Roseitalea porphyridii
99
96
 029_F4-SAH-02 Roseitalea porphyridii
 030_C1-SOL-11 Roseitalea porphyridii
100
 031_B1-GNL1-09 Roseitalea porphyridii
 032_E2-BRE-03 Roseitalea porphyridii
100
 033_G1-WW12-03 Roseitalea porphyridii
 034_F3-SA18-12 Roseibium album
100
100
100
 035_G1-WW12-05 Roseibium album
100
 036_F2-STO-04 Roseibium album
 037_E1-EBD-28 Roseibium aggregatum
89
 038_F3-SA18-09 Rhizobiales bacterium
100
 039_G1-WW12-18 Rhizobiales bacterium
 040_F1-TOW-06 Devosia marina
100
100
 041_D1-CHI-27 Devosia sp.
 042_E1-EBD-23 Pelagibacterium sp.
 043_G1-WW12-17 Filomicrobium sp.
 044_D1-CHI-19 Marinovum algicola
 045_G2-EDA-05 Marinovum algicola
 046_G1-WW12-01 Marinovum algicola
 047_B1-GNL1-07 Marinovum algicola
 048_C1-SOL-05 Marinovum algicola
100
 049_F2-STO-08 Marinovum algicola
 050_F3-SA18-07 Marinovum algicola
100
 051_A1-SPW-11 Marinovum algicola
 052_F4-SAH-15 Marinovum algicola
 053_F1-TOW-14 Marinovum algicola
79
Alphaproteobacteria
 054_C2-GNP5-01 Marinovum algicola
 055_B1-GNL1-03 Roseovarius sp.
O002
 056_F1-TOW-12 Roseovarius sp.
54
100
 057_A1-SPW-28 Roseovarius sp.
100
 058_C1-SOL-12 Roseovarius sp.
93
 059_C2-GNP5-06 Roseovarius sp.
100
 060_B1-GNL1-10 Roseovarius sp.
100
 061_E2-BRE-12 Roseovarius indicus
100
 062_F3-SA18-17 Roseovarius sp.
 063_A1-SPW-34 Roseovarius confluentis
100
100
 064_C1-SOL-08 Roseovarius sp.
100
 065_B1-GNL1-15 Roseovarius sp.
 066_E1-EBD-29 Roseocyclus sp.
 067_C1-SOL-10 Silicimonas sp.
48
 068_G1-WW12-40 Amphiplicatus sp.
100
O003
 069_A1-SPW-36 Amphiplicatus sp.
 070_E1-EBD-16 Oceanicaulis sp.
100
 071_D1-CHI-12 Henriciella sp.
100
100
O004
 072_G2-EDA-03 Hyphomonas sp.
100
100
 073_F4-SAH-12 Hyphomonas sp.
 074_A1-SPW-22 Hyphomonas sp.
73
 075_E1-EBD-09 Thalassobaculaceae bacterium
 076_C2-GNP5-14 Thalassobaculaceae bacterium
100
077_F1-TOW-08 Thalassobaculaceae bacterium
 078_D1-CHI-21 Thalassobaculaceae bacterium
100
 079_E1-EBD-18 Thalassobaculum sp.
100
 080_G1-WW12-27 Thalassobaculum sp.
 081_G1-WW12-09 Thalassobaculum sp.
100
100
 082_E2-BRE-05 Thalassobaculum sp.
 083_C2-GNP5-16 Thalassobaculum sp.
100
100
 084_F3-SA18-13 Thalassobaculum sp.
 085_A1-SPW-09 Nisaea sp.
 086_A1-SPW-31 Oceanibaculum nanhaiense
70
 087_E1-EBD-17 Alphaproteobacterium
100
100
 088_F4-SAH-17 Alphaproteobacterium
100
 089_G1-WW12-19 Alphaproteobacterium
100
 090_E1-EBD-10 Marivibrio sp.
 091_F4-SAH-03 Alphaproteobacterium
 092_E1-EBD-22 Alphaproteobacterium
100
94
 093_C2-GNP5-18 Alphaproteobacterium
100
 094_E2-BRE-19 Alphaproteobacterium
 095_D1-CHI-16 Alphaproteobacterium
100
 096_E1-EBD-30 Alphaproteobacterium
46
 097_F2-STO-11 Alphaproteobacterium
 098_C2-GNP5-10 Alphaproteobacterium
 099_A1-SPW-04 Kiloniellaceae bacterium
100
O005
 100_G1-WW12-28 Kiloniellales bacterium
 101_C1-SOL-07 Azospirillaceae bacterium
100
 102_G2-EDA-09 Azospirillaceae bacterium
 103_F4-SAH-28 Rhodospirillaceae bacterium
100
 104_F3-SA18-14 Rhodospirillaceae bacterium
100
100
 105_E2-BRE-14 Rhodospirillaceae bacterium
100
 106_D1-CHI-20 Rhodospirillaceae bacterium
 107_G1-WW12-25 Rhodospirillaceae bacterium
 108_A1-SPW-10 Thalassospira sp.
96
 109_E2-BRE-11 Thalassospira sp.
100
 110_D1-CHI-13 Thalassospira sp.
52
 111_F2-STO-10 Thalassospira sp.
100
 112_F4-SAH-24 Thalassospira sp.
 113_E1-EBD-27 Thalassospira sp.
 114_E2-BRE-06 Rhodospirillales bacterium
100
115_ D1-CHI-22 Rhodospirillales bacterium
100
100
 116_G1-WW12-22 Rhodospirillales bacterium
 117_A1-SPW-30 Rhodospirillales bacterium
100
94
 118_C2-GNP5-11 Rhodospirillales bacterium
95
 119_B1-GNL1-18 Rhodospirillales bacterium
100
100
 120_G2-EDA-12 Rhodospirillales bacterium
99
 121_D1-CHI-11 Rhodospirillales bacterium
 122_G1-WW12-23 Rhodospirillales bacterium
 123_A1-SPW-39 Rhodospirillales bacterium
67
 124_F4-SAH-26 Rhodospirillales bacterium
100
 125_G1-WW12-34 Rhodospirillales bacterium
 126_C2-GNP5-21 Sneathiellaceae bacterium
100
O006
 127_A1-SPW-23 Sneathiellaceae bacterium
51
100
 128_D1-CHI-29 Sneathiellaceae bacterium
O007
 129_A1-SPW-24 Alphaproteobacterium
100
O008
 130_F1-TOW-13 Alphaproteobacterium
 131_G1-WW12-16 Parasphingorhabdus sp.
53
53
 132_G2-EDA-11 Parasphingopyxis sp.
O009
100
 133_E1-EBD-20 Erythrobacter sp.
 134_A1-SPW-19 Sphingopyxis sp.
O010
 135_E2-BRE-18 Rickettsiales bacterium
 136_F4-SAH-18 Salinispheraceae bacterium
 137_F2-STO-09 Salinispheraceae bacterium
100
 138_G2-EDA-07 Salinispheraceae bacterium
 139_C2-GNP5-02 Salinispheraceae bacterium
 140_B1-GNL1-16 Salinispheraceae bacterium
 141_D1-CHI-10 Algiphilus acroporae
 142_E2-BRE-10 Algiphilus acroporae
100
O011
 143_A1-SPW-18 Algiphilus acroporae
 144_G1-WW12-11 Algiphilus acroporae
100
99
 145_E1-EBD-08 Algiphilus acroporae
 146_G3-WIS-03 Algiphilus acroporae
95
Gammaproteobacteria
 147_C1-SOL-06 Algiphilus acroporae
100
 148_F4-SAH-08 Algiphilus acroporae
 149_C2-GNP5-03 Algiphilus acroporae
 150_F1-TOW-05 Algiphilus acroporae
57
O012
 151_A1-SPW-40 Woeseia sp.
 152_D1-CHI-09 Gammaproteobacterium
100
 153_A1-SPW-08 Gammaproteobacterium
100
O013
 154_B1-GNL1-12 Gammaproteobacterium
100
 155_E1-EBD-15 Gammaproteobacterium
100
 156_D1-CHI-30 Gammaproteobacterium
100
100
O014
 157_C2-GNP5-20 Gammaproteobacterium
 158_A1-SPW-37 Gammaproteobacterium
 159_F2-STO-06 Haliea sp.
100
100
O015
100
 160_F4-SAH-25 Haliea sp.
 161_A1-SPW-12 Pseudohaliea sp.
98
 162_A1-SPW-33 Marinobacter salarius
100
 163_F2-STO-07 Marinobacter salarius
O016
100
 164_F1-TOW-16 Marinobacter salarius
 165_G3-WIS-02 Marinobacter sp.
 166_A1-SPW-07 Gammaproteobacterium
 167_D1-CHI-14 Gammaproteobacterium
100
100
O017
 168_F1-TOW-11 Gammaproteobacterium
100
 169_F3-SA18-08 Gammaproteobacterium
100
 170_E1-EBD-25 Gammaproteobacterium
100
 171_B1-GNL1-13 Pseudomonadales bacterium
100
99
 172_C2-GNP5-23 Pseudomonadales bacterium
100
 173_C1-SOL-15 Pseudomonadales bacterium
100
O018
 174_E1-EBD-07 Pseudomonadales bacterium
100
 175_G1-WW12-30 Pseudomonadales bacterium
68
 176_F4-SAH-20 Pseudomonadales bacterium
100
 177_G1-WW12-39 Pseudomonadales bacterium
 178_F1-TOW-18 Alcanivorax xenomutans
Beta-
proteo-
bacteria
100
O019
 179_C2-GNP5-15 Alcanivorax sp.
100
 180_A1-SPW-15 Alcanivorax sp.
47
O020
 181_C2-GNP5-13 Limnobacter sp.
 182_A1-SPW-13 Deltaproteobacterium
 183_F4-SAH-06 Deltaproteobacterium
100
O021
 184_D1-CHI-08 Deltaproteobacterium
100
Delta-
proteo-
bacteria
 185_E2-BRE-13 Deltaproteobacterium
 186_C1-SOL-14 Deltaproteobacterium
100
 187_G1-WW12-31 Sandaracinaceae bacterium
 188_B1-GNL1-17 Sandaracinaceae bacterium
 189_F1-TOW-15 Sandaracinaceae bacterium
100
O022
 190_E1-EBD-04 Sandaracinaceae bacterium
91
 191_G2-EDA-08 Sandaracinaceae bacterium
Sumer-
laeota
 192_C2-GNP5-24 Sandaracinaceae bacterium
O023
 193_F1-TOW-09 Deltaproteobacterium
O024
 194_G1-WW12-26 Candidatus Sumerlaeota bacterium
 195_C2-GNP5-25 Marinoscillum sp.
 196_F4-SAH-07 Marinoscillum sp.
100
 197_D1-CHI-06 Marinoscillum sp.
100
 198_G1-WW12-20 Marinoscillum sp.
99
 199_E1-EBD-14 Marinoscillum sp.
 200_A1-SPW-38 Cyclobacteriaceae bacterium
100
 201_E2-BRE-08 Cyclobacteriaceae bacterium
 202_D1-CHI-25 Cyclobacteriaceae bacterium
100
 203_A1-SPW-25 Cyclobacteriaceae bacterium
73
 204_C1-SOL-17 Cyclobacteriaceae bacterium
 205_E2-BRE-15 Cyclobacteriaceae bacterium
 206_G1-WW12-29 Cyclobacteriaceae bacterium
100
100
 207_D1-CHI-15 Cyclobacteriaceae bacterium
 208_E1-EBD-13 Cyclobacteriaceae bacterium
 209_D1-CHI-02 Ekhidna sp.
100
100
 210_B1-GNL1-04 Ekhidna sp.
 211_E2-BRE-04 Ekhidna sp.
100
 212_F3-SA18-02 Cyclobacteriaceae bacterium
 213_B1-GNL1-19 Imperialibacter sp.
43
 214_G1-WW12-35 Imperialibacter sp.
 215_E2-BRE-17 Imperialibacter sp.
100
100
 216_F4-SAH-14 Imperialibacter sp.
 217_E1-EBD-19 Imperialibacter sp.
O025
 218_F3-SA18-06 Imperialibacter sp.
 219_G1-WW12-36 Cyclobacteriaceae bacterium
100
 220_F3-SA18-15 Cyclobacteriaceae bacterium
68
Bacteroiota
 221_F4-SAH-13 Cyclobacteriaceae bacterium
 222_F3-SA18-10 Cyclobacteriaceae bacterium
 223_A1-SPW-02 Cytophagales bacterium
 224_G1-WW12-38 Cytophagales bacterium
100
100
 225_D1-CHI-05 Cytophagales bacterium
 226_E2-BRE-02 Cytophagales bacterium
100
 227_B1-GNL1-11 Cytophagales bacterium
100
100
 228_F4-SAH-11 Cytophagales bacterium
 229_F2-STO-12 Cytophagales bacterium
 230_F1-TOW-10 Cyclobacteriaceae bacterium
100
100
 231_A1-SPW-14 Cyclobacteriaceae bacterium
100
 232_A1-SPW-26 Cyclobacteriaceae bacterium
 233_A1-SPW-35 Fulvivirga sp.
100
 234_E2-BRE-16 Fulvivirga sp.
69
 235_D1-CHI-31 Fulvivirga sp.
 236_G2-EDA-10 Fulvivirga sp.
100
 237_E1-EBD-03 Fulvivirga sp.
100
 238_F2-STO-13 Fulvivirga sp.
 239_C1-SOL-13 Fulvivirga sp.
100
 240_F4-SAH-22 Cyclobacteriaceae bacterium
 241_D1-CHI-04 Vicingaceae bacterium
100
100
 242_F4-SAH-09 Vicingaceae bacterium
 243_B1-GNL1-06 Vicingaceae bacterium
100
 244_C2-GNP5-19 Vicingaceae bacterium
87
90
 245_A1-SPW-21 Vicingaceae bacterium
100
 246_G1-WW12-37 Vicingaceae bacterium
100
O026
 247_E2-BRE-20 Cryomorphaceae bacterium
100
 248_F3-SA18-03 Owenweeksia sp.
 249_E1-EBD-26 Arenibacter algicola
100
100
 250_A1-SPW-32 Arenibacter sp.
100
 251_D1-CHI-23 Muricauda sp.
100
 252_F1-TOW-07 Muricauda sp.
O027
 253_G1-WW12-15 Phaeodactylibacter sp.
Balneolota
 254_F4-SAH-04 Balneola sp.
100
 255_D1-CHI-07 Balneola sp.
100
 256_C1-SOL-04 Balneola sp.
 257_B1-GNL1-08 Balneola sp.
100
 258_G2-EDA-06 Balneola sp.
100
 259_F2-STO-02 Balneola sp.
O028
 260_F3-SA18-05 Balneola sp.
100
 261_C2-GNP5-12 Balneola sp.
100
 262_F3-SA18-04 Balneolaceae bacterium
Rhodo-
therhm-
ota
100
100
 263_F3-SA18-16 Balneolaceae bacterium
100
 264_C1-SOL-02 Gracilimonas sp.
100
100
 265_E1-EBD-01 Gracilimonas sp.
O029
 266_C2-GNP5-09 Rhodothermales bacterium
 267_F4-SAH-23 Longimicrobiales bacterium
100
O030
 268_A1-SPW-16 Longimicrobiales bacterium
Gemma-
timona-
dota
 269_G1-WW12-21 Gimesia sp.
100
100
 270_C2-GNP5-26 Gimesia chilikensis
O031
100
 271_D1-CHI-24 Gimesia maris
 272_F4-SAH-21 Fuerstia sp.
100
 273_G1-WW12-08 Pirellulaceae bacterium
 274_G1-WW12-14 Pirellulales bacterium
100
100
 275_F4-SAH-29 Pirellulales bacterium
O032
Planctomycetota
 276_A1-SPW-29 Lacipirellulaceae bacterium
100
42
100
100
 277_D1-CHI-18 Lacipirellulaceae bacterium
 278_G1-WW12-12 Botrimarina sp.
 279_G2-EDA-01 Phycisphaerales bacterium
 280_F4-SAH-01 Phycisphaerales bacterium
100
 281_A1-SPW-05 Phycisphaerales bacterium
100
 282_C1-SOL-01 Phycisphaerales bacterium
100
 283_F2-STO-01 Phycisphaerales bacterium
O033
 284_B1-GNL1-02 Phycisphaerales bacterium
100
 285_C2-GNP5-07 Phycisphaerales bacterium
100
 286_G1-WW12-04 Phycisphaerales bacterium
100
 287_G1-WW12-06 Phycisphaerales bacterium
100
100
 288_F2-STO-05 Phycisphaerales bacterium
100
 289_F1-TOW-01 Phycisphaerales bacterium
 290_F3-SA18-11 Phycisphaeraceae bacterium
100
Spiro-
chae-
tota
65
 291_F4-SAH-10 Phycisphaeraceae bacterium
O034
 292_G1-WW12-33 Planctomycetota bacterium
 293_F3-SA18-20 Leptospirales bacterium
100
O035
 294_A1-SPW-03 Leptospiraceae bacterium
 295_F4-SAH-19 Marinisubtilis sp.
100
Actino-
mycetota
 296_B1-GNL1-14 Marinisubtilis pacificus
O036
100
100
 297_E1-EBD-24 Marinisubtilis pacificus
 298_C2-GNP5-17 Pontimonas sp.
100
 299_G1-WW12-32 Acidimicrobiales bacterium
100
100
O037
 300_G1-WW12-13 Acidimicrobiales bacterium
100
 301_E1-EBD-21 Ilumatobacter fluminis
 302_C2-GNP5-05 Miltoncostaeaceae bacterium
100
O038
 303_G1-WW12-10 Miltoncostaeaceae bacterium
Chloro-
felx-
ota
100
 304_F4-SAH-27 Miltoncostaeaceae bacterium
 305_C1-SOL-09 Aggregatilineales bacterium
100
O039
 306_G1-WW12-24 Aggregatilineales bacterium
 307_A1-SPW-01 Coleofasciculus sp.
 308_D1-CHI-01 Coleofasciculus sp.
 309_B1-GNL1-01 Coleofasciculus sp.
 310_C1-SOL-03 Coleofasciculus sp.
 311_C2-GNP5-27 Coleofasciculus sp.
 312_E1-EBD-02 Coleofasciculus sp.
 313_E2-BRE-01 Coleofasciculus sp.
 314_F1-TOW-03 Coleofasciculus chthonoplastes
 315_F2-STO-03 Coleofasciculus chthonoplastes
 316_F3-SA18-01 Coleofasciculus chthonoplastes
 317_F4-SAH-05 Coleofasciculus chthonoplastes
 318_G1-WW12-02 Coleofasciculus sp.
 319_G2-EDA-02 Coleofasciculus sp.
 320_G3-WIS-01 Coleofasciculus sp.
49
Cyano-
bacteriota
100
100
0.20
100
Figure S1D
Order level: 39 different associated bacterial orders in the cyanosphere of Coleofasciculus

## Slide 5
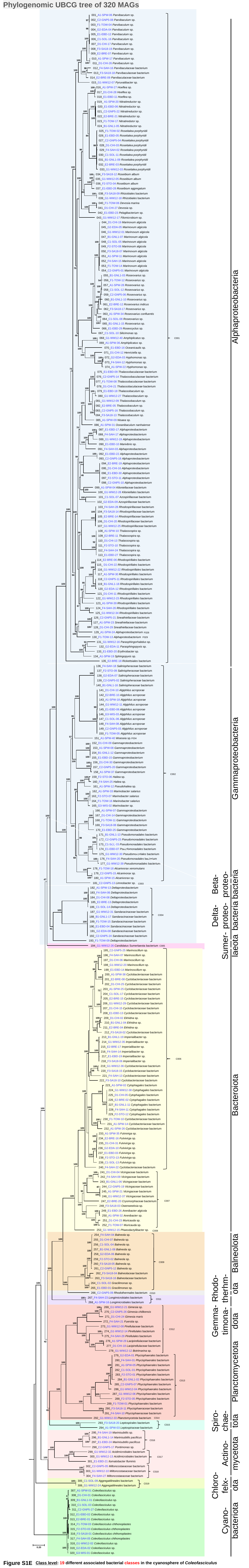

Phylogenomic UBCG tree of 320 MAGs
 001_A1-SPW-06 Parvibaculum sp.
 002_C2-GNP5-08 Parvibaculum sp.
97
 003_F1-TOW-04 Parvibaculum sp.
96
 004_G2-EDA-04 Parvibaculum sp.
 005_E1-EBD-12 Parvibaculum sp.
100
 006_C1-SOL-16 Parvibaculum sp.
 007_D1-CHI-17 Parvibaculum sp.
 008_F3-SA18-19 Parvibaculum sp.
100
 009_E2-BRE-07 Parvibaculum sp.
 010_A1-SPW-17 Parvibaculum sp.
100
100
 011_D1-CHI-26 Parvibaculum sp.
 012_F4-SAH-16 Parvibaculaceae bacterium
98
100
100
 013_F3-SA18-18 Parvibaculaceae bacterium
 014_E2-BRE-09 Parvibaculaceae bacterium
 015_G1-WW12-07 Pyruvatibacter sp.
016_A1-SPW-27 Hoeflea sp.
100
100
 017_D1-CHI-28 Hoeflea sp.
 018_E1-EBD-11 Hoeflea sp.
019_ A1-SPW-20 Nitratireductor sp.
 020_E1-EBD-06 Nitratireductor sp.
100
 021_C2-GNP5-22 Nitratireductor sp.
100
 022_E2-BRE-21 Nitratireductor sp.
100
 023_F1-TOW-17 Nitratiredutor sp.
 024_B1-GNL1-05 Nitratireductor sp.
 025_F1-TOW-02 Roseitalea porphyridii
 026_E1-EBD-05 Roseitalea porphyridii
84
 027_C2-GNP5-04 Roseitalea porphyridii
 028_D1-CHI-03 Roseitalea porphyridii
99
96
 029_F4-SAH-02 Roseitalea porphyridii
 030_C1-SOL-11 Roseitalea porphyridii
100
 031_B1-GNL1-09 Roseitalea porphyridii
 032_E2-BRE-03 Roseitalea porphyridii
100
 033_G1-WW12-03 Roseitalea porphyridii
 034_F3-SA18-12 Roseibium album
100
100
100
 035_G1-WW12-05 Roseibium album
100
 036_F2-STO-04 Roseibium album
 037_E1-EBD-28 Roseibium aggregatum
89
 038_F3-SA18-09 Rhizobiales bacterium
100
 039_G1-WW12-18 Rhizobiales bacterium
 040_F1-TOW-06 Devosia marina
100
100
 041_D1-CHI-27 Devosia sp.
 042_E1-EBD-23 Pelagibacterium sp.
 043_G1-WW12-17 Filomicrobium sp.
 044_D1-CHI-19 Marinovum algicola
 045_G2-EDA-05 Marinovum algicola
 046_G1-WW12-01 Marinovum algicola
 047_B1-GNL1-07 Marinovum algicola
 048_C1-SOL-05 Marinovum algicola
100
 049_F2-STO-08 Marinovum algicola
 050_F3-SA18-07 Marinovum algicola
100
 051_A1-SPW-11 Marinovum algicola
 052_F4-SAH-15 Marinovum algicola
 053_F1-TOW-14 Marinovum algicola
79
Alphaproteobacteria
 054_C2-GNP5-01 Marinovum algicola
 055_B1-GNL1-03 Roseovarius sp.
 056_F1-TOW-12 Roseovarius sp.
54
100
 057_A1-SPW-28 Roseovarius sp.
100
 058_C1-SOL-12 Roseovarius sp.
93
 059_C2-GNP5-06 Roseovarius sp.
100
 060_B1-GNL1-10 Roseovarius sp.
100
 061_E2-BRE-12 Roseovarius indicus
100
 062_F3-SA18-17 Roseovarius sp.
 063_A1-SPW-34 Roseovarius confluentis
100
100
 064_C1-SOL-08 Roseovarius sp.
100
 065_B1-GNL1-15 Roseovarius sp.
 066_E1-EBD-29 Roseocyclus sp.
 067_C1-SOL-10 Silicimonas sp.
48
C001
 068_G1-WW12-40 Amphiplicatus sp.
100
 069_A1-SPW-36 Amphiplicatus sp.
 070_E1-EBD-16 Oceanicaulis sp.
100
 071_D1-CHI-12 Henriciella sp.
100
100
 072_G2-EDA-03 Hyphomonas sp.
100
100
 073_F4-SAH-12 Hyphomonas sp.
 074_A1-SPW-22 Hyphomonas sp.
73
 075_E1-EBD-09 Thalassobaculaceae bacterium
 076_C2-GNP5-14 Thalassobaculaceae bacterium
100
077_F1-TOW-08 Thalassobaculaceae bacterium
 078_D1-CHI-21 Thalassobaculaceae bacterium
100
 079_E1-EBD-18 Thalassobaculum sp.
100
 080_G1-WW12-27 Thalassobaculum sp.
 081_G1-WW12-09 Thalassobaculum sp.
100
100
 082_E2-BRE-05 Thalassobaculum sp.
 083_C2-GNP5-16 Thalassobaculum sp.
100
100
 084_F3-SA18-13 Thalassobaculum sp.
 085_A1-SPW-09 Nisaea sp.
 086_A1-SPW-31 Oceanibaculum nanhaiense
70
 087_E1-EBD-17 Alphaproteobacterium
100
100
 088_F4-SAH-17 Alphaproteobacterium
100
 089_G1-WW12-19 Alphaproteobacterium
100
 090_E1-EBD-10 Marivibrio sp.
 091_F4-SAH-03 Alphaproteobacterium
 092_E1-EBD-22 Alphaproteobacterium
100
94
 093_C2-GNP5-18 Alphaproteobacterium
100
 094_E2-BRE-19 Alphaproteobacterium
 095_D1-CHI-16 Alphaproteobacterium
100
 096_E1-EBD-30 Alphaproteobacterium
46
 097_F2-STO-11 Alphaproteobacterium
 098_C2-GNP5-10 Alphaproteobacterium
 099_A1-SPW-04 Kiloniellaceae bacterium
100
 100_G1-WW12-28 Kiloniellales bacterium
 101_C1-SOL-07 Azospirillaceae bacterium
100
 102_G2-EDA-09 Azospirillaceae bacterium
 103_F4-SAH-28 Rhodospirillaceae bacterium
100
 104_F3-SA18-14 Rhodospirillaceae bacterium
100
100
 105_E2-BRE-14 Rhodospirillaceae bacterium
100
 106_D1-CHI-20 Rhodospirillaceae bacterium
 107_G1-WW12-25 Rhodospirillaceae bacterium
 108_A1-SPW-10 Thalassospira sp.
96
 109_E2-BRE-11 Thalassospira sp.
100
 110_D1-CHI-13 Thalassospira sp.
52
 111_F2-STO-10 Thalassospira sp.
100
 112_F4-SAH-24 Thalassospira sp.
 113_E1-EBD-27 Thalassospira sp.
 114_E2-BRE-06 Rhodospirillales bacterium
100
115_ D1-CHI-22 Rhodospirillales bacterium
100
100
 116_G1-WW12-22 Rhodospirillales bacterium
 117_A1-SPW-30 Rhodospirillales bacterium
100
94
 118_C2-GNP5-11 Rhodospirillales bacterium
95
 119_B1-GNL1-18 Rhodospirillales bacterium
100
100
 120_G2-EDA-12 Rhodospirillales bacterium
99
 121_D1-CHI-11 Rhodospirillales bacterium
 122_G1-WW12-23 Rhodospirillales bacterium
 123_A1-SPW-39 Rhodospirillales bacterium
67
 124_F4-SAH-26 Rhodospirillales bacterium
100
 125_G1-WW12-34 Rhodospirillales bacterium
 126_C2-GNP5-21 Sneathiellaceae bacterium
100
 127_A1-SPW-23 Sneathiellaceae bacterium
51
100
 128_D1-CHI-29 Sneathiellaceae bacterium
F028
 129_A1-SPW-24 Alphaproteobacterium
100
F029
 130_F1-TOW-13 Alphaproteobacterium
 131_G1-WW12-16 Parasphingorhabdus sp.
53
53
 132_G2-EDA-11 Parasphingopyxis sp.
100
 133_E1-EBD-20 Erythrobacter sp.
 134_A1-SPW-19 Sphingopyxis sp.
 135_E2-BRE-18 Rickettsiales bacterium
 136_F4-SAH-18 Salinispheraceae bacterium
 137_F2-STO-09 Salinispheraceae bacterium
100
 138_G2-EDA-07 Salinispheraceae bacterium
 139_C2-GNP5-02 Salinispheraceae bacterium
 140_B1-GNL1-16 Salinispheraceae bacterium
 141_D1-CHI-10 Algiphilus acroporae
 142_E2-BRE-10 Algiphilus acroporae
100
 143_A1-SPW-18 Algiphilus acroporae
 144_G1-WW12-11 Algiphilus acroporae
100
99
 145_E1-EBD-08 Algiphilus acroporae
 146_G3-WIS-03 Algiphilus acroporae
95
Gammaproteobacteria
 147_C1-SOL-06 Algiphilus acroporae
100
 148_F4-SAH-08 Algiphilus acroporae
 149_C2-GNP5-03 Algiphilus acroporae
 150_F1-TOW-05 Algiphilus acroporae
F034
57
 151_A1-SPW-40 Woeseia sp.
 152_D1-CHI-09 Gammaproteobacterium
100
 153_A1-SPW-08 Gammaproteobacterium
100
 154_B1-GNL1-12 Gammaproteobacterium
100
 155_E1-EBD-15 Gammaproteobacterium
100
 156_D1-CHI-30 Gammaproteobacterium
100
100
 157_C2-GNP5-20 Gammaproteobacterium
 158_A1-SPW-37 Gammaproteobacterium
C002
 159_F2-STO-06 Haliea sp.
100
100
100
 160_F4-SAH-25 Haliea sp.
 161_A1-SPW-12 Pseudohaliea sp.
98
 162_A1-SPW-33 Marinobacter salarius
100
 163_F2-STO-07 Marinobacter salarius
100
 164_F1-TOW-16 Marinobacter salarius
 165_G3-WIS-02 Marinobacter sp.
 166_A1-SPW-07 Gammaproteobacterium
 167_D1-CHI-14 Gammaproteobacterium
100
100
 168_F1-TOW-11 Gammaproteobacterium
100
 169_F3-SA18-08 Gammaproteobacterium
100
 170_E1-EBD-25 Gammaproteobacterium
100
 171_B1-GNL1-13 Pseudomonadales bacterium
100
99
 172_C2-GNP5-23 Pseudomonadales bacterium
100
 173_C1-SOL-15 Pseudomonadales bacterium
100
 174_E1-EBD-07 Pseudomonadales bacterium
100
 175_G1-WW12-30 Pseudomonadales bacterium
68
 176_F4-SAH-20 Pseudomonadales bacterium
100
 177_G1-WW12-39 Pseudomonadales bacterium
 178_F1-TOW-18 Alcanivorax xenomutans
Beta-
proteo-
bacteria
100
 179_C2-GNP5-15 Alcanivorax sp.
100
 180_A1-SPW-15 Alcanivorax sp.
47
C003
 181_C2-GNP5-13 Limnobacter sp.
 182_A1-SPW-13 Deltaproteobacterium
 183_F4-SAH-06 Deltaproteobacterium
100
 184_D1-CHI-08 Deltaproteobacterium
100
Delta-
proteo-
bacteria
 185_E2-BRE-13 Deltaproteobacterium
 186_C1-SOL-14 Deltaproteobacterium
100
 187_G1-WW12-31 Sandaracinaceae bacterium
C004
 188_B1-GNL1-17 Sandaracinaceae bacterium
 189_F1-TOW-15 Sandaracinaceae bacterium
100
 190_E1-EBD-04 Sandaracinaceae bacterium
91
 191_G2-EDA-08 Sandaracinaceae bacterium
Sumer-
laeota
 192_C2-GNP5-24 Sandaracinaceae bacterium
 193_F1-TOW-09 Deltaproteobacterium
C005
 194_G1-WW12-26 Candidatus Sumerlaeota bacterium
 195_C2-GNP5-25 Marinoscillum sp.
 196_F4-SAH-07 Marinoscillum sp.
100
 197_D1-CHI-06 Marinoscillum sp.
100
 198_G1-WW12-20 Marinoscillum sp.
99
 199_E1-EBD-14 Marinoscillum sp.
 200_A1-SPW-38 Cyclobacteriaceae bacterium
100
 201_E2-BRE-08 Cyclobacteriaceae bacterium
 202_D1-CHI-25 Cyclobacteriaceae bacterium
100
 203_A1-SPW-25 Cyclobacteriaceae bacterium
73
 204_C1-SOL-17 Cyclobacteriaceae bacterium
 205_E2-BRE-15 Cyclobacteriaceae bacterium
 206_G1-WW12-29 Cyclobacteriaceae bacterium
100
100
 207_D1-CHI-15 Cyclobacteriaceae bacterium
 208_E1-EBD-13 Cyclobacteriaceae bacterium
 209_D1-CHI-02 Ekhidna sp.
100
100
 210_B1-GNL1-04 Ekhidna sp.
 211_E2-BRE-04 Ekhidna sp.
100
 212_F3-SA18-02 Cyclobacteriaceae bacterium
 213_B1-GNL1-19 Imperialibacter sp.
43
 214_G1-WW12-35 Imperialibacter sp.
 215_E2-BRE-17 Imperialibacter sp.
100
100
 216_F4-SAH-14 Imperialibacter sp.
 217_E1-EBD-19 Imperialibacter sp.
C006
 218_F3-SA18-06 Imperialibacter sp.
 219_G1-WW12-36 Cyclobacteriaceae bacterium
100
 220_F3-SA18-15 Cyclobacteriaceae bacterium
68
Bacteroiota
 221_F4-SAH-13 Cyclobacteriaceae bacterium
 222_F3-SA18-10 Cyclobacteriaceae bacterium
 223_A1-SPW-02 Cytophagales bacterium
 224_G1-WW12-38 Cytophagales bacterium
100
100
 225_D1-CHI-05 Cytophagales bacterium
 226_E2-BRE-02 Cytophagales bacterium
100
 227_B1-GNL1-11 Cytophagales bacterium
100
100
 228_F4-SAH-11 Cytophagales bacterium
 229_F2-STO-12 Cytophagales bacterium
 230_F1-TOW-10 Cyclobacteriaceae bacterium
100
100
 231_A1-SPW-14 Cyclobacteriaceae bacterium
100
 232_A1-SPW-26 Cyclobacteriaceae bacterium
 233_A1-SPW-35 Fulvivirga sp.
100
 234_E2-BRE-16 Fulvivirga sp.
69
 235_D1-CHI-31 Fulvivirga sp.
 236_G2-EDA-10 Fulvivirga sp.
100
 237_E1-EBD-03 Fulvivirga sp.
100
 238_F2-STO-13 Fulvivirga sp.
 239_C1-SOL-13 Fulvivirga sp.
100
 240_F4-SAH-22 Cyclobacteriaceae bacterium
 241_D1-CHI-04 Vicingaceae bacterium
100
100
 242_F4-SAH-09 Vicingaceae bacterium
 243_B1-GNL1-06 Vicingaceae bacterium
100
 244_C2-GNP5-19 Vicingaceae bacterium
87
90
 245_A1-SPW-21 Vicingaceae bacterium
100
 246_G1-WW12-37 Vicingaceae bacterium
100
C007
 247_E2-BRE-20 Cryomorphaceae bacterium
100
 248_F3-SA18-03 Owenweeksia sp.
 249_E1-EBD-26 Arenibacter algicola
100
100
 250_A1-SPW-32 Arenibacter sp.
100
 251_D1-CHI-23 Muricauda sp.
100
 252_F1-TOW-07 Muricauda sp.
C008
 253_G1-WW12-15 Phaeodactylibacter sp.
Balneolota
 254_F4-SAH-04 Balneola sp.
100
 255_D1-CHI-07 Balneola sp.
100
 256_C1-SOL-04 Balneola sp.
 257_B1-GNL1-08 Balneola sp.
100
 258_G2-EDA-06 Balneola sp.
100
 259_F2-STO-02 Balneola sp.
C009
 260_F3-SA18-05 Balneola sp.
100
 261_C2-GNP5-12 Balneola sp.
100
 262_F3-SA18-04 Balneolaceae bacterium
Rhodo-
therhm-
ota
100
100
 263_F3-SA18-16 Balneolaceae bacterium
100
 264_C1-SOL-02 Gracilimonas sp.
100
100
 265_E1-EBD-01 Gracilimonas sp.
C010
 266_C2-GNP5-09 Rhodothermales bacterium
 267_F4-SAH-23 Longimicrobiales bacterium
C011
100
 268_A1-SPW-16 Longimicrobiales bacterium
Gemma-
timona-
dota
 269_G1-WW12-21 Gimesia sp.
100
100
 270_C2-GNP5-26 Gimesia chilikensis
100
 271_D1-CHI-24 Gimesia maris
 272_F4-SAH-21 Fuerstia sp.
100
 273_G1-WW12-08 Pirellulaceae bacterium
C012
 274_G1-WW12-14 Pirellulales bacterium
100
100
 275_F4-SAH-29 Pirellulales bacterium
Planctomycetota
 276_A1-SPW-29 Lacipirellulaceae bacterium
100
42
100
100
 277_D1-CHI-18 Lacipirellulaceae bacterium
 278_G1-WW12-12 Botrimarina sp.
 279_G2-EDA-01 Phycisphaerales bacterium
 280_F4-SAH-01 Phycisphaerales bacterium
100
 281_A1-SPW-05 Phycisphaerales bacterium
100
 282_C1-SOL-01 Phycisphaerales bacterium
100
 283_F2-STO-01 Phycisphaerales bacterium
C013
 284_B1-GNL1-02 Phycisphaerales bacterium
100
 285_C2-GNP5-07 Phycisphaerales bacterium
100
 286_G1-WW12-04 Phycisphaerales bacterium
100
 287_G1-WW12-06 Phycisphaerales bacterium
100
100
 288_F2-STO-05 Phycisphaerales bacterium
100
 289_F1-TOW-01 Phycisphaerales bacterium
 290_F3-SA18-11 Phycisphaeraceae bacterium
100
Spiro-
chae-
tota
65
 291_F4-SAH-10 Phycisphaeraceae bacterium
C014
 292_G1-WW12-33 Planctomycetota bacterium
 293_F3-SA18-20 Leptospirales bacterium
100
C015
 294_A1-SPW-03 Leptospiraceae bacterium
 295_F4-SAH-19 Marinisubtilis sp.
100
Actino-
mycetota
 296_B1-GNL1-14 Marinisubtilis pacificus
C016
100
100
 297_E1-EBD-24 Marinisubtilis pacificus
 298_C2-GNP5-17 Pontimonas sp.
100
 299_G1-WW12-32 Acidimicrobiales bacterium
100
C017
100
 300_G1-WW12-13 Acidimicrobiales bacterium
100
 301_E1-EBD-21 Ilumatobacter fluminis
 302_C2-GNP5-05 Miltoncostaeaceae bacterium
100
C018
 303_G1-WW12-10 Miltoncostaeaceae bacterium
Chloro-
felx-
ota
100
 304_F4-SAH-27 Miltoncostaeaceae bacterium
 305_C1-SOL-09 Aggregatilineales bacterium
100
C019
 306_G1-WW12-24 Aggregatilineales bacterium
 307_A1-SPW-01 Coleofasciculus sp.
 308_D1-CHI-01 Coleofasciculus sp.
 309_B1-GNL1-01 Coleofasciculus sp.
 310_C1-SOL-03 Coleofasciculus sp.
 311_C2-GNP5-27 Coleofasciculus sp.
 312_E1-EBD-02 Coleofasciculus sp.
 313_E2-BRE-01 Coleofasciculus sp.
 314_F1-TOW-03 Coleofasciculus chthonoplastes
 315_F2-STO-03 Coleofasciculus chthonoplastes
 316_F3-SA18-01 Coleofasciculus chthonoplastes
 317_F4-SAH-05 Coleofasciculus chthonoplastes
 318_G1-WW12-02 Coleofasciculus sp.
 319_G2-EDA-02 Coleofasciculus sp.
 320_G3-WIS-01 Coleofasciculus sp.
49
Cyano-
bacteriota
100
100
0.20
100
Figure S1E
Class level: 19 different associated bacterial classes in the cyanosphere of Coleofasciculus

## Slide 6
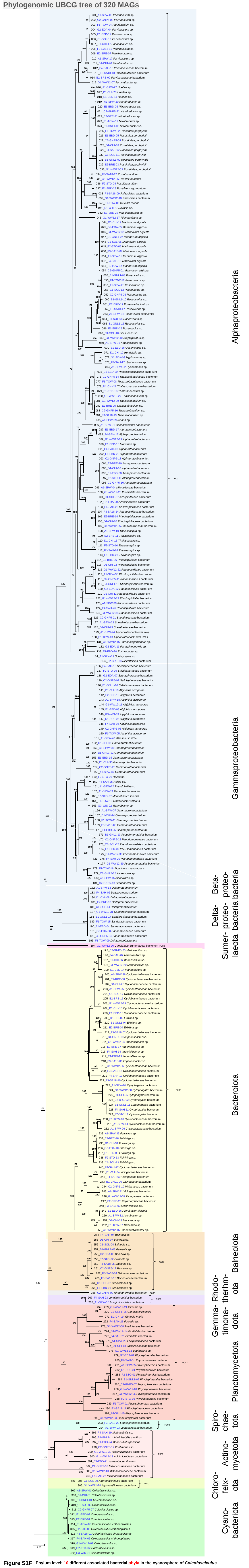

Phylogenomic UBCG tree of 320 MAGs
 001_A1-SPW-06 Parvibaculum sp.
 002_C2-GNP5-08 Parvibaculum sp.
97
 003_F1-TOW-04 Parvibaculum sp.
96
 004_G2-EDA-04 Parvibaculum sp.
 005_E1-EBD-12 Parvibaculum sp.
100
 006_C1-SOL-16 Parvibaculum sp.
 007_D1-CHI-17 Parvibaculum sp.
 008_F3-SA18-19 Parvibaculum sp.
100
 009_E2-BRE-07 Parvibaculum sp.
 010_A1-SPW-17 Parvibaculum sp.
100
100
 011_D1-CHI-26 Parvibaculum sp.
 012_F4-SAH-16 Parvibaculaceae bacterium
98
100
100
 013_F3-SA18-18 Parvibaculaceae bacterium
 014_E2-BRE-09 Parvibaculaceae bacterium
 015_G1-WW12-07 Pyruvatibacter sp.
016_A1-SPW-27 Hoeflea sp.
100
100
 017_D1-CHI-28 Hoeflea sp.
 018_E1-EBD-11 Hoeflea sp.
019_ A1-SPW-20 Nitratireductor sp.
 020_E1-EBD-06 Nitratireductor sp.
100
 021_C2-GNP5-22 Nitratireductor sp.
100
 022_E2-BRE-21 Nitratireductor sp.
100
 023_F1-TOW-17 Nitratiredutor sp.
 024_B1-GNL1-05 Nitratireductor sp.
 025_F1-TOW-02 Roseitalea porphyridii
 026_E1-EBD-05 Roseitalea porphyridii
84
 027_C2-GNP5-04 Roseitalea porphyridii
 028_D1-CHI-03 Roseitalea porphyridii
99
96
 029_F4-SAH-02 Roseitalea porphyridii
 030_C1-SOL-11 Roseitalea porphyridii
100
 031_B1-GNL1-09 Roseitalea porphyridii
 032_E2-BRE-03 Roseitalea porphyridii
100
 033_G1-WW12-03 Roseitalea porphyridii
 034_F3-SA18-12 Roseibium album
100
100
100
 035_G1-WW12-05 Roseibium album
100
 036_F2-STO-04 Roseibium album
 037_E1-EBD-28 Roseibium aggregatum
89
 038_F3-SA18-09 Rhizobiales bacterium
100
 039_G1-WW12-18 Rhizobiales bacterium
 040_F1-TOW-06 Devosia marina
100
100
 041_D1-CHI-27 Devosia sp.
 042_E1-EBD-23 Pelagibacterium sp.
 043_G1-WW12-17 Filomicrobium sp.
 044_D1-CHI-19 Marinovum algicola
 045_G2-EDA-05 Marinovum algicola
 046_G1-WW12-01 Marinovum algicola
 047_B1-GNL1-07 Marinovum algicola
 048_C1-SOL-05 Marinovum algicola
100
 049_F2-STO-08 Marinovum algicola
 050_F3-SA18-07 Marinovum algicola
100
 051_A1-SPW-11 Marinovum algicola
 052_F4-SAH-15 Marinovum algicola
 053_F1-TOW-14 Marinovum algicola
79
Alphaproteobacteria
 054_C2-GNP5-01 Marinovum algicola
 055_B1-GNL1-03 Roseovarius sp.
 056_F1-TOW-12 Roseovarius sp.
54
100
 057_A1-SPW-28 Roseovarius sp.
100
 058_C1-SOL-12 Roseovarius sp.
93
 059_C2-GNP5-06 Roseovarius sp.
100
 060_B1-GNL1-10 Roseovarius sp.
100
 061_E2-BRE-12 Roseovarius indicus
100
 062_F3-SA18-17 Roseovarius sp.
 063_A1-SPW-34 Roseovarius confluentis
100
100
 064_C1-SOL-08 Roseovarius sp.
100
 065_B1-GNL1-15 Roseovarius sp.
 066_E1-EBD-29 Roseocyclus sp.
 067_C1-SOL-10 Silicimonas sp.
48
 068_G1-WW12-40 Amphiplicatus sp.
100
 069_A1-SPW-36 Amphiplicatus sp.
 070_E1-EBD-16 Oceanicaulis sp.
100
 071_D1-CHI-12 Henriciella sp.
100
100
 072_G2-EDA-03 Hyphomonas sp.
100
100
 073_F4-SAH-12 Hyphomonas sp.
 074_A1-SPW-22 Hyphomonas sp.
73
 075_E1-EBD-09 Thalassobaculaceae bacterium
 076_C2-GNP5-14 Thalassobaculaceae bacterium
100
077_F1-TOW-08 Thalassobaculaceae bacterium
 078_D1-CHI-21 Thalassobaculaceae bacterium
100
 079_E1-EBD-18 Thalassobaculum sp.
100
 080_G1-WW12-27 Thalassobaculum sp.
 081_G1-WW12-09 Thalassobaculum sp.
100
100
 082_E2-BRE-05 Thalassobaculum sp.
 083_C2-GNP5-16 Thalassobaculum sp.
100
100
 084_F3-SA18-13 Thalassobaculum sp.
 085_A1-SPW-09 Nisaea sp.
 086_A1-SPW-31 Oceanibaculum nanhaiense
70
 087_E1-EBD-17 Alphaproteobacterium
100
100
 088_F4-SAH-17 Alphaproteobacterium
100
 089_G1-WW12-19 Alphaproteobacterium
100
 090_E1-EBD-10 Marivibrio sp.
 091_F4-SAH-03 Alphaproteobacterium
 092_E1-EBD-22 Alphaproteobacterium
100
94
 093_C2-GNP5-18 Alphaproteobacterium
100
 094_E2-BRE-19 Alphaproteobacterium
 095_D1-CHI-16 Alphaproteobacterium
100
 096_E1-EBD-30 Alphaproteobacterium
46
P001
 097_F2-STO-11 Alphaproteobacterium
 098_C2-GNP5-10 Alphaproteobacterium
 099_A1-SPW-04 Kiloniellaceae bacterium
100
 100_G1-WW12-28 Kiloniellales bacterium
 101_C1-SOL-07 Azospirillaceae bacterium
100
 102_G2-EDA-09 Azospirillaceae bacterium
 103_F4-SAH-28 Rhodospirillaceae bacterium
100
 104_F3-SA18-14 Rhodospirillaceae bacterium
100
100
 105_E2-BRE-14 Rhodospirillaceae bacterium
100
 106_D1-CHI-20 Rhodospirillaceae bacterium
 107_G1-WW12-25 Rhodospirillaceae bacterium
 108_A1-SPW-10 Thalassospira sp.
96
 109_E2-BRE-11 Thalassospira sp.
100
 110_D1-CHI-13 Thalassospira sp.
52
 111_F2-STO-10 Thalassospira sp.
100
 112_F4-SAH-24 Thalassospira sp.
 113_E1-EBD-27 Thalassospira sp.
 114_E2-BRE-06 Rhodospirillales bacterium
100
115_ D1-CHI-22 Rhodospirillales bacterium
100
100
 116_G1-WW12-22 Rhodospirillales bacterium
 117_A1-SPW-30 Rhodospirillales bacterium
100
94
 118_C2-GNP5-11 Rhodospirillales bacterium
95
 119_B1-GNL1-18 Rhodospirillales bacterium
100
100
 120_G2-EDA-12 Rhodospirillales bacterium
99
 121_D1-CHI-11 Rhodospirillales bacterium
 122_G1-WW12-23 Rhodospirillales bacterium
 123_A1-SPW-39 Rhodospirillales bacterium
67
 124_F4-SAH-26 Rhodospirillales bacterium
100
 125_G1-WW12-34 Rhodospirillales bacterium
 126_C2-GNP5-21 Sneathiellaceae bacterium
100
 127_A1-SPW-23 Sneathiellaceae bacterium
51
100
 128_D1-CHI-29 Sneathiellaceae bacterium
F028
 129_A1-SPW-24 Alphaproteobacterium
100
F029
 130_F1-TOW-13 Alphaproteobacterium
 131_G1-WW12-16 Parasphingorhabdus sp.
53
53
 132_G2-EDA-11 Parasphingopyxis sp.
100
 133_E1-EBD-20 Erythrobacter sp.
 134_A1-SPW-19 Sphingopyxis sp.
 135_E2-BRE-18 Rickettsiales bacterium
 136_F4-SAH-18 Salinispheraceae bacterium
 137_F2-STO-09 Salinispheraceae bacterium
100
 138_G2-EDA-07 Salinispheraceae bacterium
 139_C2-GNP5-02 Salinispheraceae bacterium
 140_B1-GNL1-16 Salinispheraceae bacterium
 141_D1-CHI-10 Algiphilus acroporae
 142_E2-BRE-10 Algiphilus acroporae
100
 143_A1-SPW-18 Algiphilus acroporae
 144_G1-WW12-11 Algiphilus acroporae
100
99
 145_E1-EBD-08 Algiphilus acroporae
 146_G3-WIS-03 Algiphilus acroporae
95
Gammaproteobacteria
 147_C1-SOL-06 Algiphilus acroporae
100
 148_F4-SAH-08 Algiphilus acroporae
 149_C2-GNP5-03 Algiphilus acroporae
 150_F1-TOW-05 Algiphilus acroporae
F034
57
 151_A1-SPW-40 Woeseia sp.
 152_D1-CHI-09 Gammaproteobacterium
100
 153_A1-SPW-08 Gammaproteobacterium
100
 154_B1-GNL1-12 Gammaproteobacterium
100
 155_E1-EBD-15 Gammaproteobacterium
100
 156_D1-CHI-30 Gammaproteobacterium
100
100
 157_C2-GNP5-20 Gammaproteobacterium
 158_A1-SPW-37 Gammaproteobacterium
 159_F2-STO-06 Haliea sp.
100
100
100
 160_F4-SAH-25 Haliea sp.
 161_A1-SPW-12 Pseudohaliea sp.
98
 162_A1-SPW-33 Marinobacter salarius
100
 163_F2-STO-07 Marinobacter salarius
100
 164_F1-TOW-16 Marinobacter salarius
 165_G3-WIS-02 Marinobacter sp.
 166_A1-SPW-07 Gammaproteobacterium
 167_D1-CHI-14 Gammaproteobacterium
100
100
 168_F1-TOW-11 Gammaproteobacterium
100
 169_F3-SA18-08 Gammaproteobacterium
100
 170_E1-EBD-25 Gammaproteobacterium
100
 171_B1-GNL1-13 Pseudomonadales bacterium
100
99
 172_C2-GNP5-23 Pseudomonadales bacterium
100
 173_C1-SOL-15 Pseudomonadales bacterium
100
 174_E1-EBD-07 Pseudomonadales bacterium
100
 175_G1-WW12-30 Pseudomonadales bacterium
68
 176_F4-SAH-20 Pseudomonadales bacterium
100
 177_G1-WW12-39 Pseudomonadales bacterium
 178_F1-TOW-18 Alcanivorax xenomutans
Beta-
proteo-
bacteria
100
 179_C2-GNP5-15 Alcanivorax sp.
100
 180_A1-SPW-15 Alcanivorax sp.
47
 181_C2-GNP5-13 Limnobacter sp.
 182_A1-SPW-13 Deltaproteobacterium
 183_F4-SAH-06 Deltaproteobacterium
100
 184_D1-CHI-08 Deltaproteobacterium
100
Delta-
proteo-
bacteria
 185_E2-BRE-13 Deltaproteobacterium
 186_C1-SOL-14 Deltaproteobacterium
100
 187_G1-WW12-31 Sandaracinaceae bacterium
 188_B1-GNL1-17 Sandaracinaceae bacterium
 189_F1-TOW-15 Sandaracinaceae bacterium
100
 190_E1-EBD-04 Sandaracinaceae bacterium
91
 191_G2-EDA-08 Sandaracinaceae bacterium
Sumer-
laeota
 192_C2-GNP5-24 Sandaracinaceae bacterium
 193_F1-TOW-09 Deltaproteobacterium
P002
 194_G1-WW12-26 Candidatus Sumerlaeota bacterium
 195_C2-GNP5-25 Marinoscillum sp.
 196_F4-SAH-07 Marinoscillum sp.
100
 197_D1-CHI-06 Marinoscillum sp.
100
 198_G1-WW12-20 Marinoscillum sp.
99
 199_E1-EBD-14 Marinoscillum sp.
 200_A1-SPW-38 Cyclobacteriaceae bacterium
100
 201_E2-BRE-08 Cyclobacteriaceae bacterium
 202_D1-CHI-25 Cyclobacteriaceae bacterium
100
 203_A1-SPW-25 Cyclobacteriaceae bacterium
73
 204_C1-SOL-17 Cyclobacteriaceae bacterium
 205_E2-BRE-15 Cyclobacteriaceae bacterium
 206_G1-WW12-29 Cyclobacteriaceae bacterium
100
100
 207_D1-CHI-15 Cyclobacteriaceae bacterium
 208_E1-EBD-13 Cyclobacteriaceae bacterium
 209_D1-CHI-02 Ekhidna sp.
100
100
 210_B1-GNL1-04 Ekhidna sp.
 211_E2-BRE-04 Ekhidna sp.
100
 212_F3-SA18-02 Cyclobacteriaceae bacterium
 213_B1-GNL1-19 Imperialibacter sp.
43
 214_G1-WW12-35 Imperialibacter sp.
 215_E2-BRE-17 Imperialibacter sp.
100
100
 216_F4-SAH-14 Imperialibacter sp.
 217_E1-EBD-19 Imperialibacter sp.
 218_F3-SA18-06 Imperialibacter sp.
 219_G1-WW12-36 Cyclobacteriaceae bacterium
100
 220_F3-SA18-15 Cyclobacteriaceae bacterium
68
Bacteroiota
 221_F4-SAH-13 Cyclobacteriaceae bacterium
 222_F3-SA18-10 Cyclobacteriaceae bacterium
 223_A1-SPW-02 Cytophagales bacterium
P003
 224_G1-WW12-38 Cytophagales bacterium
100
100
 225_D1-CHI-05 Cytophagales bacterium
 226_E2-BRE-02 Cytophagales bacterium
100
 227_B1-GNL1-11 Cytophagales bacterium
100
100
 228_F4-SAH-11 Cytophagales bacterium
 229_F2-STO-12 Cytophagales bacterium
 230_F1-TOW-10 Cyclobacteriaceae bacterium
100
100
 231_A1-SPW-14 Cyclobacteriaceae bacterium
100
 232_A1-SPW-26 Cyclobacteriaceae bacterium
 233_A1-SPW-35 Fulvivirga sp.
100
 234_E2-BRE-16 Fulvivirga sp.
69
 235_D1-CHI-31 Fulvivirga sp.
 236_G2-EDA-10 Fulvivirga sp.
100
 237_E1-EBD-03 Fulvivirga sp.
100
 238_F2-STO-13 Fulvivirga sp.
 239_C1-SOL-13 Fulvivirga sp.
100
 240_F4-SAH-22 Cyclobacteriaceae bacterium
 241_D1-CHI-04 Vicingaceae bacterium
100
100
 242_F4-SAH-09 Vicingaceae bacterium
 243_B1-GNL1-06 Vicingaceae bacterium
100
 244_C2-GNP5-19 Vicingaceae bacterium
87
90
 245_A1-SPW-21 Vicingaceae bacterium
100
 246_G1-WW12-37 Vicingaceae bacterium
100
 247_E2-BRE-20 Cryomorphaceae bacterium
100
 248_F3-SA18-03 Owenweeksia sp.
 249_E1-EBD-26 Arenibacter algicola
100
100
 250_A1-SPW-32 Arenibacter sp.
100
 251_D1-CHI-23 Muricauda sp.
100
 252_F1-TOW-07 Muricauda sp.
 253_G1-WW12-15 Phaeodactylibacter sp.
Balneolota
 254_F4-SAH-04 Balneola sp.
100
 255_D1-CHI-07 Balneola sp.
100
 256_C1-SOL-04 Balneola sp.
 257_B1-GNL1-08 Balneola sp.
100
 258_G2-EDA-06 Balneola sp.
100
 259_F2-STO-02 Balneola sp.
P004
 260_F3-SA18-05 Balneola sp.
100
 261_C2-GNP5-12 Balneola sp.
100
 262_F3-SA18-04 Balneolaceae bacterium
Rhodo-
therhm-
ota
100
100
 263_F3-SA18-16 Balneolaceae bacterium
100
 264_C1-SOL-02 Gracilimonas sp.
100
100
 265_E1-EBD-01 Gracilimonas sp.
P005
 266_C2-GNP5-09 Rhodothermales bacterium
 267_F4-SAH-23 Longimicrobiales bacterium
P006
100
 268_A1-SPW-16 Longimicrobiales bacterium
Gemma-
timona-
dota
 269_G1-WW12-21 Gimesia sp.
100
100
 270_C2-GNP5-26 Gimesia chilikensis
100
 271_D1-CHI-24 Gimesia maris
 272_F4-SAH-21 Fuerstia sp.
100
 273_G1-WW12-08 Pirellulaceae bacterium
 274_G1-WW12-14 Pirellulales bacterium
100
100
 275_F4-SAH-29 Pirellulales bacterium
Planctomycetota
 276_A1-SPW-29 Lacipirellulaceae bacterium
100
42
100
100
 277_D1-CHI-18 Lacipirellulaceae bacterium
 278_G1-WW12-12 Botrimarina sp.
 279_G2-EDA-01 Phycisphaerales bacterium
 280_F4-SAH-01 Phycisphaerales bacterium
P007
100
 281_A1-SPW-05 Phycisphaerales bacterium
100
 282_C1-SOL-01 Phycisphaerales bacterium
100
 283_F2-STO-01 Phycisphaerales bacterium
 284_B1-GNL1-02 Phycisphaerales bacterium
100
 285_C2-GNP5-07 Phycisphaerales bacterium
100
 286_G1-WW12-04 Phycisphaerales bacterium
100
 287_G1-WW12-06 Phycisphaerales bacterium
100
100
 288_F2-STO-05 Phycisphaerales bacterium
100
 289_F1-TOW-01 Phycisphaerales bacterium
 290_F3-SA18-11 Phycisphaeraceae bacterium
100
Spiro-
chae-
tota
65
 291_F4-SAH-10 Phycisphaeraceae bacterium
 292_G1-WW12-33 Planctomycetota bacterium
 293_F3-SA18-20 Leptospirales bacterium
100
P008
 294_A1-SPW-03 Leptospiraceae bacterium
 295_F4-SAH-19 Marinisubtilis sp.
100
Actino-
mycetota
 296_B1-GNL1-14 Marinisubtilis pacificus
100
100
 297_E1-EBD-24 Marinisubtilis pacificus
 298_C2-GNP5-17 Pontimonas sp.
100
 299_G1-WW12-32 Acidimicrobiales bacterium
100
P009
100
 300_G1-WW12-13 Acidimicrobiales bacterium
100
 301_E1-EBD-21 Ilumatobacter fluminis
 302_C2-GNP5-05 Miltoncostaeaceae bacterium
100
 303_G1-WW12-10 Miltoncostaeaceae bacterium
Chloro-
felx-
ota
100
 304_F4-SAH-27 Miltoncostaeaceae bacterium
 305_C1-SOL-09 Aggregatilineales bacterium
100
P010
 306_G1-WW12-24 Aggregatilineales bacterium
 307_A1-SPW-01 Coleofasciculus sp.
 308_D1-CHI-01 Coleofasciculus sp.
 309_B1-GNL1-01 Coleofasciculus sp.
 310_C1-SOL-03 Coleofasciculus sp.
 311_C2-GNP5-27 Coleofasciculus sp.
 312_E1-EBD-02 Coleofasciculus sp.
 313_E2-BRE-01 Coleofasciculus sp.
 314_F1-TOW-03 Coleofasciculus chthonoplastes
 315_F2-STO-03 Coleofasciculus chthonoplastes
 316_F3-SA18-01 Coleofasciculus chthonoplastes
 317_F4-SAH-05 Coleofasciculus chthonoplastes
 318_G1-WW12-02 Coleofasciculus sp.
 319_G2-EDA-02 Coleofasciculus sp.
 320_G3-WIS-01 Coleofasciculus sp.
49
Cyano-
bacteriota
100
100
0.20
100
Figure S1F
Phylum level: 10 different associated bacterial phyla in the cyanosphere of Coleofasciculus
