# Supplementary material for: The microbiome of marine mat-forming cyanobacteria—a microcosm of taxonomic novelty and phototrophic diversity
Source: ISME Commun. 2026 Feb 27;6(1):ycag041. doi: 10.1093/ismeco/ycag041 (PMC13043013; doi:10.1093/ismeco/ycag041)
Supplement: Figure-S2_Taxon-Matrix_tSNA_251024_ycag041 [file figure-s2_taxon-matrix_tsna_251024_ycag041.pptx]

## Slide 1
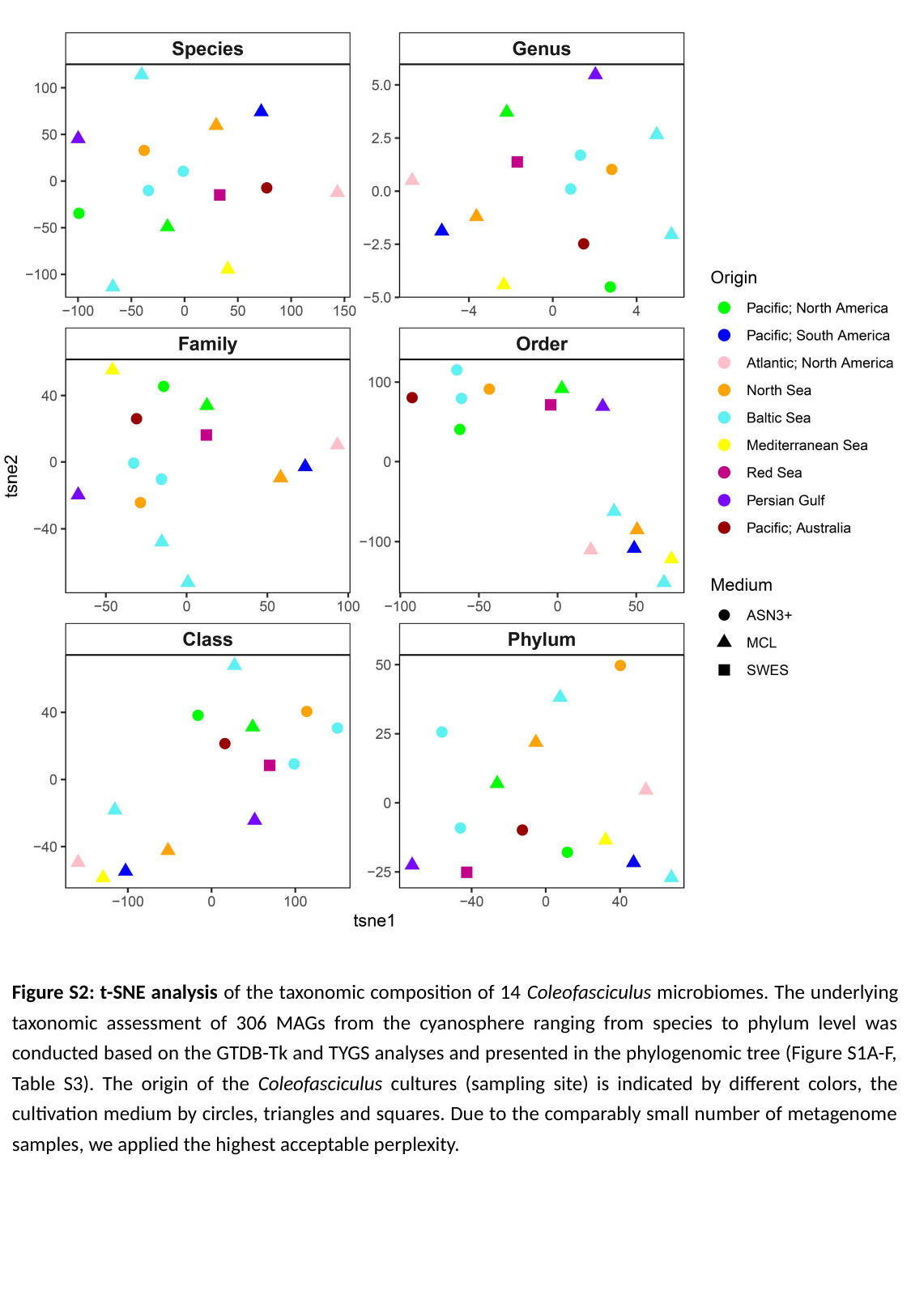

Figure S2: t-SNE analysis of the taxonomic composition of 14 Coleofasciculus microbiomes. The underlying taxonomic assessment of 306 MAGs from the cyanosphere ranging from species to phylum level was conducted based on the GTDB-Tk and TYGS analyses and presented in the phylogenomic tree (Figure S1A-F, Table S3). The origin of the Coleofasciculus cultures (sampling site) is indicated by different colors, the cultivation medium by circles, triangles and squares. Due to the comparably small number of metagenome samples, we applied the highest acceptable perplexity.
