# Supplementary material for: The microbiome of marine mat-forming cyanobacteria—a microcosm of taxonomic novelty and phototrophic diversity
Source: ISME Commun. 2026 Feb 27;6(1):ycag041. doi: 10.1093/ismeco/ycag041 (PMC13043013; doi:10.1093/ismeco/ycag041)
Supplement: Figure-S3-S8_UBCG-Trees_260224_ycag041 [file figure-s3-s8_ubcg-trees_260224_ycag041.pptx]

## Slide 1
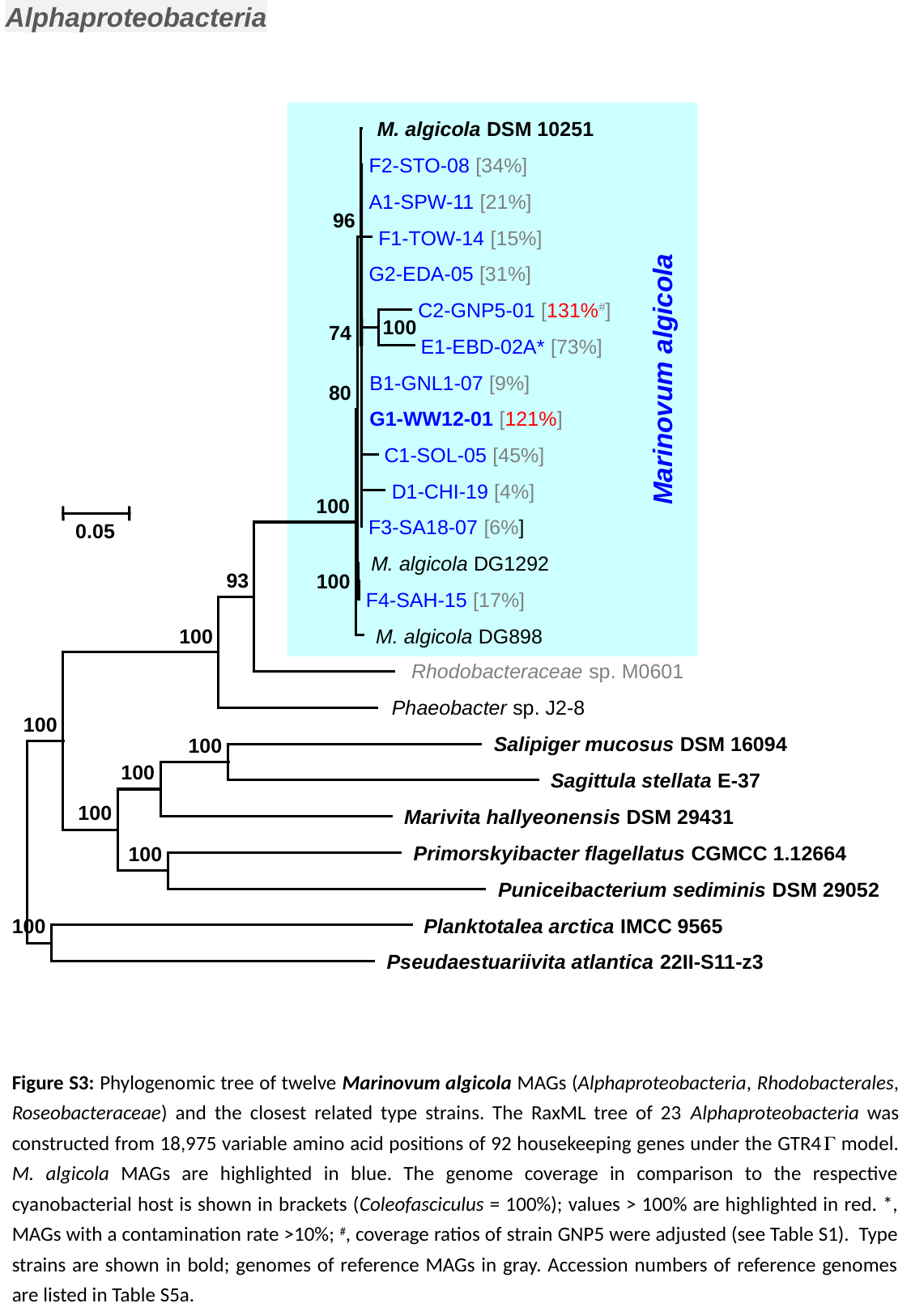

Alphaproteobacteria
 M. algicola DSM 10251
F2-STO-08 [34%]
A1-SPW-11 [21%]
F1-TOW-14 [15%]
G2-EDA-05 [31%]
C2-GNP5-01 [131%#]
E1-EBD-02A* [73%]
B1-GNL1-07 [9%]
G1-WW12-01 [121%]
C1-SOL-05 [45%]
D1-CHI-19 [4%]
F3-SA18-07 [6%]
 M. algicola DG1292
F4-SAH-15 [17%]
 M. algicola DG898
 Rhodobacteraceae sp. M0601
 Phaeobacter sp. J2-8
 Salipiger mucosus DSM 16094
 Sagittula stellata E-37
 Marivita hallyeonensis DSM 29431
 Primorskyibacter flagellatus CGMCC 1.12664
 Puniceibacterium sediminis DSM 29052
 Planktotalea arctica IMCC 9565
 Pseudaestuariivita atlantica 22II-S11-z3
96
100
74
80
100
93
100
100
100
100
100
100
100
100
0.05
Marinovum algicola
Figure S3: Phylogenomic tree of twelve Marinovum algicola MAGs (Alphaproteobacteria, Rhodobacterales, Roseobacteraceae) and the closest related type strains. The RaxML tree of 23 Alphaproteobacteria was constructed from 18,975 variable amino acid positions of 92 housekeeping genes under the GTR4G model. M. algicola MAGs are highlighted in blue. The genome coverage in comparison to the respective cyanobacterial host is shown in brackets (Coleofasciculus = 100%); values > 100% are highlighted in red. *, MAGs with a contamination rate >10%; #, coverage ratios of strain GNP5 were adjusted (see Table S1). Type strains are shown in bold; genomes of reference MAGs in gray. Accession numbers of reference genomes are listed in Table S5a.

## Slide 2
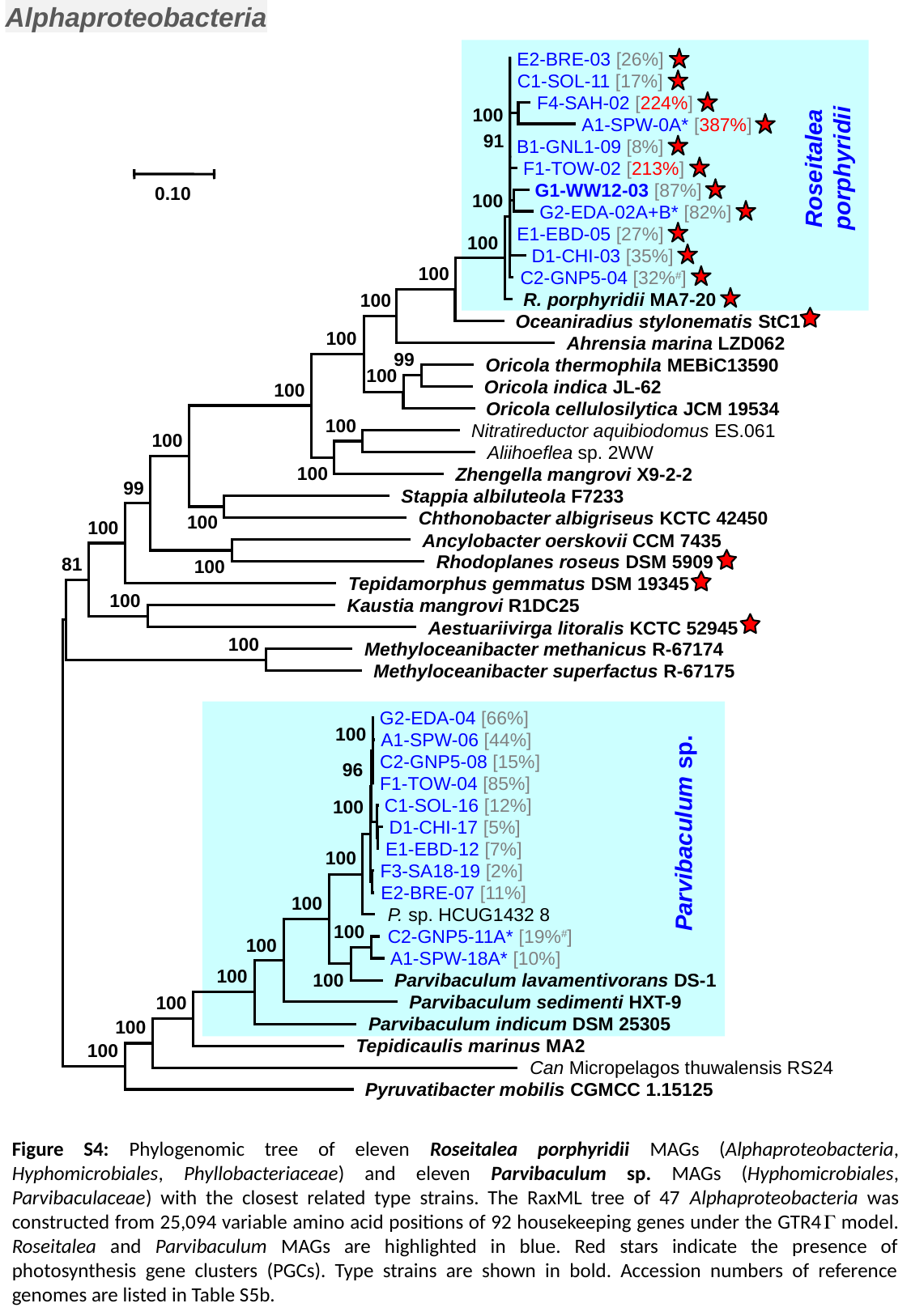

Alphaproteobacteria
E2-BRE-03 [26%]
C1-SOL-11 [17%]
F4-SAH-02 [224%]
A1-SPW-0A* [387%]
B1-GNL1-09 [8%]
F1-TOW-02 [213%]
G1-WW12-03 [87%]
G2-EDA-02A+B* [82%]
E1-EBD-05 [27%]
D1-CHI-03 [35%]
C2-GNP5-04 [32%#]
 R. porphyridii MA7-20
 Oceaniradius stylonematis StC1
 Ahrensia marina LZD062
 Oricola thermophila MEBiC13590
 Oricola indica JL-62
 Oricola cellulosilytica JCM 19534
 Nitratireductor aquibiodomus ES.061
 Aliihoeflea sp. 2WW
 Zhengella mangrovi X9-2-2
 Stappia albiluteola F7233
 Chthonobacter albigriseus KCTC 42450
 Ancylobacter oerskovii CCM 7435
 Rhodoplanes roseus DSM 5909
 Tepidamorphus gemmatus DSM 19345
 Kaustia mangrovi R1DC25
 Aestuariivirga litoralis KCTC 52945
 Methyloceanibacter methanicus R-67174
 Methyloceanibacter superfactus R-67175
G2-EDA-04 [66%]
A1-SPW-06 [44%]
C2-GNP5-08 [15%]
F1-TOW-04 [85%]
C1-SOL-16 [12%]
D1-CHI-17 [5%]
E1-EBD-12 [7%]
F3-SA18-19 [2%]
E2-BRE-07 [11%]
 P. sp. HCUG1432 8
C2-GNP5-11A* [19%#]
A1-SPW-18A* [10%]
 Parvibaculum lavamentivorans DS-1
 Parvibaculum sedimenti HXT-9
 Parvibaculum indicum DSM 25305
 Tepidicaulis marinus MA2
 Can Micropelagos thuwalensis RS24
 Pyruvatibacter mobilis CGMCC 1.15125
100
91
100
100
100
100
100
99
100
100
100
100
100
99
100
100
81
100
100
100
100
96
100
100
100
100
100
100
100
100
100
100
Roseitalea
porphyridii
0.10
Parvibaculum sp.
Figure S4: Phylogenomic tree of eleven Roseitalea porphyridii MAGs (Alphaproteobacteria, Hyphomicrobiales, Phyllobacteriaceae) and eleven Parvibaculum sp. MAGs (Hyphomicrobiales, Parvibaculaceae) with the closest related type strains. The RaxML tree of 47 Alphaproteobacteria was constructed from 25,094 variable amino acid positions of 92 housekeeping genes under the GTR4G model. Roseitalea and Parvibaculum MAGs are highlighted in blue. Red stars indicate the presence of photosynthesis gene clusters (PGCs). Type strains are shown in bold. Accession numbers of reference genomes are listed in Table S5b.

## Slide 3
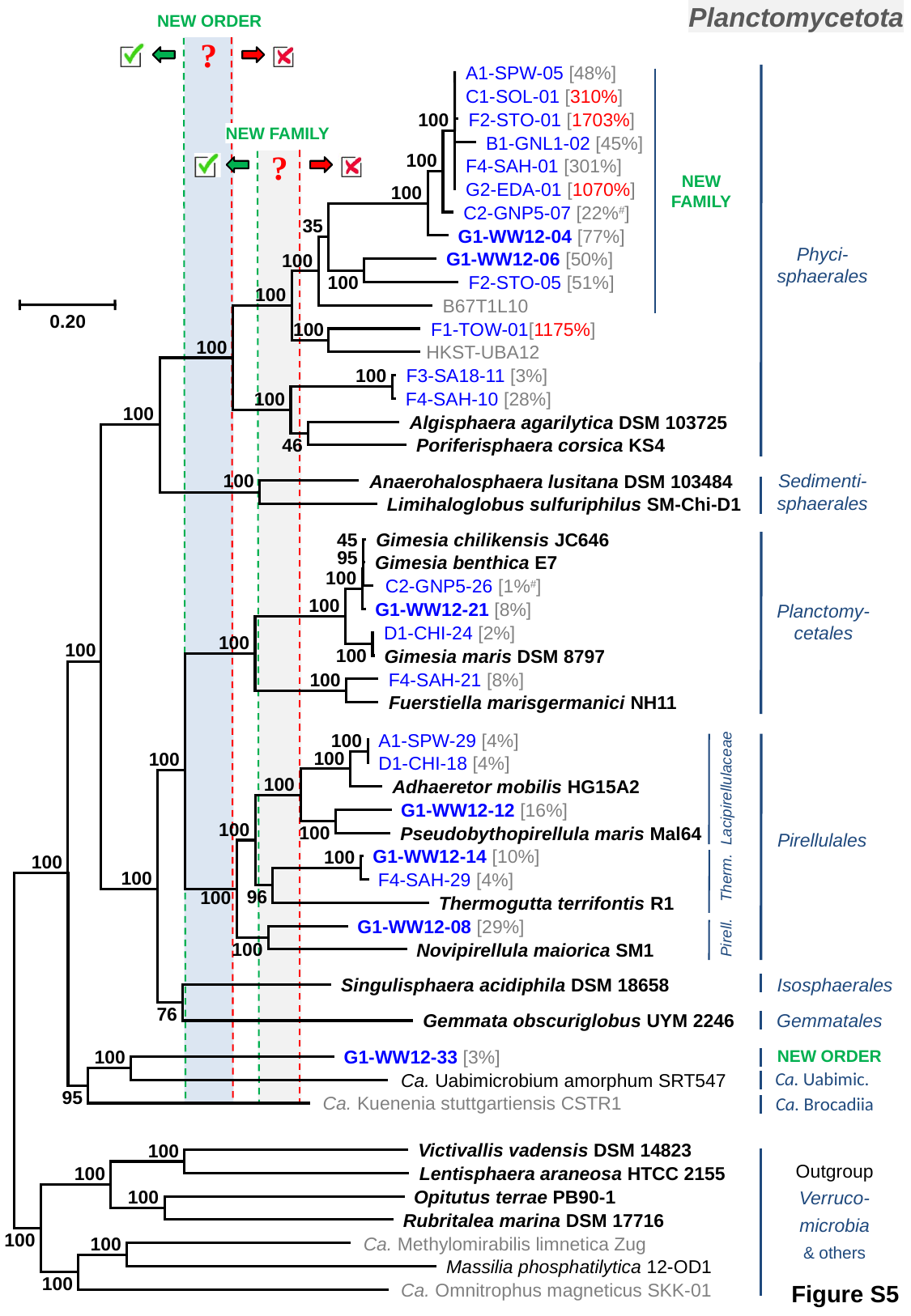

Planctomycetota
NEW ORDER
?
 A1-SPW-05 [48%]
 C1-SOL-01 [310%]
 F2-STO-01 [1703%]
 B1-GNL1-02 [45%]
 F4-SAH-01 [301%]
 G2-EDA-01 [1070%]
 C2-GNP5-07 [22%#]
 G1-WW12-04 [77%]
 G1-WW12-06 [50%]
 F2-STO-05 [51%]
 B67T1L10
 F1-TOW-01[1175%]
HKST-UBA12
 F3-SA18-11 [3%]
 F4-SAH-10 [28%]
 Algisphaera agarilytica DSM 103725
 Poriferisphaera corsica KS4
 Anaerohalosphaera lusitana DSM 103484
 Limihaloglobus sulfuriphilus SM-Chi-D1
 Gimesia chilikensis JC646
 Gimesia benthica E7
 C2-GNP5-26 [1%#]
 G1-WW12-21 [8%]
 D1-CHI-24 [2%]
 Gimesia maris DSM 8797
 F4-SAH-21 [8%]
 Fuerstiella marisgermanici NH11
 A1-SPW-29 [4%]
 D1-CHI-18 [4%]
 Adhaeretor mobilis HG15A2
 G1-WW12-12 [16%]
 Pseudobythopirellula maris Mal64
 G1-WW12-14 [10%]
 F4-SAH-29 [4%]
 Thermogutta terrifontis R1
 G1-WW12-08 [29%]
 Novipirellula maiorica SM1
 Singulisphaera acidiphila DSM 18658
 Gemmata obscuriglobus UYM 2246
 G1-WW12-33 [3%]
 Ca. Uabimicrobium amorphum SRT547
 Ca. Kuenenia stuttgartiensis CSTR1
 Victivallis vadensis DSM 14823
 Lentisphaera araneosa HTCC 2155
 Opitutus terrae PB90-1
 Rubritalea marina DSM 17716
 Ca. Methylomirabilis limnetica Zug
 Massilia phosphatilytica 12-OD1
 Ca. Omnitrophus magneticus SKK-01
100
100
100
35
100
100
100
100
100
100
100
100
46
100
45
95
100
100
100
100
100
100
100
100
100
100
100
100
100
100
100
96
100
100
76
100
95
100
100
100
100
100
100
NEW FAMILY
?
NEW
FAMILY
Phyci-
sphaerales
0.20
Sedimenti-
sphaerales
Planctomy-
cetales
Lacipirellulaceae
Pirellulales
Therm.
Pirell.
Isosphaerales
Gemmatales
NEW ORDER
Ca. Uabimic.
Ca. Brocadiia
Outgroup
Verruco-
microbia
& others
 Figure S5

## Slide 4
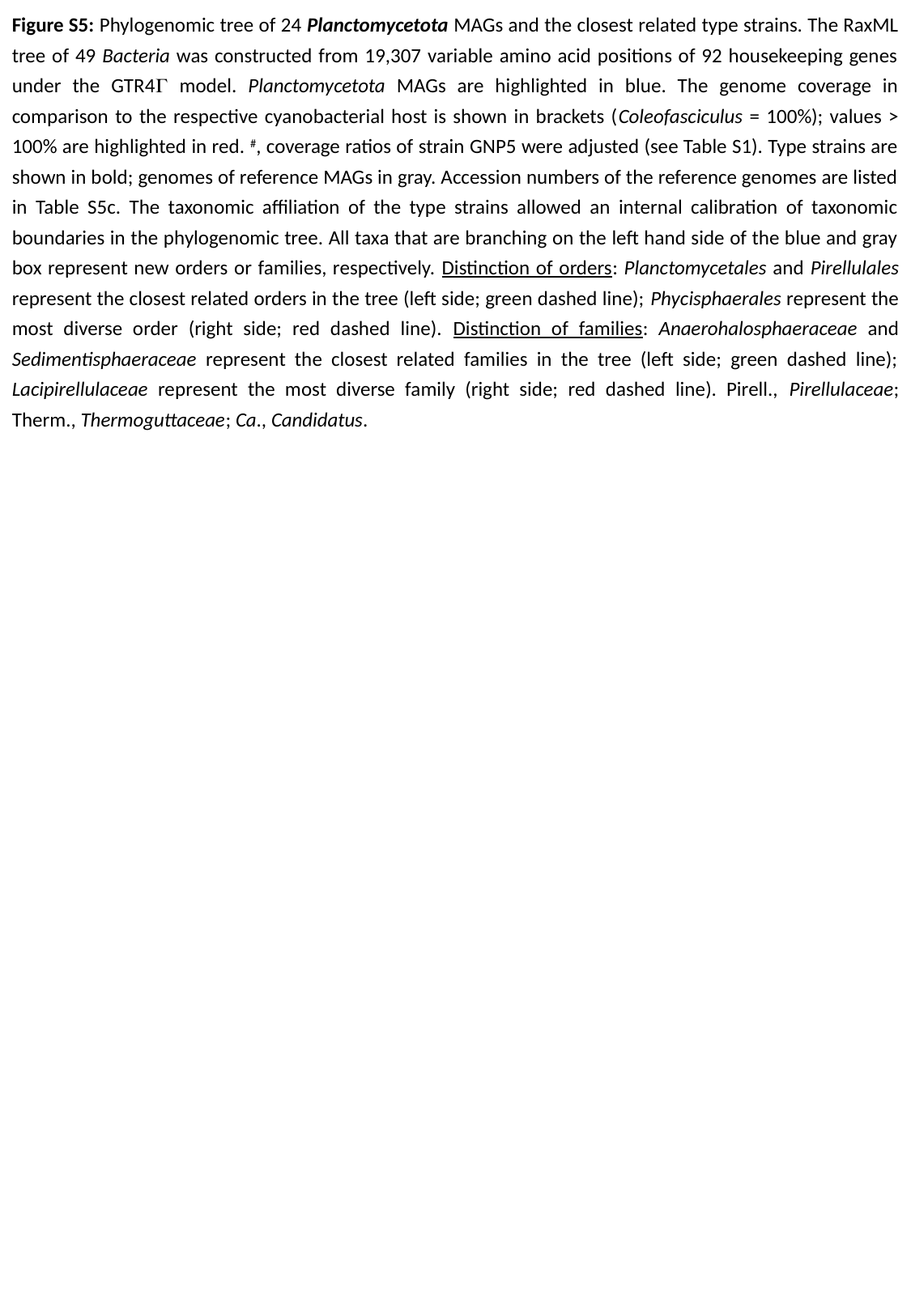

Figure S5: Phylogenomic tree of 24 Planctomycetota MAGs and the closest related type strains. The RaxML tree of 49 Bacteria was constructed from 19,307 variable amino acid positions of 92 housekeeping genes under the GTR4G model. Planctomycetota MAGs are highlighted in blue. The genome coverage in comparison to the respective cyanobacterial host is shown in brackets (Coleofasciculus = 100%); values > 100% are highlighted in red. #, coverage ratios of strain GNP5 were adjusted (see Table S1). Type strains are shown in bold; genomes of reference MAGs in gray. Accession numbers of the reference genomes are listed in Table S5c. The taxonomic affiliation of the type strains allowed an internal calibration of taxonomic boundaries in the phylogenomic tree. All taxa that are branching on the left hand side of the blue and gray box represent new orders or families, respectively. Distinction of orders: Planctomycetales and Pirellulales represent the closest related orders in the tree (left side; green dashed line); Phycisphaerales represent the most diverse order (right side; red dashed line). Distinction of families: Anaerohalosphaeraceae and Sedimentisphaeraceae represent the closest related families in the tree (left side; green dashed line); Lacipirellulaceae represent the most diverse family (right side; red dashed line). Pirell., Pirellulaceae; Therm., Thermoguttaceae; Ca., Candidatus.

## Slide 5
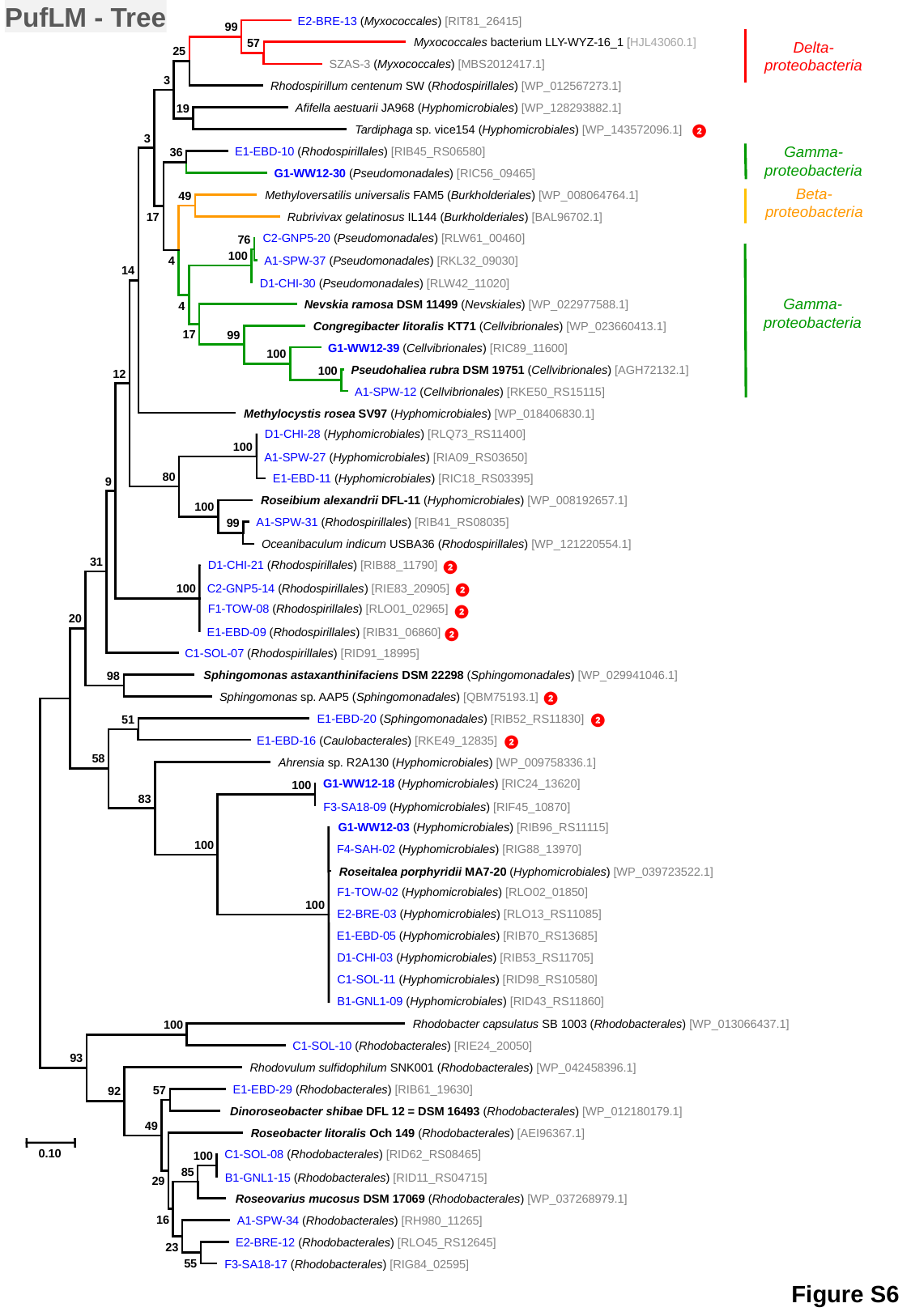

PufLM - Tree
E2-BRE-13 (Myxococcales) [RIT81_26415]
99
Myxococcales bacterium LLY-WYZ-16_1 [HJL43060.1]
57
Delta-
proteobacteria
25
SZAS-3 (Myxococcales) [MBS2012417.1]
3
Rhodospirillum centenum SW (Rhodospirillales) [WP_012567273.1]
Afifella aestuarii JA968 (Hyphomicrobiales) [WP_128293882.1]
19
❷
Tardiphaga sp. vice154 (Hyphomicrobiales) [WP_143572096.1]
3
Gamma-
proteobacteria
E1-EBD-10 (Rhodospirillales) [RIB45_RS06580]
36
G1-WW12-30 (Pseudomonadales) [RIC56_09465]
Beta-
proteobacteria
Methyloversatilis universalis FAM5 (Burkholderiales) [WP_008064764.1]
49
17
Rubrivivax gelatinosus IL144 (Burkholderiales) [BAL96702.1]
C2-GNP5-20 (Pseudomonadales) [RLW61_00460]
76
100
A1-SPW-37 (Pseudomonadales) [RKL32_09030]
4
14
D1-CHI-30 (Pseudomonadales) [RLW42_11020]
Gamma-
proteobacteria
Nevskia ramosa DSM 11499 (Nevskiales) [WP_022977588.1]
4
Congregibacter litoralis KT71 (Cellvibrionales) [WP_023660413.1]
17
99
G1-WW12-39 (Cellvibrionales) [RIC89_11600]
100
Pseudohaliea rubra DSM 19751 (Cellvibrionales) [AGH72132.1]
100
12
A1-SPW-12 (Cellvibrionales) [RKE50_RS15115]
Methylocystis rosea SV97 (Hyphomicrobiales) [WP_018406830.1]
D1-CHI-28 (Hyphomicrobiales) [RLQ73_RS11400]
100
A1-SPW-27 (Hyphomicrobiales) [RIA09_RS03650]
80
E1-EBD-11 (Hyphomicrobiales) [RIC18_RS03395]
9
Roseibium alexandrii DFL-11 (Hyphomicrobiales) [WP_008192657.1]
100
A1-SPW-31 (Rhodospirillales) [RIB41_RS08035]
99
Oceanibaculum indicum USBA36 (Rhodospirillales) [WP_121220554.1]
❷
31
D1-CHI-21 (Rhodospirillales) [RIB88_11790]
❷
C2-GNP5-14 (Rhodospirillales) [RIE83_20905]
100
❷
F1-TOW-08 (Rhodospirillales) [RLO01_02965]
20
❷
E1-EBD-09 (Rhodospirillales) [RIB31_06860]
C1-SOL-07 (Rhodospirillales) [RID91_18995]
Sphingomonas astaxanthinifaciens DSM 22298 (Sphingomonadales) [WP_029941046.1]
98
❷
Sphingomonas sp. AAP5 (Sphingomonadales) [QBM75193.1]
❷
E1-EBD-20 (Sphingomonadales) [RIB52_RS11830]
51
❷
E1-EBD-16 (Caulobacterales) [RKE49_12835]
58
Ahrensia sp. R2A130 (Hyphomicrobiales) [WP_009758336.1]
G1-WW12-18 (Hyphomicrobiales) [RIC24_13620]
100
83
F3-SA18-09 (Hyphomicrobiales) [RIF45_10870]
G1-WW12-03 (Hyphomicrobiales) [RIB96_RS11115]
100
F4-SAH-02 (Hyphomicrobiales) [RIG88_13970]
Roseitalea porphyridii MA7-20 (Hyphomicrobiales) [WP_039723522.1]
F1-TOW-02 (Hyphomicrobiales) [RLO02_01850]
100
E2-BRE-03 (Hyphomicrobiales) [RLO13_RS11085]
E1-EBD-05 (Hyphomicrobiales) [RIB70_RS13685]
D1-CHI-03 (Hyphomicrobiales) [RIB53_RS11705]
C1-SOL-11 (Hyphomicrobiales) [RID98_RS10580]
B1-GNL1-09 (Hyphomicrobiales) [RID43_RS11860]
Rhodobacter capsulatus SB 1003 (Rhodobacterales) [WP_013066437.1]
100
C1-SOL-10 (Rhodobacterales) [RIE24_20050]
93
Rhodovulum sulfidophilum SNK001 (Rhodobacterales) [WP_042458396.1]
E1-EBD-29 (Rhodobacterales) [RIB61_19630]
57
92
Dinoroseobacter shibae DFL 12 = DSM 16493 (Rhodobacterales) [WP_012180179.1]
49
Roseobacter litoralis Och 149 (Rhodobacterales) [AEI96367.1]
0.10
C1-SOL-08 (Rhodobacterales) [RID62_RS08465]
100
85
B1-GNL1-15 (Rhodobacterales) [RID11_RS04715]
29
Roseovarius mucosus DSM 17069 (Rhodobacterales) [WP_037268979.1]
16
A1-SPW-34 (Rhodobacterales) [RH980_11265]
E2-BRE-12 (Rhodobacterales) [RLO45_RS12645]
23
55
F3-SA18-17 (Rhodobacterales) [RIG84_02595]
 Figure S6

## Slide 6
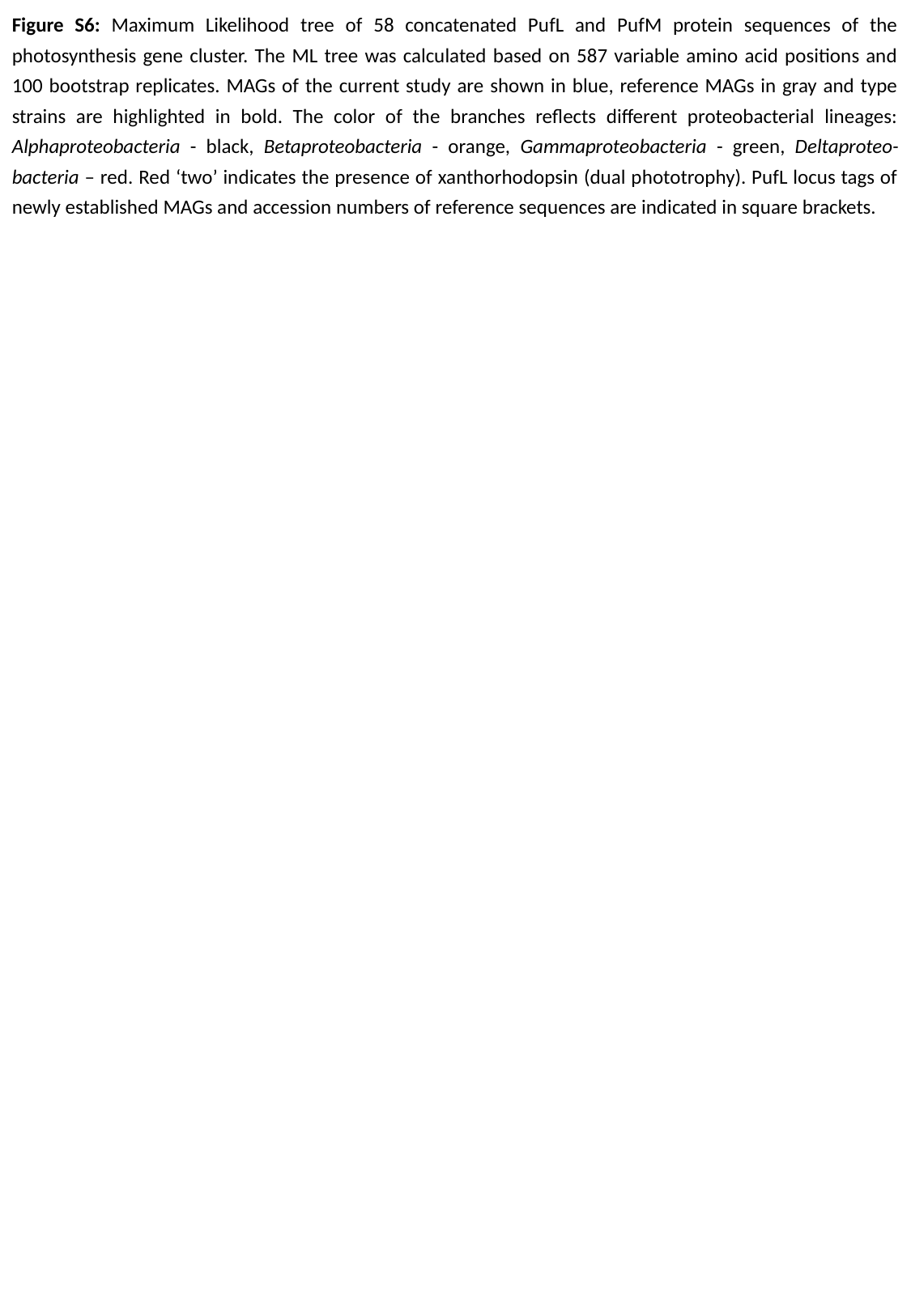

Figure S6: Maximum Likelihood tree of 58 concatenated PufL and PufM protein sequences of the photosynthesis gene cluster. The ML tree was calculated based on 587 variable amino acid positions and 100 bootstrap replicates. MAGs of the current study are shown in blue, reference MAGs in gray and type strains are highlighted in bold. The color of the branches reflects different proteobacterial lineages: Alphaproteobacteria - black, Betaproteobacteria - orange, Gammaproteobacteria - green, Deltaproteo-bacteria – red. Red ‘two’ indicates the presence of xanthorhodopsin (dual phototrophy). PufL locus tags of newly established MAGs and accession numbers of reference sequences are indicated in square brackets.

## Slide 7
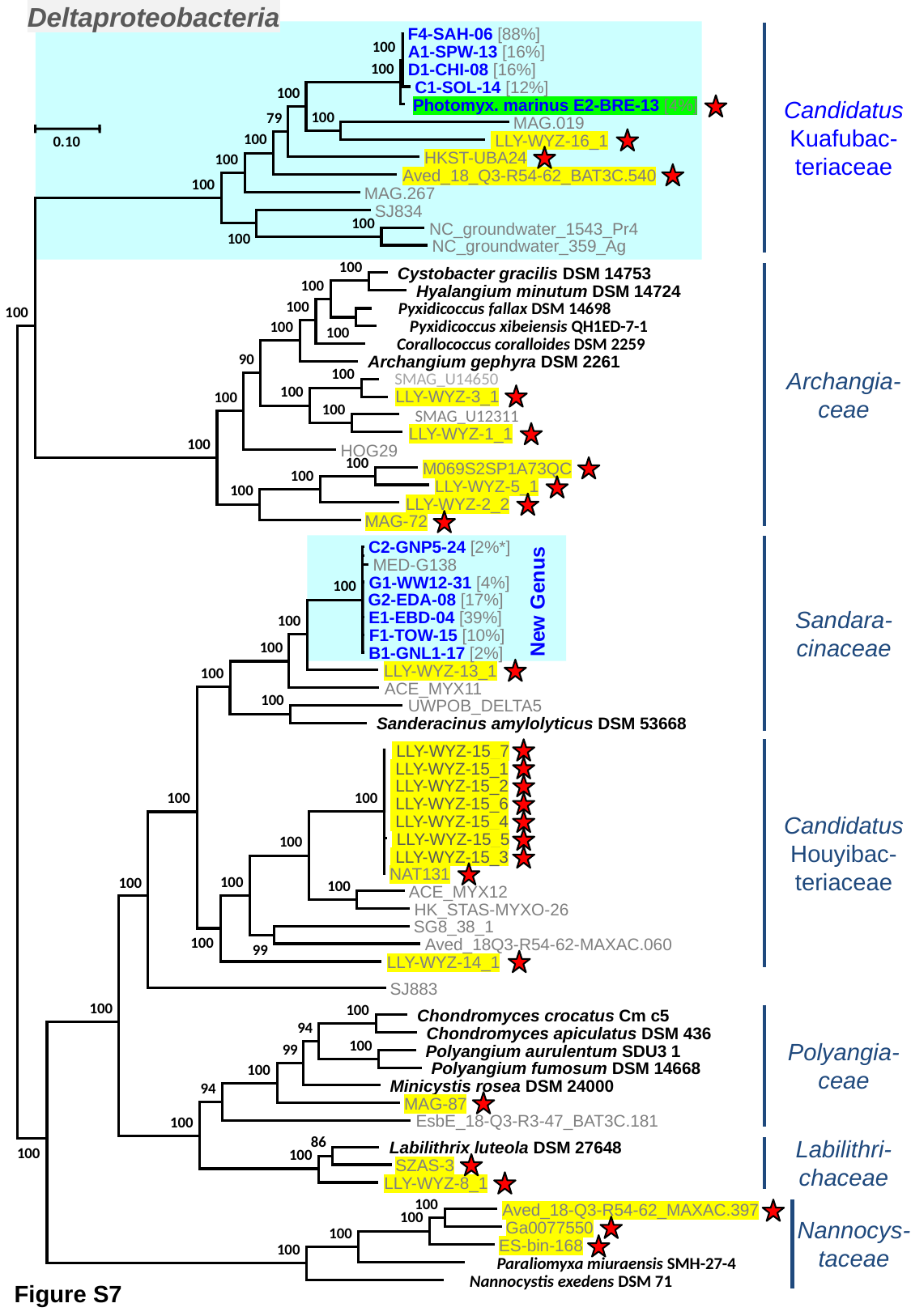

Deltaproteobacteria
F4-SAH-06 [88%]
A1-SPW-13 [16%]
D1-CHI-08 [16%]
C1-SOL-14 [12%]
Photomyx. marinus E2-BRE-13 [4%]
MAG.019
 LLY-WYZ-16_1
HKST-UBA24
Aved_18_Q3-R54-62_BAT3C.540
MAG.267
SJ834
NC_groundwater_1543_Pr4
NC_groundwater_359_Ag
 Cystobacter gracilis DSM 14753
 Hyalangium minutum DSM 14724
 Pyxidicoccus fallax DSM 14698
 Pyxidicoccus xibeiensis QH1ED-7-1
 Corallococcus coralloides DSM 2259
 Archangium gephyra DSM 2261
 SMAG_U14650
LLY-WYZ-3_1
 SMAG_U12311
LLY-WYZ-1_1
HOG29
M069S2SP1A73QC
LLY-WYZ-5_1
LLY-WYZ-2_2
MAG-72
C2-GNP5-24 [2%*]
MED-G138
G1-WW12-31 [4%]
G2-EDA-08 [17%]
E1-EBD-04 [39%]
F1-TOW-15 [10%]
B1-GNL1-17 [2%]
LLY-WYZ-13_1
ACE_MYX11
UWPOB_DELTA5
 Sanderacinus amylolyticus DSM 53668
 LLY-WYZ-15_7
 LLY-WYZ-15_1
 LLY-WYZ-15_2
 LLY-WYZ-15_6
 LLY-WYZ-15_4
 LLY-WYZ-15_5
 LLY-WYZ-15_3
NAT131
ACE_MYX12
HK_STAS-MYXO-26
SG8_38_1
Aved_18Q3-R54-62-MAXAC.060
LLY-WYZ-14_1
SJ883
 Chondromyces crocatus Cm c5
 Chondromyces apiculatus DSM 436
 Polyangium aurulentum SDU3 1
 Polyangium fumosum DSM 14668
 Minicystis rosea DSM 24000
MAG-87
EsbE_18-Q3-R3-47_BAT3C.181
 Labilithrix luteola DSM 27648
SZAS-3
LLY-WYZ-8_1
Aved_18-Q3-R54-62_MAXAC.397
Ga0077550
ES-bin-168
 Paraliomyxa miuraensis SMH-27-4
 Nannocystis exedens DSM 71
Candidatus
Kuafubac-
teriaceae
Archangia-
ceae
Sandara-
cinaceae
Candidatus
Houyibac-teriaceae
Polyangia-
ceae
Labilithri-
chaceae
Nannocys-
taceae
100
100
100
100
79
100
100
100
100
100
100
100
100
100
100
100
90
100
100
100
100
100
100
100
100
100
100
100
100
100
100
100
100
100
100
100
100
99
100
100
94
100
99
100
94
100
86
100
100
100
100
100
100
New Genus
0.10
 Figure S7

## Slide 8
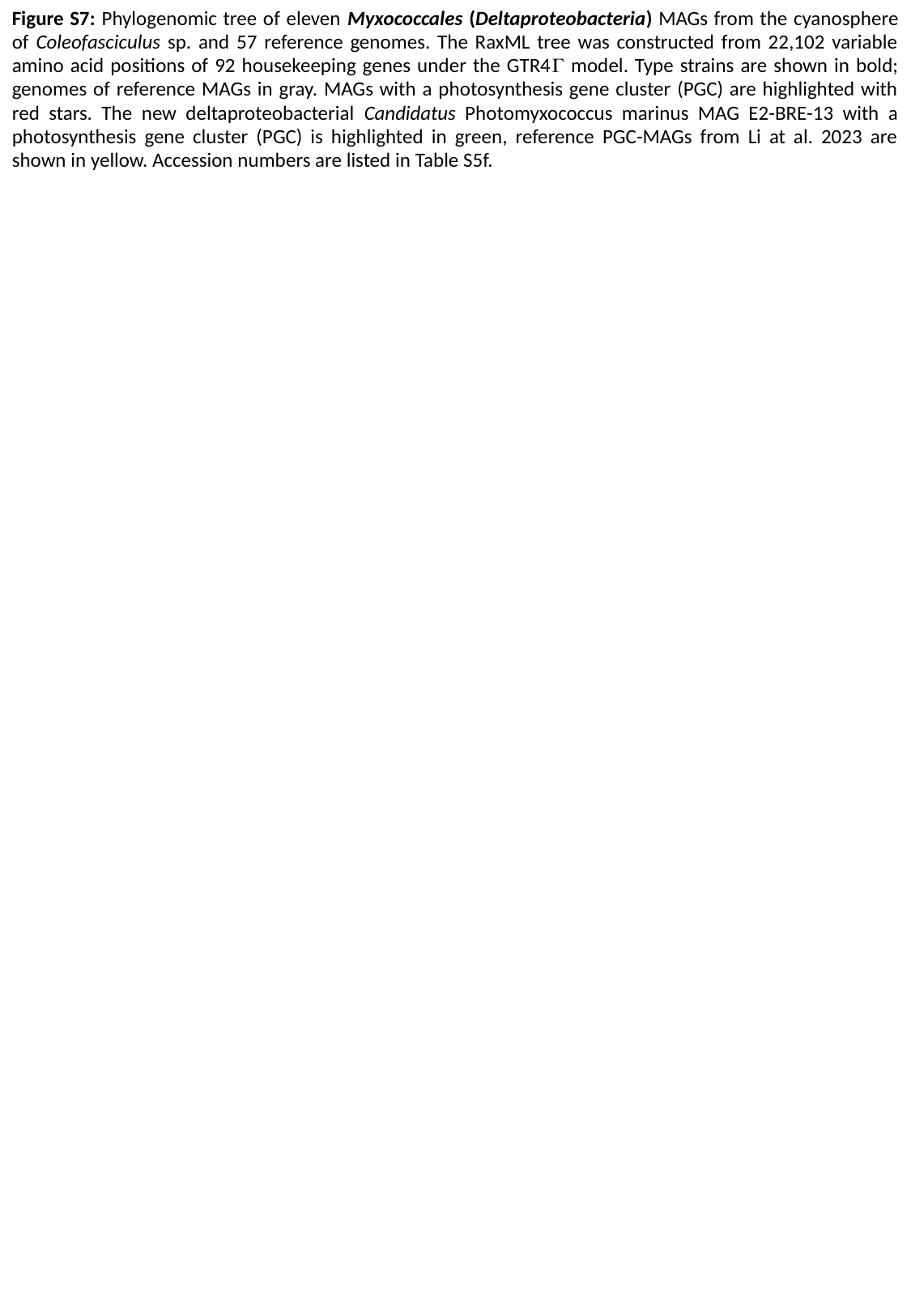

Figure S7: Phylogenomic tree of eleven Myxococcales (Deltaproteobacteria) MAGs from the cyanosphere of Coleofasciculus sp. and 57 reference genomes. The RaxML tree was constructed from 22,102 variable amino acid positions of 92 housekeeping genes under the GTR4G model. Type strains are shown in bold; genomes of reference MAGs in gray. MAGs with a photosynthesis gene cluster (PGC) are highlighted with red stars. The new deltaproteobacterial Candidatus Photomyxococcus marinus MAG E2-BRE-13 with a photosynthesis gene cluster (PGC) is highlighted in green, reference PGC-MAGs from Li at al. 2023 are shown in yellow. Accession numbers are listed in Table S5f.

## Slide 9
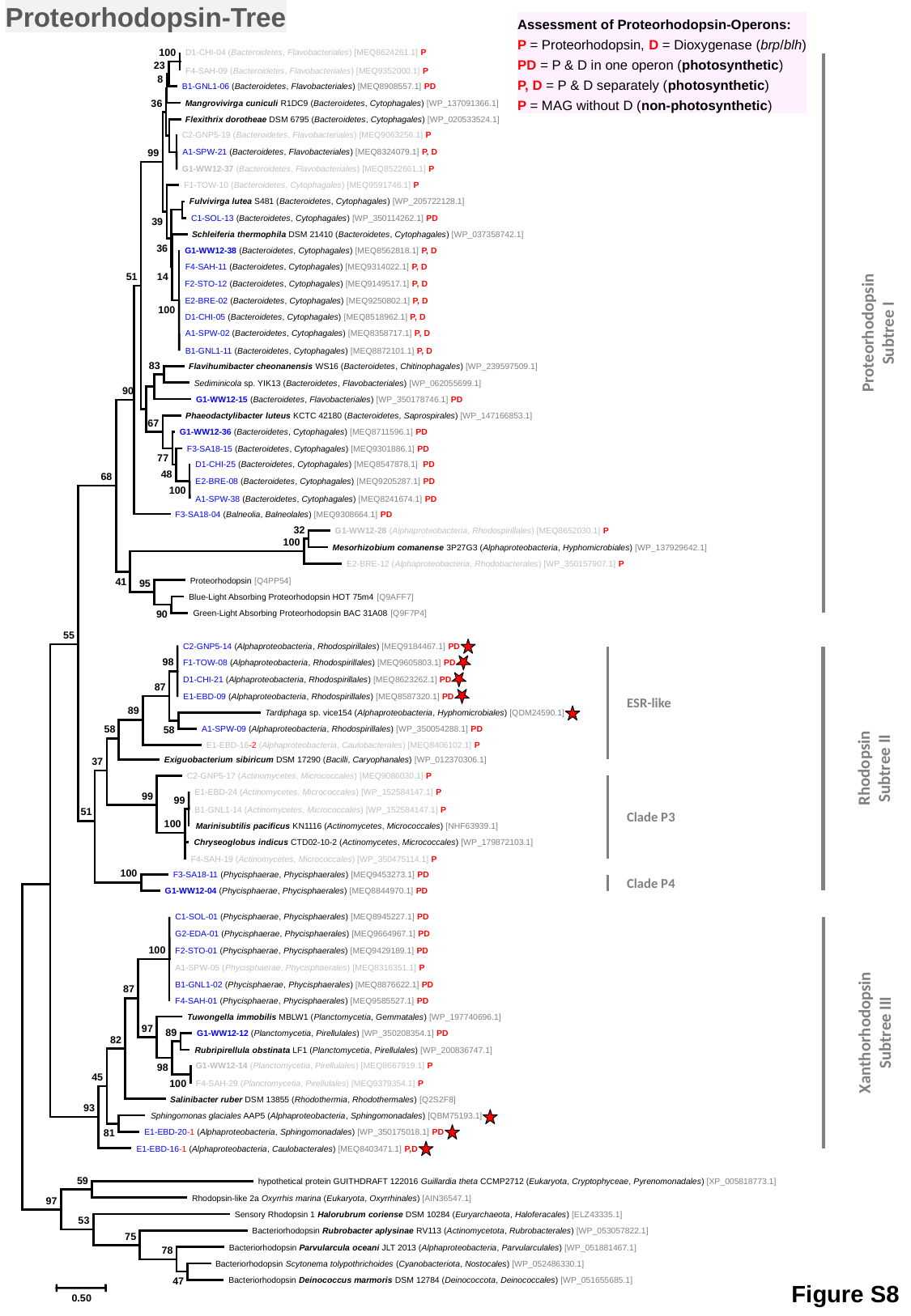

Proteorhodopsin-Tree
Assessment of Proteorhodopsin-Operons:
P = Proteorhodopsin, D = Dioxygenase (brp/blh)
PD = P & D in one operon (photosynthetic)
P, D = P & D separately (photosynthetic)
P = MAG without D (non-photosynthetic)
100
23
8
99
39
51
100
83
90
67
77
48
68
100
32
100
41
95
90
D1-CHI-04 (Bacteroidetes, Flavobacteriales) [MEQ8624261.1] P
F4-SAH-09 (Bacteroidetes, Flavobacteriales) [MEQ9352000.1] P
B1-GNL1-06 (Bacteroidetes, Flavobacteriales) [MEQ8908557.1] PD
36
Mangrovivirga cuniculi R1DC9 (Bacteroidetes, Cytophagales) [WP_137091366.1]
Flexithrix dorotheae DSM 6795 (Bacteroidetes, Cytophagales) [WP_020533524.1]
C2-GNP5-19 (Bacteroidetes, Flavobacteriales) [MEQ9063256.1] P
A1-SPW-21 (Bacteroidetes, Flavobacteriales) [MEQ8324079.1] P, D
G1-WW12-37 (Bacteroidetes, Flavobacteriales) [MEQ8522661.1] P
F1-TOW-10 (Bacteroidetes, Cytophagales) [MEQ9591746.1] P
Fulvivirga lutea S481 (Bacteroidetes, Cytophagales) [WP_205722128.1]
C1-SOL-13 (Bacteroidetes, Cytophagales) [WP_350114262.1] PD
Schleiferia thermophila DSM 21410 (Bacteroidetes, Cytophagales) [WP_037358742.1]
36
G1-WW12-38 (Bacteroidetes, Cytophagales) [MEQ8562818.1] P, D
F4-SAH-11 (Bacteroidetes, Cytophagales) [MEQ9314022.1] P, D
14
F2-STO-12 (Bacteroidetes, Cytophagales) [MEQ9149517.1] P, D
E2-BRE-02 (Bacteroidetes, Cytophagales) [MEQ9250802.1] P, D
Proteorhodopsin
Subtree I
D1-CHI-05 (Bacteroidetes, Cytophagales) [MEQ8518962.1] P, D
A1-SPW-02 (Bacteroidetes, Cytophagales) [MEQ8358717.1] P, D
B1-GNL1-11 (Bacteroidetes, Cytophagales) [MEQ8872101.1] P, D
Flavihumibacter cheonanensis WS16 (Bacteroidetes, Chitinophagales) [WP_239597509.1]
Sediminicola sp. YIK13 (Bacteroidetes, Flavobacteriales) [WP_062055699.1]
G1-WW12-15 (Bacteroidetes, Flavobacteriales) [WP_350178746.1] PD
Phaeodactylibacter luteus KCTC 42180 (Bacteroidetes, Saprospirales) [WP_147166853.1]
G1-WW12-36 (Bacteroidetes, Cytophagales) [MEQ8711596.1] PD
F3-SA18-15 (Bacteroidetes, Cytophagales) [MEQ9301886.1] PD
D1-CHI-25 (Bacteroidetes, Cytophagales) [MEQ8547878.1] PD
E2-BRE-08 (Bacteroidetes, Cytophagales) [MEQ9205287.1] PD
A1-SPW-38 (Bacteroidetes, Cytophagales) [MEQ8241674.1] PD
F3-SA18-04 (Balneolia, Balneolales) [MEQ9308664.1] PD
G1-WW12-28 (Alphaproteobacteria, Rhodospirillales) [MEQ8652030.1] P
Mesorhizobium comanense 3P27G3 (Alphaproteobacteria, Hyphomicrobiales) [WP_137929642.1]
E2-BRE-12 (Alphaproteobacteria, Rhodobacterales) [WP_350157907.1] P
Proteorhodopsin [Q4PP54]
Blue-Light Absorbing Proteorhodopsin HOT 75m4 [Q9AFF7]
Green-Light Absorbing Proteorhodopsin BAC 31A08 [Q9F7P4]
55
C2-GNP5-14 (Alphaproteobacteria, Rhodospirillales) [MEQ9184467.1] PD
ESR-like
Clade P3
Clade P4
98
F1-TOW-08 (Alphaproteobacteria, Rhodospirillales) [MEQ9605803.1] PD
D1-CHI-21 (Alphaproteobacteria, Rhodospirillales) [MEQ8623262.1] PD
87
E1-EBD-09 (Alphaproteobacteria, Rhodospirillales) [MEQ8587320.1] PD
89
Tardiphaga sp. vice154 (Alphaproteobacteria, Hyphomicrobiales) [QDM24590.1]
58
A1-SPW-09 (Alphaproteobacteria, Rhodospirillales) [WP_350054288.1] PD
58
E1-EBD-16-2 (Alphaproteobacteria, Caulobacterales) [MEQ8406102.1] P
Rhodopsin
Subtree II
Exiguobacterium sibiricum DSM 17290 (Bacilli, Caryophanales) [WP_012370306.1]
37
C2-GNP5-17 (Actinomycetes, Micrococcales) [MEQ9086030.1] P
E1-EBD-24 (Actinomycetes, Micrococcales) [WP_152584147.1] P
99
99
B1-GNL1-14 (Actinomycetes, Micrococcales) [WP_152584147.1] P
51
100
Marinisubtilis pacificus KN1116 (Actinomycetes, Micrococcales) [NHF63939.1]
Chryseoglobus indicus CTD02-10-2 (Actinomycetes, Micrococcales) [WP_179872103.1]
F4-SAH-19 (Actinomycetes, Micrococcales) [WP_350475114.1] P
100
F3-SA18-11 (Phycisphaerae, Phycisphaerales) [MEQ9453273.1] PD
G1-WW12-04 (Phycisphaerae, Phycisphaerales) [MEQ8844970.1] PD
C1-SOL-01 (Phycisphaerae, Phycisphaerales) [MEQ8945227.1] PD
G2-EDA-01 (Phycisphaerae, Phycisphaerales) [MEQ9664967.1] PD
100
F2-STO-01 (Phycisphaerae, Phycisphaerales) [MEQ9429189.1] PD
A1-SPW-05 (Phycisphaerae, Phycisphaerales) [MEQ8316351.1] P
B1-GNL1-02 (Phycisphaerae, Phycisphaerales) [MEQ8876622.1] PD
87
F4-SAH-01 (Phycisphaerae, Phycisphaerales) [MEQ9585527.1] PD
Xanthorhodopsin
Subtree III
Tuwongella immobilis MBLW1 (Planctomycetia, Gemmatales) [WP_197740696.1]
97
89
G1-WW12-12 (Planctomycetia, Pirellulales) [WP_350208354.1] PD
82
Rubripirellula obstinata LF1 (Planctomycetia, Pirellulales) [WP_200836747.1]
G1-WW12-14 (Planctomycetia, Pirellulales) [MEQ8667919.1] P
98
45
100
F4-SAH-29 (Planctomycetia, Pirellulales) [MEQ9379354.1] P
Salinibacter ruber DSM 13855 (Rhodothermia, Rhodothermales) [Q2S2F8]
93
Sphingomonas glaciales AAP5 (Alphaproteobacteria, Sphingomonadales) [QBM75193.1]
81
E1-EBD-20-1 (Alphaproteobacteria, Sphingomonadales) [WP_350175018.1] PD
E1-EBD-16-1 (Alphaproteobacteria, Caulobacterales) [MEQ8403471.1] P,D
59
97
53
75
78
47
hypothetical protein GUITHDRAFT 122016 Guillardia theta CCMP2712 (Eukaryota, Cryptophyceae, Pyrenomonadales) [XP_005818773.1]
Rhodopsin-like 2a Oxyrrhis marina (Eukaryota, Oxyrrhinales) [AIN36547.1]
Sensory Rhodopsin 1 Halorubrum coriense DSM 10284 (Euryarchaeota, Haloferacales) [ELZ43335.1]
Bacteriorhodopsin Rubrobacter aplysinae RV113 (Actinomycetota, Rubrobacterales) [WP_053057822.1]
Bacteriorhodopsin Parvularcula oceani JLT 2013 (Alphaproteobacteria, Parvularculales) [WP_051881467.1]
Bacteriorhodopsin Scytonema tolypothrichoides (Cyanobacteriota, Nostocales) [WP_052486330.1]
Bacteriorhodopsin Deinococcus marmoris DSM 12784 (Deinococcota, Deinococcales) [WP_051655685.1]
 Figure S8
0.50

## Slide 10
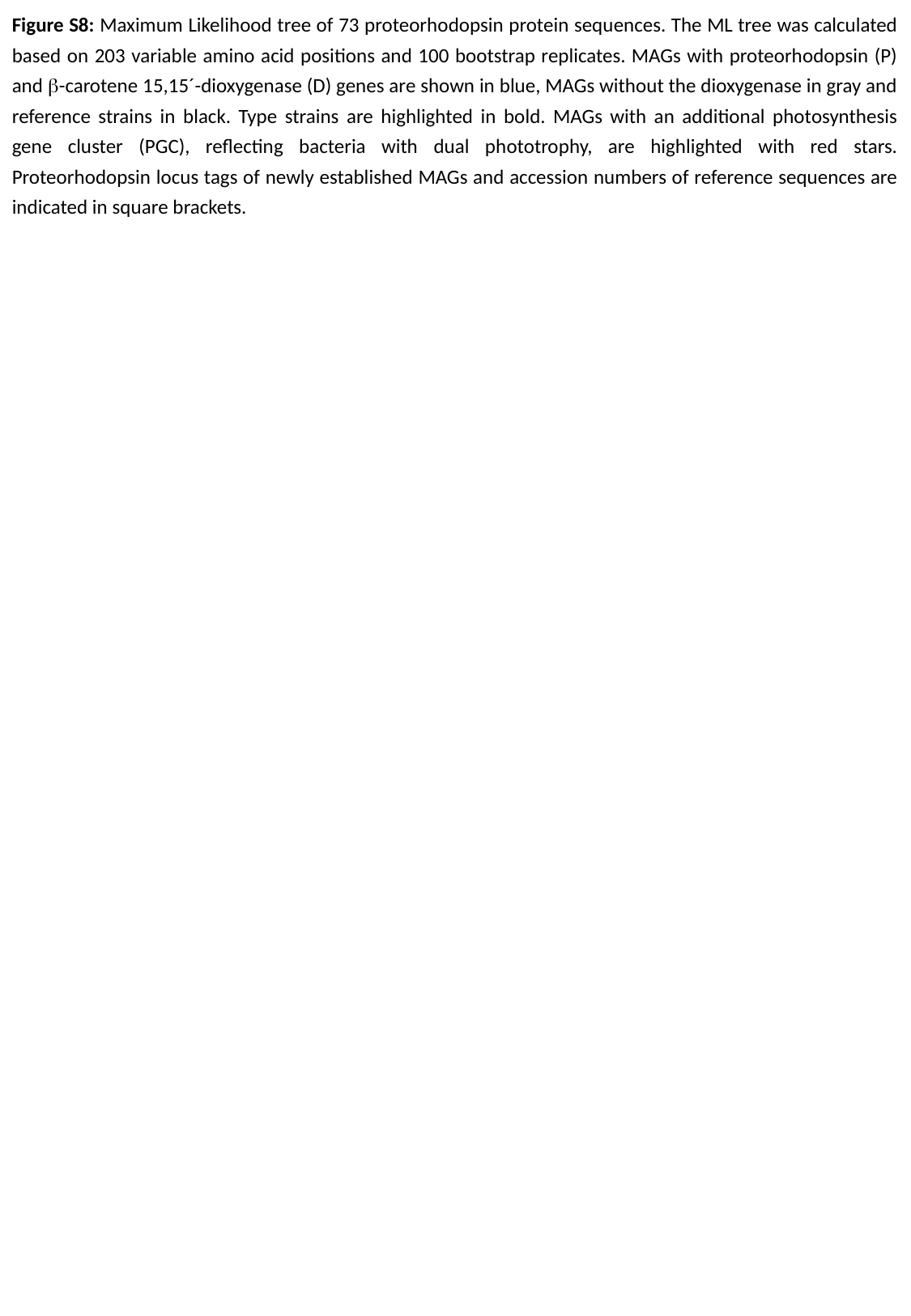

Figure S8: Maximum Likelihood tree of 73 proteorhodopsin protein sequences. The ML tree was calculated based on 203 variable amino acid positions and 100 bootstrap replicates. MAGs with proteorhodopsin (P) and b-carotene 15,15´-dioxygenase (D) genes are shown in blue, MAGs without the dioxygenase in gray and reference strains in black. Type strains are highlighted in bold. MAGs with an additional photosynthesis gene cluster (PGC), reflecting bacteria with dual phototrophy, are highlighted with red stars. Proteorhodopsin locus tags of newly established MAGs and accession numbers of reference sequences are indicated in square brackets.
