# Supplementary material for: The microbiome of marine mat-forming cyanobacteria—a microcosm of taxonomic novelty and phototrophic diversity
Source: ISME Commun. 2026 Feb 27;6(1):ycag041. doi: 10.1093/ismeco/ycag041 (PMC13043013; doi:10.1093/ismeco/ycag041)
Supplement: Text-S1_Coleofasciculus_260122_ycag041 [file text-s1_coleofasciculus_260122_ycag041.docx]

**Supplemental Text S1. [Results and Discussion]**

*The most common housemates of* Coleofasciculus

*Marinovum algicola* (*Alphaproteobacteria*, *Rhodobacterales*) was detected in twelve of 14 metagenomes (Figure 3a, Table S4a), and is thus the most common heterotrophic bacterium in the current study. The isolate WW12_PG-3A (= DSM 120483) represents the most abundant high-quality MAG of the *Coleofasciculus* sp. WW12 microbiome (G1-WW12-01; Table S1). Phylogenomic analyses of the MAGs with the closest related reference genomes revealed a largely unresolved positioning in the phylogenetic tree (Figure 3a, Figure S3, Table S5a), and the very close relationship is reflected by dDDH values between 90.2% and 99.8% (Table S2b). However, there is no hint for a bacterial cross-contamination of the *Coleofasciculus* cultures during up to three decades of continuous cultivation [1]. The presence of clearly distinguishable *M. algicola* MAGs is documented by (i) a common branching of F4-SAH-15 with strain DG1292 to the exclusion of all other MAGs grouping with the type strain DSM 10251^T^, and (ii) the distinct branch of C2-GNP5-01 and E1-EBD-02A reflecting their shared microevolution (Figure 3a). *Marinovum* was found in the microbiomes of all seven putative species of the genus *Coleofasciculus* (A - G; Figure 1c) and it shows an omnipresent global distribution among the nine investigated sampling sites (Figure 2). This finding expands the knowledge about the ecological niche of *M. algicola*, which was repeatedly isolated from the phycosphere of dinoflagellates [2, 3]. *Marinovum* strains are characterized by a multipartite genome organization and comprise a characteristic biofilm plasmid for surface attachment [4, 5]. Analogous to the abundance of *M. algicola* in the cyanosphere of *Coleofasciculus*, shotgun proteomics of the non-axenic harmful bloom-causing dinoflagellate *Pyrodinium bahamense* showed that most of the annotated proteins belonged to *M. algicola* [6].

*Roseitalea porphyridii* (*Alphaproteobacteria*, *Hyphomicrobiales*) was detected in eleven of 14 investigated metagenomes (Figure 3b, Table S4a), and is thus the second most common housemate. The reddish isolate WW12_G5-13 (= DSM 119668) represents another abundant MAG of the *Coleofasciculus* sp. WW12 microbiome, and the comparison of the genome coverage proposed a 1:1 ratio with the cyanobacterial host (G1-WW12-03; Table S1). The phylogenomic tree showed a common origin of the closely related MAGs (Figure 3b, Figure S4, Table S2c), which might reflect a specific adaptation to the ecological niche. The type strain *R. porphyridii* MA7-20^T^ that was isolated from the phycosphere of the unicellular red alga *Porphyridium marinum* produces carotenoid pigments [7], but the presence of a complete photosynthesis gene cluster (PGC) remained undetected in the species description (CP036532). Accordingly, the detection of conserved PGCs in all eleven MAGs of *R. porphyridii* is remarkable (Figure 3b, Table S6), as it provided clear evidence for the important role of aerobic anoxygenic photosynthesis for this species. The ASV- and MAG-based detection of the phototrophic alphaproteobacterium in 25 of 32 *Coleofasciculus* cultures (Table S4) likely reflects its commonality in the natural habitat, and its stable maintenance is probably supported by the light-dependent cultivation of the cyanobacterium.

**References**

1. Karsten U. Growth and organic osmolytes of geographically different isolates of Microcoleus chthonoplastes (Cyanobacteria) from benthic microbial mats: response to salinity change. *J Phycol* 1996; **32**: 501–506.

2. Lafay B, Ruimy R, De Traubenberg CR, Breittmayer V, Gauthier MJ, Christen R. Roseobacter algicola sp. nov., a new marine bacterium isolated from the phycosphere of the toxin-producing dinoflagellate Prorocentrum lima. *Int J Syst Bacteriol* 1995; **45**: 290–296.

3. Green DH, Llewellyn LE, Negri AP, Blackburn SI, Bolch CJS. Phylogenetic and functional diversity of the cultivable bacterial community associated with the paralytic shellfish poisoning dinoflagellate Gymnodinium catenatum. *FEMS Microbiol Ecol* 2004; **47**: 345–357.

4. Frank O, Göker M, Pradella S, Petersen J. Ocean’s Twelve: flagellar and biofilm chromids in the multipartite genome of *Marinovum algicola* DG898 exemplify functional compartmentalization. *Environ Microbiol* 2015; **17**: 4019–4034.

5. Pradella S, Päuker O, Petersen J. Genome organisation of the marine Roseobacter clade member *Marinovum algicola*. *Arch Microbiol* 2010; **192**: 115–126.

6. Subong BJJ, Malto ZBL, Lluisma AO, Azanza R V., Salvador-Reyes LA. Biochemical mapping of Pyrodinium bahamense unveils molecular underpinnings behind organismal processes. *Int J Mol Sci* 2021; **22**: 13332.

7. Hyeon JW, Jeong SE, Baek K, Jeon CO. Roseitalea porphyridii gen. Nov., sp. nov., isolated from a red alga, and reclassification of hoeflea suaedae Chung et al. 2013 as Pseudohoeflea suaedae gen. nov., comb. nov. *Int J Syst Evol Microbiol* 2017; **67**: 362–368.
